# Intelligent Recognition of Building Facade Defects: A Multilevel Review from Visual Perception to Engineering Operations and Maintenance

**DOI:** 10.3390/s26175498

**Published:** 2026-08-30

**Authors:** Jianhua Liu, Chen Li, Xinyu Wang, Bozhen Wang

**Affiliations:** Mobile Geospatial Big Data Cloud Service Innovation Team, Aerospace Remote Sensing Intelligent Computing Joint Laboratory, The School of Geomatics and Urban Spatial Informatics, Beijing University of Civil Engineering and Architecture, Beijing 100044, China; 2108570424083@stu.bucea.edu.cn (C.L.);

**Keywords:** building facade condition assessment, facade defect recognition, artificial intelligence, multimodal data fusion, digital twin

## Abstract

**Highlights:**

**What are the main findings?**

- A five-dimensional coding scheme—annotation granularity, task hierarchy, fusion level, spatial mapping, and engineering maturity—organizes evidence from 203 sources on building facade defect recognition.
- The main bottleneck is not individual-model accuracy but the missing link from defect detection and quantification to component-level repair decisions.

**What are the implications of the main findings?**

- Future evaluations should report recognition performance, deployment efficiency, engineering quantification error, and cross-domain performance degradation together.
- Standardized 2D-to-3D mapping-error protocols and engineering condition-rating frameworks are needed for large-scale deployment.

**Abstract:**

Building facades are continually exposed to weathering, material aging, hygrothermal cycling, and service-related disturbances. Cracks, delamination, spalling, and seepage can compromise durability and safety, while associated thermal anomalies provide indirect evidence of deterioration. Deep learning, UAV inspection, infrared thermography, LiDAR, building information modeling (BIM), and digital twins have shifted facade inspection toward automated sensing and spatially referenced assessment. Yet most studies remain focused on isolated measures of model accuracy and give limited attention to the full pathway from detection and quantification to component localization, condition rating, and repair. This review organizes the evidence along five dimensions: annotation granularity (A0–A2/A2*), visual task hierarchy (T1–T5), fusion level (F0–F3), spatial mapping level (S0–S3), and engineering maturity (M1–M3). The synthesis indicates that acquisition conditions, material heterogeneity, negative-sample composition, and annotation rules constrain model generalization. Single-modality methods cover classification, detection, segmentation, and partial geometric quantification, but facade-specific external validation remains limited. Multimodal gains depend on registration quality, thermophysical conditions, defect mechanisms, and sensor reliability. Mapping two-dimensional outputs to point clouds, BIM, and digital twins is technically feasible, but error propagation, component matching, and condition-rating protocols remain inconsistent. Interpretability tools and vision foundation models may support verification, annotation, and reporting but cannot replace dedicated systems with validated error bounds. Future work should prioritize cross-dataset benchmarks, facade-specific lightweight models, reproducible multimodal evaluation, standardized 2D-to-3D accuracy protocols, uncertainty-aware manual review, and engineering condition-rating frameworks.

## 1. Introduction

Building facades form the exterior envelope of existing buildings and remain exposed to structural loading, climatic action, material aging, and service-related disturbance throughout their service life. Cracks, delamination, finish detachment, water seepage, thermal anomalies, and reinforcement corrosion can reduce durability and safety and directly affect repair, strengthening, and operations and maintenance (O&M) decisions. China’s First National Comprehensive Natural Disaster Risk Survey reported approximately 128 billion m^2^ of building floor area, including about 66.2 billion m^2^ in urban areas [1]. A subsequent industry estimate indicated that approximately 35% of residential buildings were more than 30 years old [2]. A substantial proportion of the building stock is therefore entering a period of concentrated envelope maintenance. In late 2023, the Ministry of Housing and Urban-Rural Development incorporated structural-safety screening into its national guidance on urban physical examinations [3]. Such programs were subsequently introduced in 297 prefecture-level cities and more than 150 county-level cities [4]. National urban-renewal policy has further emphasized the resilience of existing buildings, the renewal of aging districts, and the use of urban physical-examination results in renewal decisions [5].

Visual surveys, hammer sounding, and close-range inspection from scaffolds or suspended platforms remain common facade-inspection practices. For high-rise buildings and large facade areas, however, these methods are labor-intensive, operator-dependent, difficult to scale, and potentially hazardous. UAV-based RGB imaging, infrared thermography, LiDAR, BIM, and digital twins have expanded the means of data acquisition and spatial documentation, while deep learning has reduced reliance on hand-crafted visual features [6,7,8,9]. The practical question has consequently shifted from whether a defect is visible to whether it can be detected consistently, measured reliably, associated with a building component, and converted into evidence for maintenance decisions.

Early studies increasingly moved from hand-crafted edge operators to CNN-based feature learning, classification, and object detection. Ali et al. documented the rapid growth of deep-learning-based crack detection between 2010 and 2022 [10]. Hamishebahar et al. surveyed the corresponding development of deep-learning methods for structural defect detection [11]. As pixel-level segmentation matured, multimodal sensing, structure-from-motion, point clouds, and BIM broadened the focus from coarse image localization to geometric description and spatial association. The review by Zhou et al. and the FIND dataset illustrate this shift toward pixel-level crack recognition in civil infrastructure [12]. More recent work has begun to connect visual outputs with physical sensing and maintenance decisions. Zhang et al.’s review of AI-assisted nondestructive testing illustrates why physically measured responses must be distinguished from conventional visual appearance [13].

Yet the transition from recognition to engineering use remains incomplete. Image classification supports rapid screening but provides little geometric information. Object detection offers approximate localization, although bounding boxes may contain substantial background for narrow or irregular defects. Semantic segmentation provides pixel-level contours but remains sensitive to annotation quality, acquisition conditions, and domain shift [11,12]. Limited public facade data, weak cross-dataset validation, complex field backgrounds, and high inference costs further constrain deployment [7,10,11,12]. Multimodal fusion also depends on registration accuracy, environmental conditions, and material response. Similarly, 3D visualization alone does not provide component identity, traceable geometric error, condition rating, or a repeatable model-updating process. What remains missing is a continuous and verifiable path from detection through quantification and spatial association to repair and O&M decisions.

Facade inspection also requires a clearly defined evidence boundary. Facade appearance varies with material, architectural texture, illumination, occlusion, imaging distance, and platform pose, while defect responses differ across sensing modalities. Spencer et al. distinguished visual-perception methods from physical-sensor approaches in their review of artificial intelligence for structural health monitoring, providing a useful basis for this boundary [14]. The present review focuses on visible or near-surface facade defects, including cracks, spalling, delamination, finish detachment, seepage, thermal anomalies, corrosion signs, and related material deterioration. Studies involving roads, bridges, tunnels, masonry, and general concrete surfaces are included only when their datasets, metrics, architectures, measurement methods, or 3D-mapping procedures are transferable; their reported performance is not treated as direct evidence of facade deployment [7,11,12,15].

Within this boundary, computer vision, nondestructive testing (NDT), and structural health monitoring (SHM) serve related but distinct functions. Computer vision addresses recognition, localization, segmentation, and measurement in images or video. NDT uses infrared thermography, radar, radiography, ultrasonics, and related techniques to investigate internal or near-surface conditions, whereas SHM emphasizes long-term sensing, condition assessment, and risk-informed decision-making. Reviews of AI-assisted NDT show that deep learning can improve the interpretation of radar, radiographic, and acoustic measurements, although these signals differ physically and structurally from RGB images [9,13]. NDT and SHM are therefore treated as adjacent or extended evidence domains rather than being merged with visual inspection into a single task class [16].

The review addresses these issues through three linked contributions.

(1) A five-dimensional analytical framework links annotation granularity, visual-task hierarchy, fusion level, spatial-mapping level, and engineering maturity. Its operational rules distinguish predictive performance from geometric quantification, component-level mapping, and closed-loop O&M capability.
(2) Corpus-wide evidence stratification separates direct facade studies from transferable infrastructure research, enabling methods, and contextual sources. The complete 203-source coding table and the quantitative analysis of the codable visual-technical subset provide a traceable basis for comparing task, spatial, maturity, and evidence-strength distributions.
(3) An engineering-oriented synthesis traces where information is lost between data acquisition and maintenance action, with particular attention to cross-domain generalization, modality registration, geometric error propagation, component association, and workflow integration. These gaps inform practical priorities for reporting and evaluation so that visual outputs can become calibrated, reviewable, and updateable engineering evidence.

The remainder of this paper is organized as follows. Section 2 describes the review methodology, coding framework, and evidence synthesis; Section 3 examines data acquisition, annotation, and datasets; Section 4 reviews recognition, multimodal fusion, quantitative measurement, 3D localization, and engineering implementation; and Section 5 presents the conclusions and research priorities.

## 2. Review Methodology and Evidence Organization

The reporting of this review follows PRISMA 2020 and the PRISMA-S extension for literature searches [17,18], subject to the historical-record limitations described in Section 2.1 and Section 5.2. A completed PRISMA 2020 checklist is provided as Supplementary File S1. Because building-facade defect recognition spans building pathology, computer vision, UAV inspection, infrared thermography, nondestructive testing, 3D reconstruction, BIM and digital twins, and structural health monitoring, this section defines the literature boundary, search scope, screening criteria, evidence-coding scheme, and evaluation framework used in the synthesis.

### 2.1. Search Scope, Screening Process, and Inclusion Criteria

The principal database search covered January 2015 through 27 April 2026, when the final database search was conducted. Targeted checks for policy and contextual sources were updated through 30 June 2026. Thirteen earlier sources were retained only when they provided foundational methods, benchmark datasets, standards, or essential background evidence within the broader methodological scope described by Koch et al. [15]. Information sources included Web of Science, Scopus, IEEE Xplore, ScienceDirect, SpringerLink, Wiley Online Library, MDPI, SAGE Journals, arXiv, CNKI, Crossref, and Google Scholar; reference lists of relevant reviews and representative primary studies were also screened.

The search combined three concept groups: (1) building facades and envelope systems; (2) cracks, delamination, debonding, spalling, seepage, thermal anomalies, and related defects; and (3) computer vision, deep learning, UAV inspection, infrared thermography, LiDAR, point clouds, BIM, digital twins, and 3D reconstruction. Table A1 summarizes the source roles, eligibility limits, and screening arrangements. Appendix A.2 presents the reconstructed master Boolean expression, while Table A2 records how its field wrapper and syntax were adapted for the principal databases and discovery platforms. The original source-specific search histories and retrieval counts were not fully retained; consequently, the reconstructed expressions document the search scope and logic but are not presented as verbatim historical queries.

Technical studies were eligible if they (1) addressed a building facade, envelope system, or transferable near-surface defect; (2) examined an automated recognition, sensing, multimodal-fusion, or spatial-mapping method relevant to facade inspection; and (3) reported sufficient methodological, data, metric, or experimental detail for qualitative synthesis. Policy documents, reporting guidelines, reviews, datasets, standards, and foundational methods were retained when they supplied necessary contextual or methodological evidence but were not treated as direct evidence of facade-recognition performance. Records were excluded if they concerned out-of-scope structures or defects, lacked a relevant automated recognition or spatial-mapping pipeline, examined only structural mechanisms or physical nondestructive testing, provided insufficient methodological or experimental information, or duplicated or had been superseded by a more complete publication.

**Figure 1:**
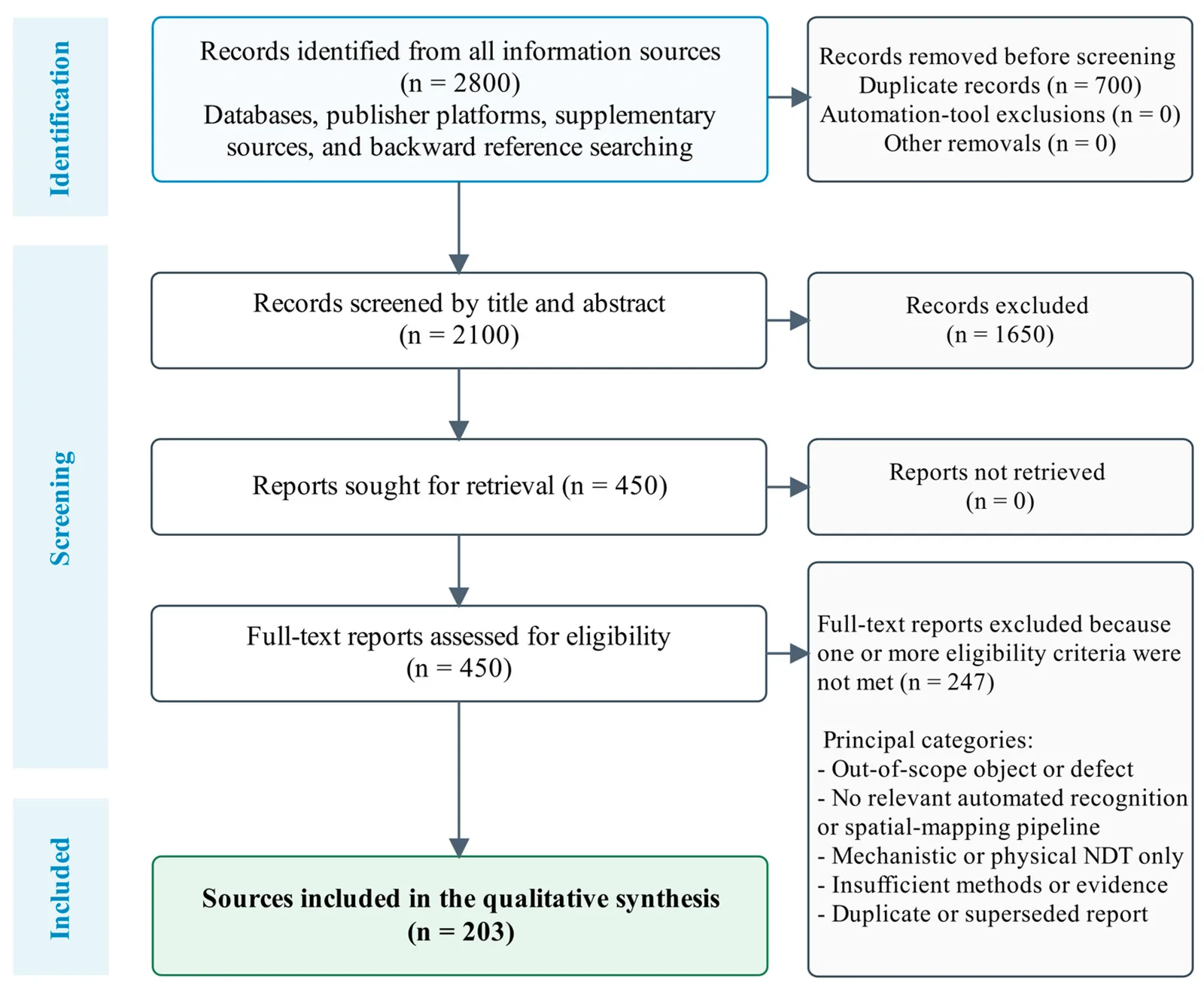
PRISMA 2020 flow diagram of the literature identification, screening, eligibility assessment, and inclusion process.

The search identified 2800 records. Removing 700 duplicates left 2100 records for title and abstract screening, of which 1650 were excluded. All 450 reports sought for retrieval were obtained and assessed in full text; 247 were excluded because they met one or more of the exclusion criteria described above. Because reason-specific historical counts were not retained, only the verified aggregate is reported. The final qualitative synthesis comprised 203 sources. Figure 1 summarizes the identification and selection process. One reviewer (C.L.) conducted title–abstract screening, full-text assessment, and data extraction; uncertain records and borderline inclusion decisions were discussed with X.W. and J.L. No automation tools were used for screening, eligibility assessment, or data extraction. The review was not registered, and no prospective protocol was prepared.

Data extraction and source-level coding were performed by C.L. using the framework defined in Section 2.2, Section 2.3 and Section 2.4. Recorded variables included study type, defect type and material, sensing modality, acquisition platform, annotation and task levels, validation design, originally reported performance metrics, and engineering evidence. Study authors were not contacted for clarification; information that was not reported was coded as NR rather than imputed.

**Figure 2:**
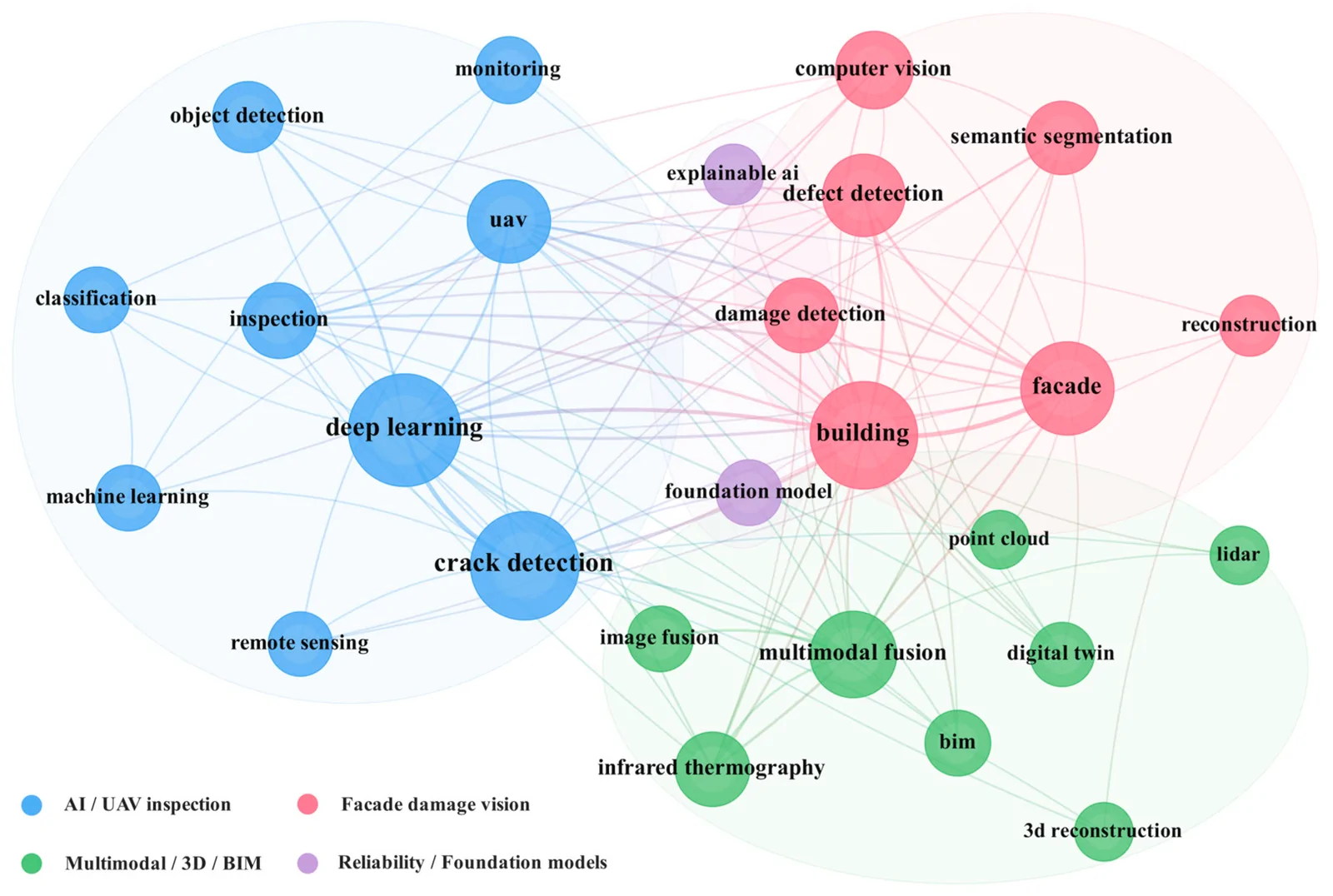
Keyword co-occurrence network and clustering analysis.

**Figure 3:**
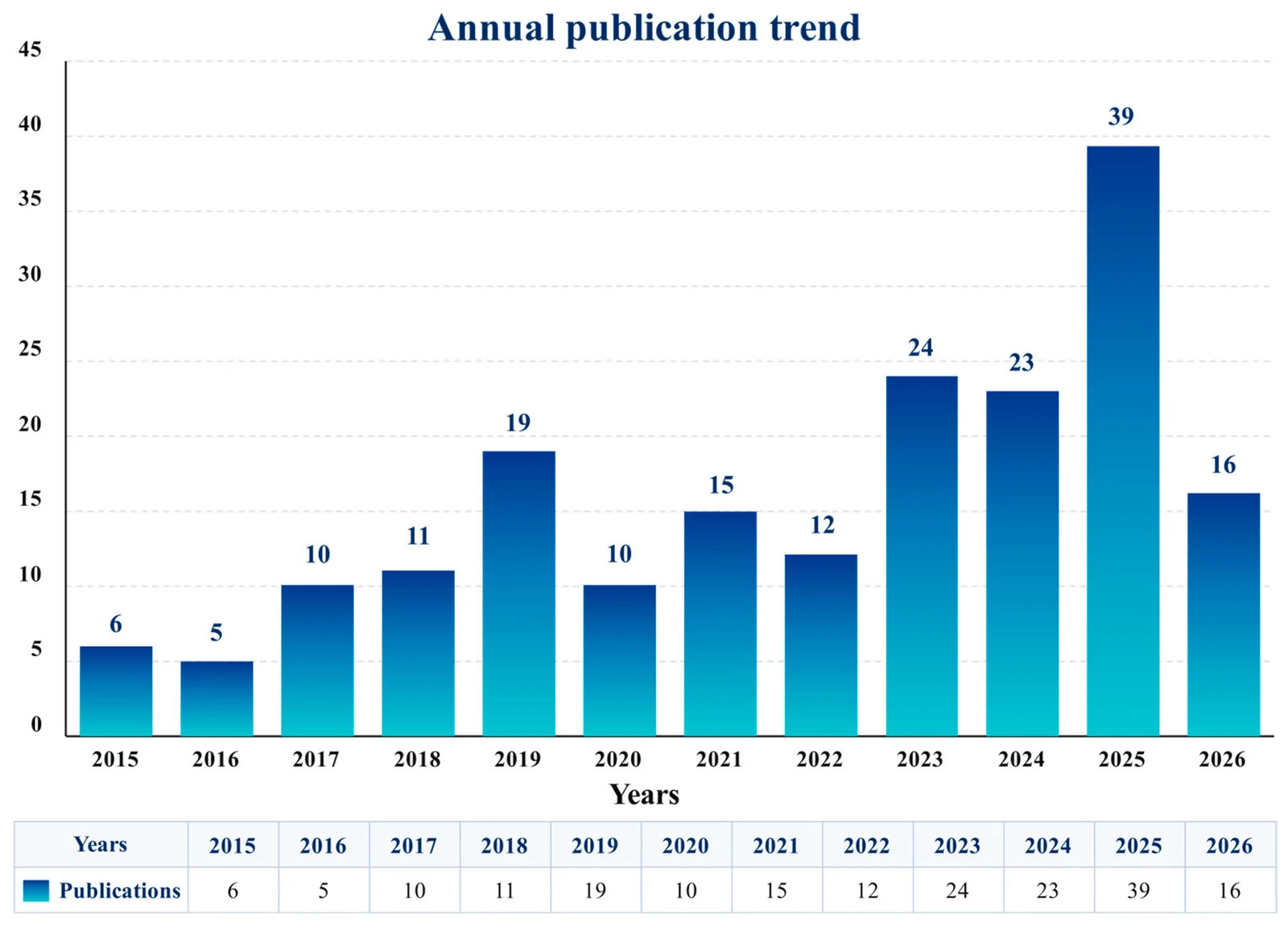
Annual distribution of the 190 retained sources published between 2015 and 2026; the 2026 count includes technical sources identified through 27 April 2026 and targeted policy or contextual updates through 30 June 2026 and therefore represents a partial-year total.

Figure 2 shows the principal thematic clusters in the retained literature, including UAV inspection, crack and defect detection, infrared thermography, deep-learning-based segmentation, and 3D/BIM mapping. Of the 203 retained sources, 190 were published between 2015 and 2026 and are plotted in Figure 3; the 13 pre-2015 sources were omitted from the temporal analysis. The 190-source corpus includes policy and contextual references [1,2,3,4,5], reporting guidelines [17,18], reviews, datasets, and primary studies. The annual series therefore characterizes the retained evidence base as a whole rather than only experimental facade-recognition studies and shows a marked increase in publication output in recent years. The 2026 count includes technical sources identified through 27 April 2026 and targeted policy or contextual updates through 30 June 2026 and therefore represents a partial year. Following completion of the formal screening and coding, six additional references [19,20,21,22,23,24] were identified through targeted supplementary searches to strengthen the methodological context. They were not incorporated into the original screened corpus of 203 sources and are therefore excluded from the A/T/F/S/M coding and the corpus-level analyses shown in Figure 2 and Figure 3.

### 2.2. Coding Scheme and Unified Analytical Dimensions

This paper adopts a unified coding scheme to organize the evidence rather than simply listing studies by author and year. Defect types include cracks, spalling, delamination/debonding, finish detachment, water seepage, thermal anomalies, corrosion signs, and compound defects; sensor modalities include RGB, infrared thermography, point clouds, GPR, ultrasonics, radiography, and multimodal combinations. Table 1 summarizes the annotation, task, fusion, spatial mapping, and engineering maturity levels used in the following sections.

Codes were assigned from the highest output actually demonstrated in the reported experiment or workflow, not from the intended application stated by the authors. A0 denotes image-level labels, A1 bounding boxes, and A2 pixel masks; A2* is used for dense point-level defect labels as the 3D analogue of a pixel mask. T1–T5 record the highest validated task output. T4 requires scale-aware geometric quantities such as length, width, area, displacement, or deformation rather than a segmentation mask alone; T5 requires localization or reconstruction in a 3D coordinate system or model.

Fusion codes describe where information streams interact. F0 denotes a single stream or multiple modalities assessed separately without an implemented fusion operation. F1 denotes fusion of co-registered raw inputs or images; F2 denotes learned interaction before the prediction head; and F3 denotes combination of predictions, temporal results, decisions, or independently derived measurements. Merely displaying RGB and IRT images side by side was not coded as fusion. Where a method implemented several fusion stages, the highest implemented interaction level was recorded, and the lower stages were retained in the coding note.

S1 was assigned when the result remained in image or thermogram coordinates. S2 required registration to a point cloud, mesh, reality model, or other metric 3D geometry. S3 required explicit binding to BIM or a digital-twin object or component; exporting a colored 3D visualization without a maintained component/model association remained S2. M1 denotes a proof of concept based mainly on laboratory data, public datasets, or a single technical demonstration. M2 requires controlled field acquisition or evaluation on real structures but does not require recurring O&M integration. M3 requires an integrated operational chain linking acquisition, recognition, spatial or component binding, human or rule-based review, maintenance decision support, and model/status updating. Field data alone therefore do not qualify a study as M3.

Contextual sources, reviews, general-purpose algorithms, and nonvisual physical-mechanism studies were retained in the evidence map but assigned N/A where A/T/F/S/M coding was not meaningful. NR in the evidence-strength fields means that an attribute was not reported or could not be verified; it is not interpreted as evidence that the attribute was absent. The complete source-level decisions and their verification basis are reported in Supplementary Table S1.

Together, the A/T/F/S/M codes distinguish predictive performance from spatial integration and operational maturity. High 2D accuracy at T3 does not, by itself, constitute S3 component-level BIM/digital-twin binding or M3 closed-loop deployment; similarly, F2 feature-level fusion does not demonstrate that the physical meaning of cross-modal evidence has been resolved.

### 2.3. Framework of Datasets, Platforms, and Evaluation Metrics

Data sources, acquisition platforms, and evaluation metrics form the basis of the method comparison. The sources include RGB images, infrared thermograms, RGB-IRT pairs, street-view or oblique-photogrammetry imagery, point-cloud/mesh/BIM data, and transferable crack data from roads, bridges, tunnels, and general concrete surfaces. Acquisition platforms include UAVs, ground cameras, street-view vehicles, wall-climbing robots, aerial oblique-photogrammetry systems, thermal cameras, and LiDAR. Because these platforms differ in imaging scale, occlusion, positioning capability, and engineering applicability, no model can be evaluated using a single accuracy metric. As shown in Table 2, evaluation should cover 2D recognition, deployment efficiency, geometric quantification, 3D localization, and generalization reliability. Classification, detection, and segmentation metrics describe recognition performance; efficiency metrics indicate edge deployability; geometric and 3D metrics assess spatial mapping; and generalization and uncertainty metrics indicate applicability across buildings, materials, and acquisition conditions.

### 2.4. Evidence Stratification, Appraisal, and Quantitative Synthesis

**Figure 4:**
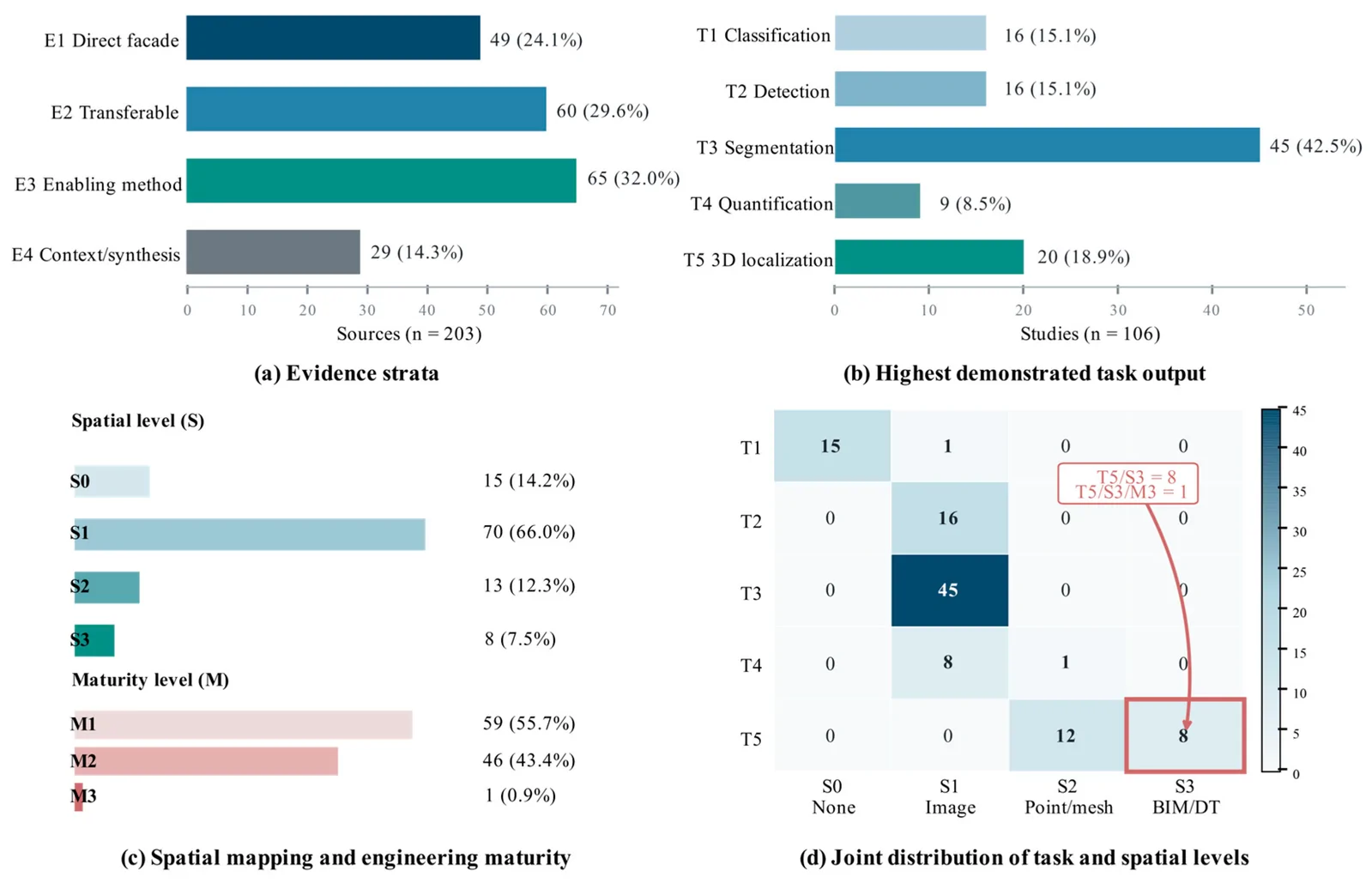
Corpus-wide evidence stratification and quantitative synthesis of the visual-technical subset. (**a**) Evidence strata across all 203 retained sources. (**b**) Highest demonstrated task level among the 106 codable visual-technical studies. (**c**) Spatial-mapping and engineering-maturity distributions in the same subset. (**d**) Cross-tabulation of task and spatial levels. The highlighted T5/S3 cell contains eight studies, all from the E1 direct-facade stratum; one also met the M3 criteria. Counts are descriptive, and NR evidence indicators are not interpreted as evidence of absence.

The synthesis combined corpus-level evidence stratification, source-level coding, and thematic comparison. For readability, Table 3 presents representative applications of the coding framework; it is illustrative rather than the basis of the quantitative results. Supplementary Table S1 reports the evidence stratum, A/T/F/S/M assignments, evidence-strength indicators, and verification basis for all 203 retained sources. Figure 4 summarizes the composition of the complete corpus and the quantitative profile of the codable visual-technical subset.

For corpus-level analysis, the 203 retained sources were classified into four evidence strata: E1 comprised 49 direct facade or building-envelope sources (24.1%); E2 comprised 60 transferable studies involving bridges, tunnels, roads, masonry, or general concrete surfaces (29.6%); E3 comprised 65 enabling algorithms, sensing methods, or other technical sources (32.0%); and E4 comprised 29 policy, reporting, review, standard, or contextual sources (14.3%) (Figure 4a). The quantitative A/T/F/S/M analysis used 106 codable visual-technical studies, including 47 E1 and 59 E2 sources. E3 and E4 sources, together with two E1 sources and one E2 source that did not implement a codable visual-recognition task, were retained for qualitative interpretation but excluded from this denominator. This separation prevents adjacent-domain and contextual evidence from being treated as direct evidence of facade-recognition performance or operational maturity.

Within each evidence stratum, sources were compared by defect type, sensing modality, visual task, and A/T/F/S/M level. Reported metrics were retained in their original definitions and units; missing values were neither imputed nor converted into common effect measures. Because the corpus combines heterogeneous datasets, algorithmic studies, field demonstrations, reviews, and contextual sources, neither a meta-analysis nor a single universal risk-of-bias instrument was applied. Evidence strength was instead profiled using six nonexclusive indicators: external validation, public data, public code, quantitative or geometric ground truth, cross-domain testing, and field evidence. An indicator was marked as verified only when supported by the source or an authoritative record; NR denotes information that was not reported or could not be verified, rather than confirmed absence. No formal analyses of statistical heterogeneity, sensitivity, reporting bias, or certainty of pooled effects were undertaken because no common estimand or pooled effect was defined.

As shown in Figure 4b–d, the 106-study visual-technical subset was concentrated at T3 segmentation (45 studies, 42.5%), followed by T5 3D localization/reconstruction (20, 18.9%), T1 classification (16, 15.1%), T2 detection (16, 15.1%), and T4 geometric quantification (9, 8.5%). Spatial outputs remained predominantly at S1 image coordinates (70, 66.0%); S0, S2, and S3 accounted for 15 (14.2%), 13 (12.3%), and 8 studies (7.5%), respectively. Engineering maturity was divided between M1 proof of concept (59, 55.7%) and M2 controlled field evaluation (46, 43.4%), while only one study (0.9%) satisfied the M3 operational-workflow criteria. The T-by-S cross-tabulation in Figure 4d identified eight T5/S3 studies, all of which belonged to the E1 direct-facade stratum; only one also reached M3. Thus, 3D or BIM association has become feasible as a technical demonstration, but integration with recurring inspection, component-level review, maintenance action, and model updating remains rare.

Evidence strength was profiled using six nonexclusive source-level indicators documented in Supplementary Table S1: external validation, public data, public code, quantitative or geometric ground truth, cross-domain testing, and field evidence. Within the 106-study visual-technical subset, 13 studies (12.3%) included verified external validation, 16 (15.1%) used or released public data, 3 (2.8%) had verified public code, 23 (21.7%) reported quantitative or geometric ground truth, 12 (11.3%) included cross-domain testing, and 47 (44.3%) used field evidence. These counts are verified minima: NR denotes that an attribute was not reported or could not be confirmed and must not be interpreted as evidence of absence. Field evidence should also not be equated with operational maturity; although 47 studies used field data, only one met the M3 criteria.

## 3. Data Acquisition, Annotation Schemes, and Benchmark-Dataset Challenges

Facade-defect recognition depends first on the conditions under which data are acquired. UAVs, ground cameras, street-view vehicles, wall-climbing robots, oblique-photogrammetry systems, and thermal cameras differ in imaging distance, viewing geometry, occlusion, illumination, surface reflectance, and positioning capability. If a defect is unresolved during acquisition or masked by the thermal background, later processing cannot reliably recover its boundary or engineering meaning. This section therefore treats data source, annotation granularity, negative samples, modality coverage, and generalization tests as prerequisites for comparing algorithms.

**Figure 5:**
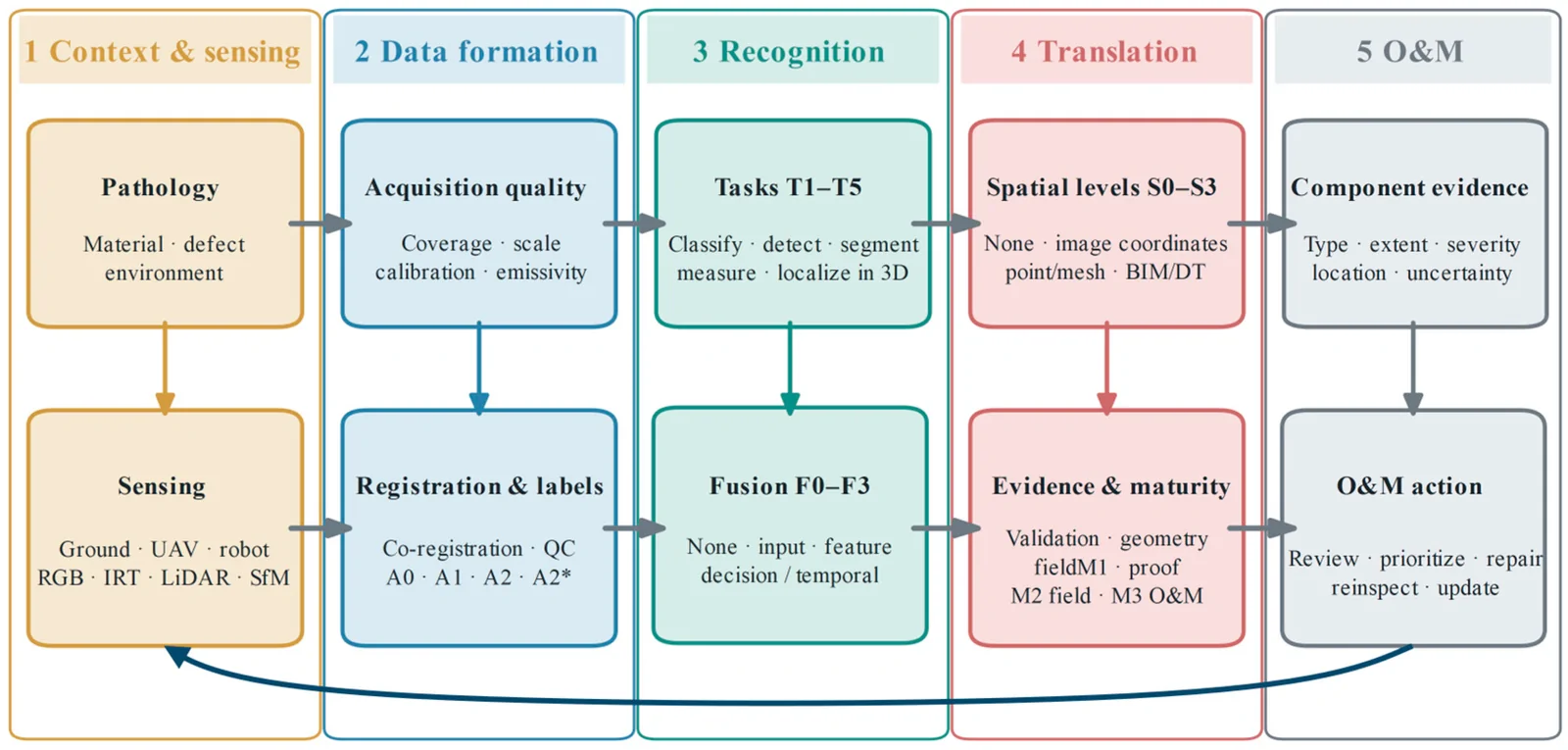
Pathology-aware technical pathway from sensing to engineering O&M.

Facade-defect recognition is treated here as an information pathway rather than an isolated prediction task (Figure 5). Material, defect mechanism, and environment determine what can be sensed; acquisition quality and registration determine what can be annotated; task and fusion levels determine the information retained by the model; and spatial mapping, validation, and maturity determine whether that information can support an O&M decision. The same pathway also structures the review: Section 3 examines data formation and labels, while Section 4 follows recognition outputs through fusion, measurement, 3D association, and engineering use.

### 3.1. Acquisition Platforms, Material Systems, and Imaging Conditions

Pose deviation, shooting distance, and illumination changes are often mutually coupled. When an acquisition platform avoids obstacles, sweeps across floors, or changes its observation angle, it simultaneously alters the geometric relationships among the camera, the facade, the light source, and the target defects, causing perspective distortion, scale variation, shadow displacement, enhanced reflections, and edge blurring. In their inspection of residential facades in Shenzhen, Fan et al. noted that low-rise buildings allow close-range acquisition at 5–10 m, whereas high-rise buildings usually require a distance of about 20–25 m to balance coverage efficiency, flight safety, and recognition capability [37]. Studies on UAV crack segmentation and multitemporal aerial oblique imagery also show that shadows, low contrast, texture interference, viewpoint differences, and cross-temporal pose inconsistency all weaken the recognition of cracks or facade damage [38,39].

**Figure 6:**
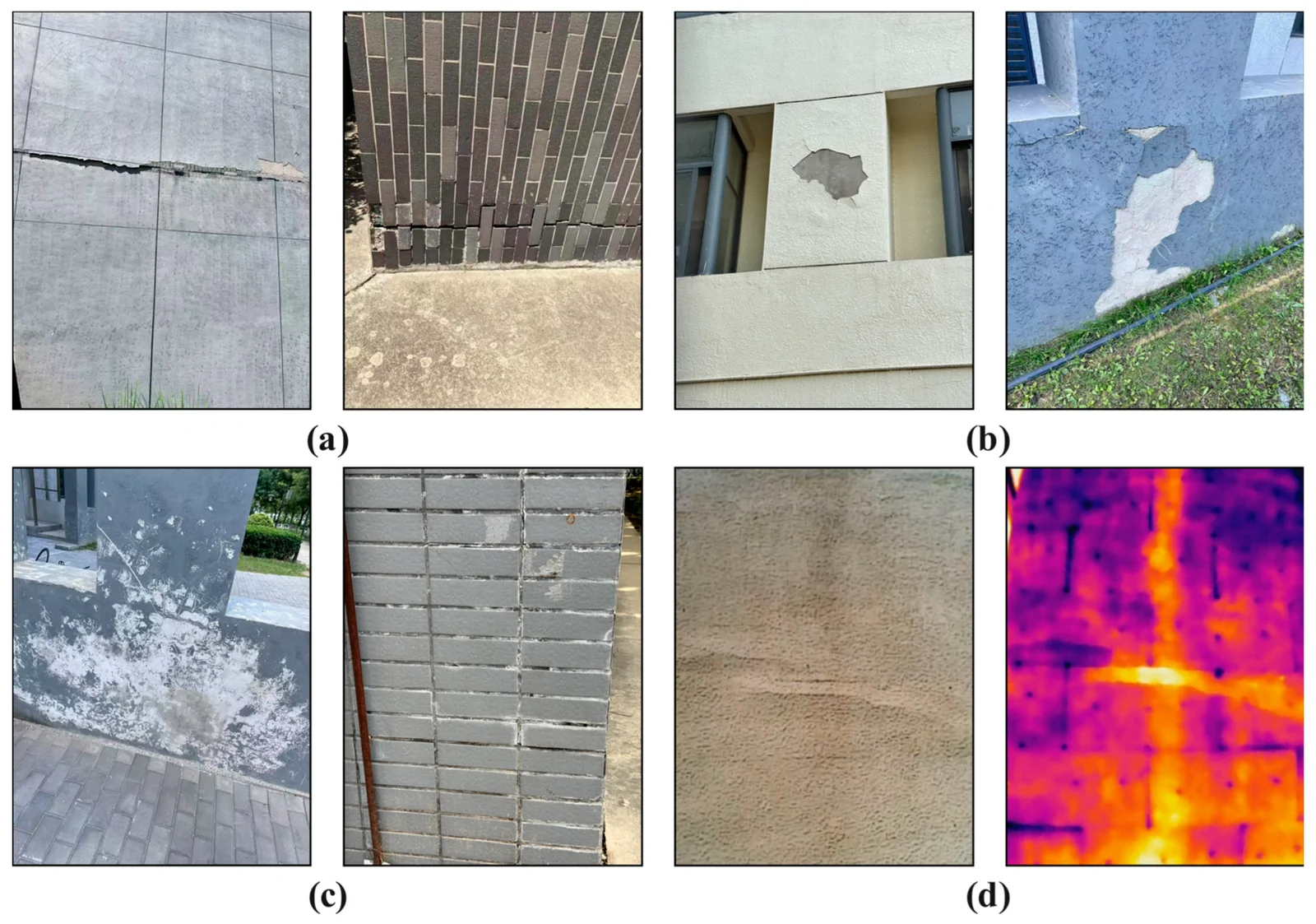
Representative field manifestations of facade defects and their material, component, and sensing contexts. (**a**) Visible crack with surrounding facade texture and component boundaries. (**b**) Finish or cladding detachment shown within its local component context. (**c**) Moisture-related seepage, efflorescence, or mold near a joint, opening, or drainage path. (**d**) Corresponding RGB and infrared thermographic views of a weakly visible anomaly. All visible-light photographs and infrared thermograms were acquired by the authors from buildings on the campus of Beijing University of Civil Engineering and Architecture, Beijing, China.

Infrared thermography provides a complementary channel for weakly visible defects, but its interpretation depends on heat conduction processes, material emissivity, ambient temperature differences, acquisition time, wind speed, and humidity. IRT images of high-rise facades are often accompanied by blurred boundaries, abundant interference, and class imbalance [30]. Studies on thermographic imaging of facade debonding also caution that saliency maps such as Grad-CAM can only indicate the regions a model attends to and cannot directly prove a causal relationship between thermal anomalies and actual debonding [40]. Research on thermographic detection of bridge-deck delamination further shows that environmentally induced non-uniform temperature backgrounds can lead to over- or under-estimation, requiring background correction or morphological reconstruction to separate physical anomalies from environmental noise [41]. IRT is therefore better suited to providing complementary evidence for delamination, debonding, seepage, and thermal anomalies, as illustrated by the corresponding RGB and thermal observations in Figure 6d.

Building facades feature regular components and repetitive textures. Balconies, air-conditioning units, drainpipes, railings, vegetation, billboards, brick joints, window frames, shadows, and drainage stains may all interfere with defect interpretation. Facade parsing research indicates that building facades are structured scenes with component organization and repetitive patterns [42,43,44]. These front-end constraints define the upstream boundaries for subsequent annotation, training, generalization, and multimodal fusion.

The facade material system also determines defect visibility, thermal response, and annotatable boundaries. When drainage, sealing, insulation, or material interfaces fail, cracks, seepage, delamination, debonding, efflorescence, mold, and thermal bridges manifest differently. Figure 6 presents representative field manifestations of these defects in their material, component, and sensing contexts. The visible-light examples retain surrounding joints, openings, finishes, and component boundaries, while the paired RGB and IRT views show how thermal evidence can supplement visual appearance when defect boundaries are weak or ambiguous.

Together, these examples show why defect labels cannot be determined from local image texture alone. Experience with rain control and EIFS/external insulation shows that facade moisture damage is closely related to joints, openings, drainage layers, flashing details, drying capacity, rainwater intrusion, and hygrothermal stress. Dataset construction should therefore record material type, construction layering, joint positions, exposure environment, and maintenance history alongside the images and annotations. Table 4 summarizes common facade materials, typical defect manifestations, and environmental triggers.

Figure 6 and Table 4 together show that facade defects cannot be treated as generic visual targets independently of their material and component contexts. Brick joints can resemble cracks, seepage boundaries are often gradual, and thermal bridges or seal failures in glass curtain walls require thermal and component-level evidence. Facade datasets should therefore record material, component, defect boundary, environmental condition, and sensor modality together.

### 3.2. Annotation Granularity and Task Definition

The main annotation challenge is the unstable correspondence among defect boundaries, component semantics, and repair implications, rather than the distinction among A0, A1, and A2 alone. Cracks may follow brick joints, window frames, or insulation-board joints; seepage and thermal anomalies often have gradual boundaries; and delamination or debonding may have little visible expression. Facade annotations must specify defect presence and location, whether the boundary can be delineated quantitatively, and whether the label can support a repair decision.

Image-level labels are suitable for low-cost screening but carry limited engineering semantics. SDNET2018 provides more than 56,000 crack/non-crack images covering interference conditions such as shadows, rough surfaces, holes, edges, and debris [26]; the multiclass wall-crack dataset of Wibowo et al. emphasizes that crack subtypes and decision boundaries require expert knowledge [45]. Bounding-box annotation improves localization capability but remains coarse for elongated cracks, localized seepage, and small-scale debonding. CODEBRIM, CUBIT-Det, and the UAV building-surface defect dataset extend multi-defect detection evidence from the perspectives of bridge concrete, cross-infrastructure scenarios, and building surfaces, respectively [27,46,47]. However, the background within bounding boxes often far exceeds the defect pixels, and models may learn co-occurring textures such as tile joints, shadows, decorative lines, or window-frame edges. Pixel-level masks come closest to repair-quantification needs; both masonry-wall segmentation and RGB-IRT facade damage datasets show that texture noise, modality differences, and instance-partition rules jointly determine final segmentation quality [31,48]. Facade datasets should therefore explicitly state annotator qualifications, category definitions, rules for handling ambiguous samples, and inter-annotator consistency.

Thermal and dual-modality data depend even more strongly on task definition. Thermal bridges, seepage, and debonding are usually not sharp geometric contours but anomalous regions jointly delimited by temperature differences, component locations, and expert interpretation. Cones et al. incorporated walls, windows, doors, and thermal bridges into thermogram instance segmentation [49], while Mirzabeigi et al. combined building-component semantics with thermal anomaly identification [50]. These studies indicate that IRT and RGB-IRT datasets should report defect categories, component categories, annotation rules, and public availability together.

### 3.3. Negative-Sample Composition and Cross-Scene Generalization

In facade scenarios, “negative samples” are by no means simply normal walls. Real inspections involve at least four sources of confusion: normal facades, non-defect textures, non-facade backgrounds, and non-target defects. Brick joints, expansion joints, drainage stains, shadows, soiling, decorative lines, window-frame edges, thermal bridges, and moss may all resemble real defects; if these heterogeneous samples are compressed into a single “background” class, the model’s decision boundary is forced to blur, ultimately manifesting as false and missed detections.

SDNET2018 and CODEBRIM explicitly include numerous interference conditions, providing adjacent evidence of negative-sample complexity. The former incorporates shadows, surface roughness, holes, edges, and debris into its range of image variation [26]; the latter handles cracks, spalling, exposed rebar, weathering, and seepage in multiclass concrete defects while introducing safety-irrelevant variations such as illumination, weather, stains, holes, markings, and graffiti [27]. This shows that facade datasets cannot contain only “clean cracks” and “clean normal walls.”

Cross-domain generalization is the external manifestation of negative-sample complexity in model evaluation. Facade images differ markedly in materials, climate, orientation, acquisition platform, shooting distance, sensor type, defect categories, and maintenance status. Katsigiannis et al. applied transfer learning to crack detection on masonry facades, showing that pre-trained features can improve single-dataset performance, but scene scope and data scale still limit extrapolation [51]. Thermal anomaly recognition likewise depends on target-domain physical conditions and anomaly definitions; the studies of Mirzabeigi et al. and Kurtser et al. show that component semantics, target-domain thresholds, training-data availability, and thermal-response differences all affect the scalability of building-envelope anomaly identification pipelines [50,52].

Cross-dataset experiments in adjacent fields further suggest that high accuracy under random splits of a single dataset is insufficient to demonstrate engineering deployability. Rashid et al. evaluated classification models on six public structural-surface crack datasets, finding high self-testing performance but marked cross-dataset degradation [53]. For facade inspection, a minimum generalization evaluation should include cross-building, cross-material, cross-distance, cross-sensor, and cross-city testing. Studies on tile debonding and aluminum–plastic composite panels also show that different materials have different failure mechanisms, visible defect types, and detection thresholds [54,55]. Cross-material generalization is therefore not merely an image-texture transfer problem; it also involves the coupling of defect-generation mechanisms, observable physical responses, and engineering semantics.

### 3.4. Dataset Modalities, Annotation Bias, and Evaluation Differences

Existing datasets and related studies can be grouped into RGB visible-light data, infrared thermography, registered RGB-IRT pairs, street-view or oblique imagery, 3D geometric mapping data, and aggregated general-crack data. RGB imagery is inexpensive, intuitive to annotate, and supported by a mature model ecosystem, making it suitable for detecting cracks, spalling, seepage stains, and surface damage; however, it is sensitive to illumination, shadows, reflections, soiling, and surface texture [37,47,51]. Infrared thermography is used mainly to detect delamination, debonding, thermal bridges, seepage, and thermal anomalies. Its limitations include diffuse boundaries, small sample sizes, emissivity differences, environmental thermal backgrounds, and limited interpretability [30,40,49,50,52]. Registered RGB-IRT data can combine visible texture with thermal response. Wang et al.’s multimodal facade-damage dataset contains 1761 image pairs with reported registration errors [31], while Kurtser et al.’s color-to-thermal anomaly data use visible images to predict thermal responses under sparse anomaly annotation [52]. Such studies should also report registration error, acquisition time, thermal conditions, and failure cases.

Street-view, oblique photogrammetry, and 3D-augmented data provide transitional evidence from 2D recognition toward city-scale assessment and spatial localization. Street-view facade data can support the identification of building storeys, types, materials, and image usability [56]; multitemporal aerial oblique imagery highlights viewpoint changes and spatial registration errors [39]. Kalfarisi et al. mapped crack segmentation onto 3D reality meshes for length, width, and visual quantification [57]; PointCrack3D provides 21 colored LiDAR scans covering more than 900 m^2^ with point-level annotations of 412 cracks [58]. Both demonstrate that spatial anchoring is feasible but are not yet sufficient to constitute an open 3D multi-defect benchmark for facades.

Road and general crack datasets retain methodological transfer value. CrackTree, random structured forests, FPHBN, DeepCrack, and CrackSeg9k cover traditional image processing, random forests, feature pyramids, hierarchical feature learning, and cross-dataset aggregation [59,60,61,62,63]. They help characterize thin linear structures, class imbalance, and segmentation-evaluation protocols. However, their background textures, acquisition poses, defect semantics, and repair logic differ from those of facades, so their results should not be treated as evidence of facade engineering performance.

Table 5 summarizes representative datasets and evidence sources. It retains core facade, wall, and building-envelope data together with selected bridge, general-crack, cross-structure, and point-cloud entries that illustrate transferability or modality coverage rather than facade-specific performance. Existing data remain concentrated on RGB crack images and a small number of multi-defect tasks. IRT and RGB-IRT provide additional evidence for weakly visible defects, but public availability, annotation consistency, and cross-domain replication remain limited. LiDAR and 3D data are used mainly for crack localization, point-cloud mapping, or 3D visualization; open point- or mesh-level benchmarks for multiple facade defects remain scarce. Future datasets should jointly record material, component, defect, modality, and spatial position, together with acquisition distance, viewing angle, floor level, sensor parameters, and maintenance history [47,56,64,65].

**Figure 7:**
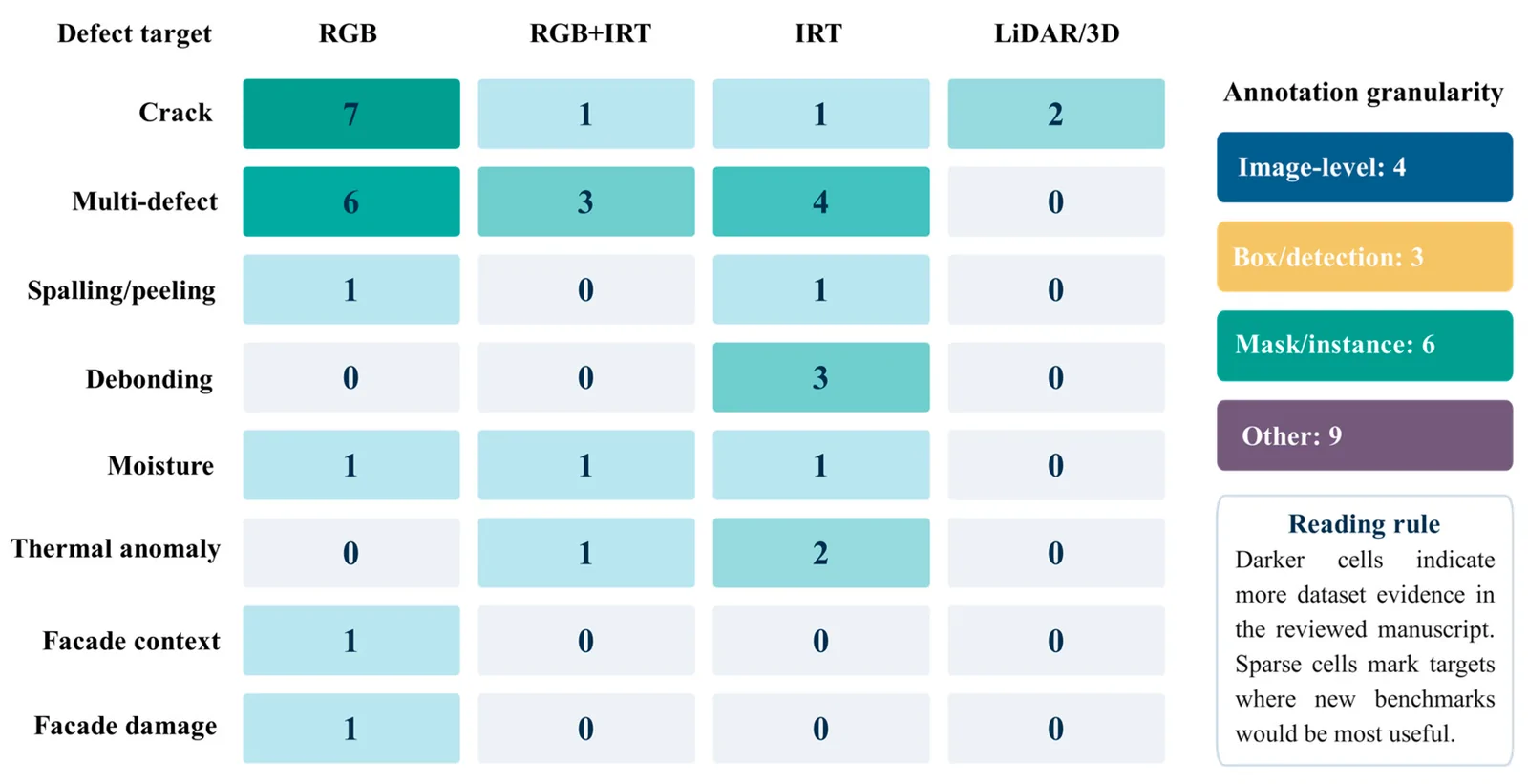
Coverage of different data evidence sources.

A comparison of Table 4 and Table 5 explains the uneven coverage shown in Figure 7: RGB datasets concentrate on cracks and multi-defect tasks because cracks, spalling, and surface damage more readily form visible textures; IRT and RGB-IRT cover more thermal anomalies, seepage, and debonding, stemming from hygrothermal transport, interface voids, emissivity differences, and thermal-bridge effects; LiDAR/3D is better suited to crack localization, panel deformation, component misalignment, and 3D mapping. The data challenge is not simply insufficient sample size, but insufficient joint annotation of modalities, materials, component semantics, and spatial localization.

The central data problem is not sample count alone, but the absence of common protocols for acquisition conditions, annotation granularity, negative-sample design, sensor modality, and engineering semantics. High accuracy on a random split of one dataset does not establish reliability across buildings, materials, cities, or sensors. The analyses of single-modality models, multimodal fusion, 3D mapping, and interpretability that follow should therefore be read against these sensing and generalization constraints.

## 4. Multi-Level Visual Analysis Framework and Engineering Applicability

Single-modality vision remains the most mature and accessible basis for automated facade inspection. At F0, it covers T1 classification, T2 detection, T3 segmentation, and T4 geometric quantification. Systems that incorporate pose information, stereo measurement, or coordinate recording may extend to T5 and S2/S3. Their practical limits remain tied to the acquisition, material, and annotation conditions described in Section 3. The following subsections assess single-modality methods by task level and deployment requirements, then examine multimodal fusion, 3D localization, and supporting tools for trustworthy use. Three information products recur throughout this discussion: visual defect evidence, calibrated geometric or dynamic measurements, and structural-state inference. These outputs are not interchangeable. The latter two may inform facade O&M, but high image-level accuracy alone does not establish either; scale calibration, physical validation, and component context are also required.

### 4.1. Image Classification and Object Detection: Coarse Screening and Pre-Localization at the T1–T2 Levels

Image-level classification corresponds to T1 and A0 and is suitable for initial large-scale screening. Early CNNs, modified AlexNet variants, pretrained networks, and analyses of noisy samples have shown that deep features can screen for cracks and recognize defects on multiclass structural surfaces containing shadows, stains, holes, and other interference [25,66,67,68]. Transfer learning has reduced the amount of task-specific data required by adapting ImageNet-pretrained networks to crack recognition on pavements, concrete, building surfaces, and masonry facades [51,69,70,71,72]. However, T1 classification mainly answers whether further inspection is needed and cannot indicate defect location, component affiliation, length, area, or delamination extent. Chen et al. reported that a plain CNN achieved a test accuracy of about 88.92%, whereas transfer learning increased it to about 94.34%, with precision, recall, and F1-score of approximately 0.95 [72]. This result indicates that transfer learning can improve small-sample screening, but image-level outputs remain insufficient for repair decisions. Recent studies of facade-defect ensembles, exterior-surface defect detection, and UAV-based building maintenance likewise indicate that T1/T2 models can support initial screening but do not delineate defect boundaries or repair semantics [73,74,75,76].

Object detection advances the task to T2, typically corresponding to A1 bounding boxes and S1 image-coordinate localization. Faster R-CNN and the YOLO family provide general frameworks for proposal generation, classification and regression, and real-time localization [77,78,79,80], while region-based convolutional networks have been applied to multiclass structural-damage detection [81,82,83]. For facade applications, the more important question is whether a model is adapted to facade defects and field workflows. As shown in Table 6, BFD-YOLO reported a precision of 81.6%, recall of 77.8%, mAP@0.5 of 82.4%, F1 of 79.7%, and 76 FPS for multiclass building-facade defect detection [28]. CladdingNet integrated UAV acquisition, binocular vision, embedded GPUs, and 3D coordinate recording for crack detection on stone curtain walls, reporting PA 0.99, mPA 0.86, mIoU 0.86, F1 0.91, 1–10 FPS, a crack-measurement precision of 1.0 mm, and an error rate below 10% [84]. Zhou and Tiong combined detection results with report generation [29]. These studies indicate that T2 detection is beginning to enter field workflows, although outputs remain predominantly at the 2D box level. Machine-learning detection for stone and brick masonry, YOLO-DL multitask crack recognition and measurement, DAH-YOLO, and metaheuristic-optimized crack detection further indicate that models are increasingly designed to balance complex backgrounds, real-time performance, and preliminary quantification [48,85,86,87,88,89,90].

Classification and detection retain a clear information bottleneck. Image-level labels have no spatial interpretation, while bounding boxes approximate the boundaries of cracks, seepage, and debonding only coarsely. For elongated cracks, background pixels may greatly outnumber defect pixels, encouraging models to exploit brick joints, shadows, window frames, or decorative lines. The negative-sample complexity discussed in Section 3.3 therefore appears directly as false positives at T1–T2; adjacent studies also show that class balance affects crack-recognition stability [91]. T1–T2 methods are best used for screening and pre-localization. Pixel-level segmentation is still required before area, length, width, or 3D position can be estimated.

### 4.2. Pixel-Level Segmentation and Defect Quantification: Geometric Representation at the T3–T4 Levels

Semantic segmentation advances defect recognition from box-level localization to pixel-level contours, corresponding to T3 and A2, and forms the basis for facade defect quantification, 3D mapping, and repair assessment. FCN, U-Net, SegNet, the DeepLab family, and Mask R-CNN constitute a segmentation toolchain from the perspectives of dense prediction, encoder–decoder design, upsampling efficiency, multi-scale context, and instance masks, respectively [92,93,94,95,96,97]. These architectures provide pixel-level inputs for cracks, spalling, seepage, and local damage, and lay the groundwork for subsequent S2/S3 spatial mapping.

Crack segmentation is harder than general semantic segmentation because cracks are elongated, occupy few pixels, and are easily confused with shadows, brick joints, construction joints, and material texture. Yang et al. combined pixel-level crack detection with width measurement, linking T3 segmentation to T4 geometric quantification; the reported measurement errors also show the effects of image resolution, boundary blur, and post-processing [98]. DeepCrack, CrackNet, CrackU-Net, MSMC-U-Net, and Gated Scale Pooling address crack continuity and boundary integrity through multiscale features, deep supervision, pooling-free designs, recurrent modeling, and context fusion [62,91,99,100,101,102,103,104,105,106,107]. Although most evaluations concern roads, bridges, or concrete, their treatment of thin structures, class imbalance, and scale remains relevant to facades. Deep models generally outperform edge operators, but texture interference, threshold selection, and negative-sample design still require explicit control [108].

Facade applications are moving from crack recognition toward condition assessment. Chen et al. used CNN-based filtering before U-Net segmentation to reduce background redundancy and class imbalance in close-range facade images [109]. Xu et al. combined DeepLabv3+ with connected-component analysis and the analytic hierarchy process to extract crack geometry and assign damage grades, extending the workflow from T3 to T4 [110]. Wang and Wu developed a multiscale feature-fusion network for tile spalling; despite the clearer regional appearance of spalling, available data and cross-material validation remain limited [111]. YOLO-DL, integrated detection–segmentation–quantification pipelines, self-supervised crack detection, and Co-CrackSegment further connect masks with geometric measurements [86,87,112,113].

Instance segmentation can output mutually separated cracks, spalling regions, or seepage stains as independent objects, making it better suited to defect counting, area ranking, and repair prioritization. For facade maintenance management, several small spalling spots and one continuous large spalling area imply different risks, giving instance segmentation frameworks such as Mask R-CNN engineering significance [97]. However, instance segmentation depends on clear object boundaries and instance partition rules; without unified annotation standards in the dataset, the “instance counts” output by a model may not carry stable repair meaning.

#### From Static Defect Geometry to Video-Based Structural Measurement

Static facade-defect quantification and dynamic structural measurement share a need for scale, calibration, uncertainty control, and external reference data, but they address different physical quantities. Crack width, spalling area, or delamination extent are derived from defect boundaries, whereas video-based SHM estimates displacement, tilt, vibration, or modal response from temporal image motion. A typical quantitative vision pipeline comprises camera calibration, target or feature tracking, and transformation from pixel motion to structural coordinates [19]. Stereo-photogrammetry and phase-based motion processing can extend this process to full-field or three-dimensional operating shapes, although synchronization, camera geometry, resolution, and image noise constrain the recovered motion [20].

Robust field use also requires algorithms that remain stable across motion scales and imaging conditions. Integrated video frameworks combining phase-based processing and template matching allow the measurement method to be selected according to the structural response and camera configuration [21], while learned fusion of independently extracted video signals can improve the signal-to-noise ratio of displacement estimates [22]. For facade inspection, these developments are most relevant to component deformation, curtain-wall movement, vibration, and the validation of image-derived geometry. They do not convert a surface mask directly into structural damage; calibration uncertainty, physical interpretation, and comparison with reference measurements remain necessary.

Complementary nonvisual SHM studies reinforce this boundary. FBG monitoring combined with SSI-COV converts measured strain responses into validated modal parameters and damage indices [23], while modal-identification reviews emphasize environmental variability, stability diagrams, and parameter validation before damage inference [24]. Their relevance to facade vision is methodological: recognition outputs must likewise be transformed into calibrated, component-linked, and uncertainty-bounded engineering variables before they are used in O&M decisions.

### 4.3. Advanced Visual Architectures and Edge Deployment

Transformers and hybrid architectures introduce global-dependency modeling into single-modality vision. ViT, Swin, SegFormer, DETR, and TransUNet support image recognition, high-resolution hierarchical modeling, lightweight segmentation, set prediction, and local–global fusion, respectively [114,115,116,117,118]. In crack tasks, CA-SegFormer and multitemporal Swin UNETR show that global modeling helps maintain the continuity of long cracks and temporal consistency [119,120]. For facades, however, long-range dependency solves only part of the problem. Microcrack tips, high-frequency boundaries, and repetitive textures still depend heavily on local detail, so pure transformer models are not necessarily superior to CNNs or hybrid architectures.

Vision foundation models such as SAM raise the ceiling of single-modality segmentation. SAM possesses general segmentation capability [121]; CrackSAM fine-tuned SAM with adapters and low-rank adaptation and constructed a dataset of 810 road and facade crack images to examine zero-shot and cross-scene segmentation capability [122]. Studies on semi-supervised SAM crack detection, Segment Any Crack, and combined SAM–U-Net evaluations also show that, after parameter-efficient fine-tuning or task adaptation, SAM can improve crack segmentation under low light, shadows, and complex textures [123,124,125,126]. Nevertheless, in automated facade inspection SAM still faces prompt dependence, uncertain material generalization, and excessive parameter counts, making it more suitable as an annotation aid, a semi-automatic segmentation tool, or an offline analysis tool.

Mamba/SSM methods attempt to balance long-range dependency and computational efficiency. SCSegamba achieved an F1-score of 0.8390 and mIoU of 0.8479 on multiscene crack datasets with only 2.8 M parameters [33]; HFR-Mamba reported IoU of 79.34% on CrackTree200 and mIoU of 71.27% on GAPS384, with 22.1 M parameters and 21.9 G FLOPs [127]. Results on the synergy of Visual Mamba with convolutional networks and on Mamba-Enhanced HRNet also suggest that SSMs are more likely to complement the local-detail branches of CNN/HRNet than to fully replace convolutional priors [128,129]. Existing evaluations, however, still come mainly from road, bridge, concrete, and general crack data, lacking validation across facade materials, defect types, floor levels, and platforms.

Field-inspection models must combine accuracy with executable performance under limited computing power, battery capacity, and high-resolution input. Desktop GPUs differ substantially from UAVs, mobile phones, wall-climbing robots, and portable edge devices. FLOPs and parameter counts do not translate directly into measured FPS, because input resolution, inference framework, batch size, quantization, operator support, and data transfer also affect latency. INT8 or FP16 quantization may further break fine cracks, shrink boundaries, or suppress small defects.

Facade scenarios amplify the accuracy–efficiency conflict. High-rise facades require high resolution to keep micro-cracks visible, which increases memory usage and latency; lowering resolution loses fine-grained defects, while tiling introduces repeated inference and stitching costs. In their inspection of residential facades in Shenzhen, Fan et al. reported crack segmentation with aAcc 98.03%, mIoU 87.86%, and Dice 93.23%, and thermal-assisted seepage detection with aAcc 98.41% and mIoU 79.05% [37], noting that acquisition distances of 5–10 m for low-rise and about 20–25 m for high-rise buildings must balance accuracy, efficiency, and safety.

Edge deployment is not mere model compression but a redefinition of the task. Initial screening can use lightweight classifiers or detectors; repair-quantity estimation must retain segmentation and geometric quantification; and 3D mapping additionally requires pose, scale, and component semantics. Future single-modality research should jointly report recognition, deployment, and engineering metrics, including Acc, P/R/F1, mAP, mIoU, Dice, latency, FPS, parameter count, FLOPs, input resolution, per-unit facade processing time, minimum detectable crack width, cross-building generalization, and failure cases.

Table 6 shows that single-modality methods now range from CNN classifiers and detectors to segmentation networks, Transformers, SAM-based models, and Mamba architectures. Facade-specific constraints—material diversity, repetitive component patterns, difficult viewpoints, and limited edge resources—remain underrepresented in validation. Classification and detection support screening, segmentation supports geometric quantification, and newer architectures may improve contextual modeling or efficiency. A complete O&M workflow, however, still requires spatial mapping, multimodal evidence where appropriate, and independent verification.

**Table 6:** Overview of representative single-modality visual methods.

Category/Studies	Input and Task	T/F/S/M	Key Reported Evidence	Engineering Use and Limitation
CNN classification: Cha et al. [25]; Dorafshan et al. [108]	RGB/grayscale cracks	T1/F0/S0/M1	Cha: train/validation Acc 98.22/97.95%	Fast screening; no location, boundary, or size
Building-surface classification: Chen et al. [72]	RGB cracks	T1/F0/S0/M1	CNN Acc 88.92%; transfer learning Acc 94.34%, P/R/F1 ≈ 0.95	Useful with small samples; insufficient for repair quantities
Detection: Faster R-CNN [77], YOLO [78,79,80], BFD-YOLO [28], DAH-YOLO [88]	RGB multiclass defects	T2/F0/S1/M1–M2	BFD-YOLO P/R/mAP50/F1 81.6/77.8/82.4/79.7%, 76 FPS, 26.8 M; DAH-YOLO P 83.0–90.2%, R 71.6–84.9%, mAP50 gain 5.2–8.4%	Pre-localization and reporting; box redundancy for thin cracks
UAV facade systems: CladdingNet [84], Zhou and Tiong [29], Interlando et al. [73], Chen and Li [74], Kung et al. [75]	UAV RGB; detection and partial 3D positioning	T2–T5/F0/S1–S2/M2–M3	CladdingNet PA/mPA/mIoU/F1 0.99/0.86/0.86/0.91, 1–10 FPS, error < 10%; other reported Acc 84.42–90.9%, class P/R 65.29–91.57/77.55–98.00%, mAP50 82.56%	Closest to field workflows; platform, range, and resolution dependent
General segmentation: FCN [92], U-Net [93], SegNet [94], DeepLab [95,96]	RGB cracks/damage	T3/F0/S1/M1–M2	FCN VOC2012 mIoU 62.2%; SegNet CamVid road Acc 88.6%; DeepLabv3+ VOC2012 mIoU 89.0%	Geometric contours; costly labels and domain shift
Facade segmentation/quantification: Chen et al. [109], Xu et al. [110], Wang and Wu [111], YOLO-DL [86], Co-CrackSegment [113]	Facade cracks/tile spalling	T3–T4/F0/S1/M2	Chen F1 crack/non-crack ≈ 94/96%; Xu mIoU 76.40%, mAP 87.47%, F1 0.80, Acc 99.36%; YOLO-DL P 84.87%, mAP50 83.55%, mIoU 94.94%; IoU/P/R/Dice/mAP = 72.98/82.22/86.00/83.62/85.80%	Supports quantity estimation; limited cross-material data
UAV residential facades: Fan et al. [37]	RGB + IRT; cracks/seepage	T3–T5/F0–F1/S1–S2/M2	Cracks aAcc/mIoU/Dice 98.03/87.86/93.23%; seepage aAcc/mIoU/P/R 98.41/79.05/87.54/86.44%	Facade-specific; affected by distance, thermal context, and registration
Crack segmentation: DeepCrack [62,99], CrackNet [100,101], MSMC-U-Net [104]	Road/bridge/concrete cracks	T3/F0/S1/M1	DeepCrack P/R/F1 0.8683/0.8637/0.865, mean IoU 86.5%, ~0.1 s/image at 544 × 384	Strong thin-line modeling; facade transfer unverified
Transformer/hybrid: ViT [114], Swin [115], SegFormer [116], TransUNet [118], CA-SegFormer [119]	RGB structural surfaces	T2–T3/F0/S1/M1–M2	ViT-B/16 ImageNet-1k Acc 77.9%; Swin-T 81.3%; SegFormer-B5 ADE20K/Cityscapes mIoU 51.0/84.0%	Long-range context; demanding for high-resolution edge use
SAM/foundation models: SAM [121], CrackSAM [122], semi-supervised methods [123,124,125,126]	RGB cracks/defects	T3/F0/S1/M1–M2	CrackSAM_adapter IoU 64.95%, Facade390 47.18%; SAC F1 61.22%, IoU 44.13%	Annotation and offline segmentation; prompt and compute dependence
Mamba/SSM: SCSegamba [33], MambaCrackNet [128], HFR-Mamba [127], MambaHRNet [123]	RGB cracks	T3/F0/S1/M1–M2	SCSegamba 2.8 M, F1 0.8390, mIoU 0.8479; HFR-Mamba IoU 79.34%, DSC 81.02%, 22.1 M/21.9 G; MambaHRNet Mean IoU = 0.963	Efficient long-range modeling; facade/material validation limited

### 4.4. Multimodal Data Alignment and Fusion Strategies

Multimodal facade inspection should begin with a physical question: does each sensor contribute information that the others cannot provide reliably? RGB images describe texture, color, and component context; infrared thermography captures thermal responses associated with delamination, debonding, seepage, and thermal bridges; and LiDAR or depth data provide geometry for deformation and component localization. The choice among F1 image-level, F2 feature-level, and F3 decision-level fusion should follow the defect mechanism, registration quality, task level, thermophysical conditions, and deployment cost.

#### 4.4.1. Spatial Registration and Modality Correspondence

The primary problem of multimodal detection is spatial alignment. RGB, IRT, and point clouds are inconsistent in resolution, field of view, distortion, and acquisition time; if pixels, components, or 3D surfaces cannot be reliably matched, a fusion model may learn registration bias rather than complementary features.

Wang et al. provide a useful benchmark for RGB-IRT facade segmentation. Their dataset contains 1761 infrared-visible image pairs. A principal-orientation feature-point method produced a reported registration RMSE of 14.35 px, after which registration, image fusion, and instance segmentation achieved a best mAP of 85.4% across four facade-damage classes [31]. The pipeline establishes technical feasibility, but a displacement of this magnitude is consequential for narrow cracks, seepage boundaries, and localized debonding because it can create duplicate edges and misplaced thermal responses.

The weakly aligned cross-modal learning of He et al. and their subsequent hierarchical bilateral feature-fusion network show that, when RGB-IR image pairs acquired by UAVs in the field contain translation errors, strict pixel-level registration need not be the sole prerequisite; modality associations can instead be established in feature space, distinguishing infrared-specific delamination/debonding responses from surface damage or spalling responses shared by both modalities [32,130]. Such studies indicate that, under consumer-grade UAV or mobile acquisition conditions, F2 feature-level tolerance to weak alignment is often more practicable in engineering than unstable pixel-level strict registration.

Registration errors affect different fusion levels differently. F1 image-level fusion is the most sensitive to pixel misalignment; F2 feature-level fusion can partially absorb translation, scale, and local deformation errors in the semantic space; and F3 decision-level fusion mainly manifests as inconsistency in the final spatial results. The fusion level should therefore be determined jointly by registration accuracy, defect scale, and task requirements, rather than chosen merely for architectural convenience.

#### 4.4.2. Fusion Levels and Task Adaptation

F1 image-level fusion merges modalities directly; its advantage is retaining low-level physical signals, suiting scenarios with high registration accuracy and strong physical fidelity requirements. General infrared–visible fusion methods such as DenseFuse, FusionGAN, IFCNN, U2Fusion, NestFuse, RFN-Nest, and SwinFusion provide the methodological basis for RGB-IRT facade fusion [131,132,133,134,135,136,137]; DeepFuse, Bayesian fusion, SeAFusion, and PAIF have further pushed fusion from visual-quality orientation toward task-performance orientation [138,139,140,141]. However, facade thermal anomalies are affected by solar radiation, emissivity, wind speed, humidity, and acquisition time; F1 evaluation cannot rely on SSIM or information entropy alone and should also report registration RMSE, thermal environment, and downstream mAP/mIoU [31,41]. RGB-X/RGB-T segmentation research offers transferable structures for facade feature interaction and real-time inference [142,143,144,145].

F2 feature-level fusion performs cross-modal interaction in intermediate network layers and is currently the more practicable route in multimodal facade detection. It does not require strict pixel coincidence yet can learn when to rely on RGB and when on IRT. WCL, graph-learning methods, and LBF2-Net all incorporate weak alignment, modality complementarity, and consistency into their designs [32,130,146]. Comparisons of RGB-T fusion levels for facades also show that no single optimal level exists independent of defect type and registration conditions [147]. For thermal-anomaly defects, F2 should be combined with environmental metadata and modality confidence rather than treating IRT features as unconditional enhancement. Infrared energy-related recognition of building envelopes, cross-modal detection of curtain-wall frames, and UAV multispectral debonding detection further show that the value of F2/F3 fusion lies in jointly constraining thermal anomalies, visible textures, and component semantics onto interpretable defect objects [148,149,150].

F3 decision-level fusion merges results after independent inference per modality, offering flexible deployment, module decoupling, and easy maintenance. RGB, IRT, and point-cloud models can be trained, replaced, and upgraded separately, suiting scenarios with changing sensor models, inter-organizational collaboration, or edge–cloud coordination. Yet moving multimodal systems toward engineering deployment incurs new costs: dual/triple-modality acquisition requires time synchronization and extrinsic-parameter maintenance; dual- or multi-branch networks increase parameters, memory, and power consumption; and high-resolution RGB, thermograms, and point clouds add transmission bandwidth and edge-caching pressure. Edge-intelligence research has pointed out that whether inference can be completed on the edge depends on computing power, bandwidth, latency, and task partitioning [151]; the validation of real-time RGB-T segmentation at 27 FPS on a Jetson Orin NX likewise shows that multimodal deployment must report hardware and efficiency [152]. Consequently, the value of F3 lies not merely in producing averaged results but in providing modality-consistency checks, conflict-region alerts, and manual-review prioritization. Future research should explicitly report RGB-IRT decision divergence rates, the area proportion of conflict regions, manual review ratios, modality synchronization strategies, and edge latency.

#### 4.4.3. Defect Types, Modality Selection, and Representative Evaluation Evidence

Whether multimodality is effective depends on the complementarity of physical responses. Cracks, spalling, and finish detachment are usually dominated by RGB textures and boundaries; delamination, debonding, seepage, and thermal bridges are more sensitive to IRT but require suitable thermal excitation, emissivity, and ambient temperature differences; spalling depth, deformation, and component misalignment depend more on LiDAR/depth data; and rebar corrosion, deep cracks, or internal voids may require NDT such as ultrasonics or GPR. Multimodal fusion should determine dominant and auxiliary modalities along the chain of “physical conditions–modality selection–fusion level–evaluation evidence.” Table 7 summarizes the relationship between typical defects and sensor responses.

Table 7 shows that fusion strategies should serve defect physical mechanisms rather than modality counts. When RGB can already express cracks or spalling clearly, IRT may only add thermal noise or registration error; thermal-anomaly defects require RGB for component localization and pseudo-anomaly exclusion; and component-level localization or volume estimation further requires point clouds, BIM, or other 3D data.

Existing research covers F1 image-level, F2 feature-level, and F3 decision-level fusion, but evaluation metrics, dataset sizes, registration conditions, and application targets differ substantially, making direct numerical ranking is inappropriate. To avoid misusing results obtained under different tasks and data conditions as a unified leaderboard, Table 8 therefore reports only representative metrics that can be verified in the original publications and interprets their engineering applicability using the T/F/S/M codes, rather than treating heterogeneous results as a common leaderboard.

Table 8 groups multimodal studies by registration and fusion strategy, thermal-anomaly detection, weakly aligned learning, and lightweight cross-modal modeling. Wang et al. [31] reported a registration RMSE of 14.35 px and a best mAP of 85.4% on 1761 RGB-IRT pairs; Wang et al. [153] reported an instance-segmentation mAP of 86.5% on 1228 infrared-visible pairs. These results support an end-to-end registration–fusion–segmentation workflow, although registration remains important for narrow boundaries. He et al. [32,130] reported a pixel accuracy of 98.84%, IoU of 0.7145, and F1 of about 0.78; LBF2-Net achieved an IoU of 0.8115 and F1 of 0.7819. These F2 methods are better suited to weakly aligned UAV data. Elamin et al. [156] showed that LiDAR does not guarantee improvement; gains depend on modality quality, target scale, and defect type. DMM and MambaRefine-YOLO [158,159] offer efficient RGB-IR interaction but still require facade-specific validation, while heat-leakage mapping, debonding diagnosis, and UAV-IRT detection provide additional F1–F3 evidence [154,155,157].

Multimodal performance is governed by three linked choices: the defect mechanism determines the dominant sensor, the fusion level determines how information is combined, and deployment conditions determine whether the system is practical. Registration error, environmental conditions, recognition performance, modality disagreement, and edge-computing cost should therefore be reported together.

### 4.5. Three-Dimensional Engineering Localization of 2D Recognition Results

If 2D detection and segmentation remain at the S1 image-coordinate level, they can only indicate the position and extent of defects within images and cannot yet support component localization, area accounting, repair prioritization, and long-term updating. Only when cracks, spalling, delamination, or seepage are mapped into 3D space and associated with component IDs, geometric dimensions, and repair attributes do recognition results advance from T3 to T4/T5 and extend to S2 point clouds/meshes or S3 BIM/digital twins.

#### 4.5.1. Three-Dimensional Geometric Foundations

The geometric basis for this mapping can be described using four complementary data models: point clouds, wireframes, surface models, and solid models. Point clouds provide discrete measurements; wireframes represent boundaries and topology; surface models provide continuous geometry and texture; and solid models add component semantics. These representations are not mutually exclusive and support different parts of the T4/T5 and S2/S3 workflow.

Point clouds represent building surfaces as discrete 3D samples generated mainly by LiDAR or SFM/MVS. In contrast, 3D Gaussian Splatting uses optimized Gaussian primitives to represent reconstructed scenes for view synthesis and visualization; it is not a point-cloud acquisition source [160]. LiDAR is less dependent on texture and illumination, whereas SFM/MVS is lower-cost and readily integrated with UAV inspection. Maru et al. segmented facade components based on LiDAR point clouds and RandLA-Net [161], and Chen et al. used 3D laser scanning and an improved k-d tree to assess facade flatness and verticality [162]. CAD object recognition, TLS facade feature extraction, and PCL also provide foundations for point cloud–BIM registration and geometric analysis [163,164,165]. Yet millimeter-scale fine cracks often still require high-resolution texture or 2D segmentation to retain semantics.

Wireframe models express boundaries through line segments, contours, skeletons, and isolines, suiting the recording of window frames, panel joints, component outlines, crack centerlines, and facade grids. With small data volume and clear topology, they can serve as an intermediate representation between 2D results and BIM components, but they can hardly carry continuous textures, areal seepage, spalling area, or thermal-anomaly distributions.

Surface models express continuous facades through triangular meshes, textured meshes, oblique-photogrammetry reality models, or orthographically unfolded surfaces, suiting the projection of 2D detection boxes or masks and the computation of crack length, spalling area, and spatial position. Pantoja-Rosero et al. realized automatic representation of damage in 3D models [35], Kalfarisi et al. combined crack detection with 3D reality meshes for quantitative assessment [57], and Jiang et al. used photogrammetric point-cloud features for defect detection and visualization [166]. Their limitations stem mainly from occlusion, glass reflection, weak texture, and illumination differences.

Solid models represent walls, windows, columns, curtain-wall units, storeys, and material attributes as BIM/IFC or digital-twin objects. They allow defect results to be linked to component IDs, repair attributes, and status updates. Zhang et al. registered UAV images to BIM [167], while Yang et al. mapped detected defects into BIM project coordinates [34]. The value of a solid model is therefore not higher pixel-level accuracy, but conversion of a defect in an image into a record attached to a specific building component. In summary, point clouds support measurement, wireframes support topology, surface models support projection and area calculation, and solid models support component semantics and O&M updating.

#### 4.5.2. Coordinate Unification and Mapping of 2D Results

The key to bringing 2D results into 3D space is unifying image, point-cloud, BIM, and sensor coordinates within a common reference frame. This involves camera intrinsics and extrinsics, pose estimation, point-cloud/BIM registration, and result back-projection; without unified coordinate systems, detections that are accurate in the image will still deviate from the true component positions.

Laguela et al. automatically mapped thermograms and RGB textures onto as-built BIM, providing an early case of thermography–visible–BIM registration [168]. The UAV image-to-BIM registration method of Zhang et al. localizes facade defects through geometric matching between BIM views and UAV images [167]. The AI–GeoBIM fusion framework proposed by Zhang et al. achieves 3D global localization and semantic registration of defects through virtual building models, camera geometry, and geographic metadata [169]. These studies show that 2D-to-3D mapping relies on the joint constraints of camera models, coordinate transformations, BIM semantics, and field metadata.

Mapping 2D masks or detection boxes onto 3D surfaces usually involves camera calibration, model reconstruction, point-cloud/BIM registration, and ray intersection. Errors at any stage affect defect position, area, and component affiliation. Grazing angles on facades are especially prone to amplifying pixel-boundary errors, most notably near balconies, window openings, corners, and edges. Photogrammetric uncertainty studies also point out that SFM/BA and MVS errors propagate into point-cloud coordinates and surface geometry [170]. Therefore, when T3 advances to T4/T5, metrics such as reprojection error, registration error, 3D RMSE, component matching accuracy, and length/width/area error should be reported alongside—rather than only 2D mIoU or mAP. Table 9 summarizes the main error chain.

Tan et al. proposed a mapping framework from UAV-image defect data to BIM, attaching defects as BIM family objects with attributes and locating them on model surfaces through coordinate transformation, deep-learning detection, and Dynamo automated modeling [171]. Ma et al. extended information models of earthquake-damaged reinforced-concrete structures based on IFC, providing a data-structure reference for damage processes, component relations, and geometric evolution [172]. Ouyang et al. proposed a pixel-to-infrastructure digitization workflow for tunnel cracks, realizing spatial distribution and length/width quantification through SFM, mesh fitting, orthographic unfolding, and deep-learning detection [173]. Zhu et al. combined deep-learning 3D reconstruction with damage detection for surface-damage recognition and localization on historic buildings [174]. Together, these studies show that 3D mapping of 2D results has a methodological basis, but reliability still depends on reconstruction quality, coordinate transformation, and the observability of target surfaces.

Point-cloud density determines the lower bound of 3D detectability. Millimeter-scale cracks rarely produce stable geometric signatures in sparse SFM point clouds and therefore often depend on high-resolution texture maps. Point-cloud learning methods such as PointNet, PointNet++, and RandLA-Net are suitable for classification or segmentation only when the point cloud has sufficient density and quality [175,176,177]. For fine cracks, 3D models therefore serve mainly for localization and component association, whereas spalling, deformation, or missing finish layers are more likely to be quantified directly from point clouds.

#### 4.5.3. Engineering Quantification and Digital Twin Updating

Once in 3D space, defect results must be converted into length, width, area, position, component ID, status updates, and repair priority. Torok et al. reconstructed crack contours on 3D meshes and measured geometric features [178]; Yu et al. used depth, intensity, and local PCA features of UAV point clouds to segment and quantify cracks and spalling on post-earthquake building facades [179]; Deng et al. combined SAM-refined segmentation with visual–inertial LiDAR fusion for 3D reconstruction and automatic measurement of concrete cracks [180]. However, spalling depth, defect volume, and component-ID association remain harder to automate, requiring assistance from high-density point clouds, structured light, laser scanning, IRT, ultrasonics, or GPR. SDE-BIM and UAV-to-BIM mapping have demonstrated the feasibility of projecting defects onto BIM surfaces [34,171], but under multi-material facades, complex components, and occlusion, component-level automatic association still generally requires manual review or semi-automatic correction.

From the perspective of the engineering closed loop, facade defect recognition should serve “detection–localization–rating–repair.” Visual saliency does not equal engineering severity: elongated surface cracks may be visually conspicuous yet pose limited structural risk, whereas localized delamination or loose tiles may appear visually weak yet carry falling hazards. Crack width, spalling area, delamination position, seepage extent, and component type must therefore be further mapped to engineering ratings and repair priorities. Research on BIM for existing buildings points out that high modeling cost, difficult information updating, and inadequate handling of uncertain data remain major obstacles [181]; digital twins for structural health monitoring likewise require explicit geometric-accuracy requirements [182]. Kim et al. and Golparvar-Fard et al. demonstrated the potential of automatic matching between 3D data and BIM, but mainly for construction-progress monitoring of new buildings rather than existing facade maintenance [183,184]. The key to facade digital twins is therefore not merely building 3D models but continuously updating defect status and converting detection results into actionable maintenance-management information.

Table 10 summarizes representative pathways for 2D-to-3D engineering localization, including SFM, BIM registration, point-cloud segmentation, LiDAR quantification, and digital-twin updating, and reports reprojection, registration, and measurement errors together with mIoU and processing time. Most studies remain at T3/T4 and S1/S2, and few systems reach T5, S3, and M3 simultaneously. Cases approaching M3 include climbing robots linked to synchronized digital twins and selected GeoBIM frameworks. The former integrate robotic platforms, RTK, deep-learning segmentation, position updating, and centimeter-level quantification [36], while the latter achieve centimeter-level registration and support large-building management [169]. M3 depends less on any single metric than on continuous acquisition, coordinate unification, component-ID binding, status updating, review interfaces, and repair-information output. Sustained integration of AI, 3D reconstruction, and BIM also requires reusable components, field-review interfaces, and component-semantic models [185,186,187,188,189].

The 2D-to-3D workflow is progressing from proof of concept toward operational tools. UAV-SFM provides flexible, low-cost coverage; LiDAR offers more reliable geometry for verification and measurement; and BIM or GeoBIM adds component semantics and an O&M information structure. Future studies should evaluate the complete error chain in Table 9, relate point-cloud density to detectable defect size, account for grazing views and occlusion in flight planning, and link mapped defects to facade condition ratings.

### 4.6. Interpretability, Physical Constraints, and Boundaries of Foundation Models

Detection accuracy alone does not establish trustworthiness. When model outputs inform repair, verification, or area closure, users must also know whether the model responds to the defect rather than to a correlated background feature. Grad-CAM, LIME, and SHAP can reveal sensitive image regions; physics-informed or physics-guided methods can improve plausibility; and SAM, CLIP, and vision-language models can reduce the effort required for annotation, retrieval, and reporting. None of these tools can support repair decisions without material context, environmental data, spatial localization, and engineering rules.

#### 4.6.1. Applicability Boundaries of Post Hoc Explanation Methods

Grad-CAM can generate class-relevant saliency maps and is often used to judge whether a model attends to crack, spalling, or thermal-anomaly regions [190]. In facade scenarios, however, saliency maps can only show that the model’s output is sensitive to certain regions and cannot prove that the model relies on genuine defect mechanisms. Brick joints, window-frame edges, drainage stains, shadows, and soiling often resemble crack or seepage boundaries, and Grad-CAM can hardly distinguish whether the model exploits defect morphology or misuses co-occurring background textures.

LIME and SHAP explain predictions through local surrogate models and Shapley values, respectively [191,192], but superpixel perturbation, local sampling, and approximate contribution computation in high-resolution facade images are affected by repetitive textures, elongated cracks, and complex backgrounds. XAI reviews in the SHM field also point out that no general explanation method yet simultaneously satisfies engineering interpretability, stability, and deployability [193].

Facade studies have begun to use these tools for model checking. Fallahy and Rezazadeh combined LIME, Grad-CAM, and occlusion-sensitivity analysis to test whether a masonry-crack model relied on crack structures rather than background texture [194]. Wang et al. paired XAI with UAV thermography to examine debonding predictions from a small infrared dataset [40]. Post hoc explanation can support verification, but it does not establish engineering validity. A more reliable assessment combines saliency maps, occlusion tests, cross-domain validation, and manual review, and checks whether explanations remain consistent across materials, lighting, and defect types.

#### 4.6.2. Physical Constraints and Domain Adaptation

Physics-informed machine learning attempts to introduce structural mechanics, material degradation, or heat-conduction laws into models to improve stability under small samples and out-of-domain conditions. PIML can constrain model outputs through physics-inspired features, physics-guided data manipulation, regularization losses, or prior constraints, and has been used in SHM, bridge damage detection, and structural failure analysis to enhance interpretability and generalization [195,196,197]. However, when transferring these constraints to building facades, the combined effects of multiple materials, multiple scales, nonlinear degradation, and environmental noise must be confronted; physical assumptions from adjacent structural scenarios cannot simply be imposed on facade defects.

Physics-informed neural networks (PINNs) commonly impose governing equations as soft constraints by adding PDE residuals and initial or boundary conditions to the loss function [198]. Hard constraints instead satisfy selected conditions exactly through the architecture or solution representation. PINNs are most effective when governing equations are well defined, but facade defects cannot always be reduced to a single PDE. Cracks, delamination, debonding, seepage, and finish detachment are often jointly affected by material aging, hygrothermal cycling, construction quality, interface bonding, and environmental exposure; directly transferring bridge fatigue-crack models or idealized heat-conduction equations may impose inappropriate constraints and hinder learning.

Building facades are therefore better suited to weak physical constraints than to forcibly embedding complete equations. For example, infrared debonding detection can take solar radiation, wind speed, ambient temperature difference, and acquisition time as model inputs or confidence-modulation factors. The study by Chen et al. on infrared thermography of facade debonding shows that environmental variables significantly affect thermal contrast and the effective detection window [199]. A more feasible future direction is to incorporate material properties, orientation, acquisition time, and environmental parameters into dataset metadata, and to involve them in model inference in the form of output plausibility checks, confidence calibration, or multimodal fusion weights.

#### 4.6.3. Usable Boundaries of Vision Foundation Models

Vision foundation models such as SAM, CLIP, LLaVA, and MiniGPT-4 provide new tools for facade inspection, but their engineering boundaries must be distinguished. SAM provides general-purpose segmentation capabilities [121]. Yet facade cracks are often elongated, open-ended, and blur-bounded, while debonding and delamination lack stable RGB textures. CrackSAM, through parameter-efficient fine-tuning and the construction of a road-and-facade crack dataset, demonstrates that foundation models still require task adaptation [122]. In practical inspection, SAM mostly relies on upstream detectors for prompts and is better suited to fine segmentation of candidate regions than to serving as an independent detection model.

VLMs are most useful for semantic retrieval and report generation. CLIP can assist category expansion and image retrieval [200]. LLaVA and MiniGPT-4 connect visual encoders to large language models for image captioning, visual question answering, and report drafting [201,202]. Construction-hazard recognition frameworks further show that image interpretation can be converted into structured text [203]. Facade repair, however, requires length, width, area, component position, and grading; without detection masks, 3D localization, and engineering rules, general-purpose VLMs may produce generic or hallucinated descriptions.

Foundation models are best positioned as complementary tools in facade inspection rather than replacements for dedicated systems. SAM can support interactive or semi-automatic refinement of candidate defects, CLIP can support image retrieval and category prompting, and VLMs can assist report drafting, explanations for manual review, and knowledge queries. Quantitative detection, 3D mapping, and repair rating should remain the responsibility of dedicated models and engineering-rule systems validated on facade data with controlled error.

Table 11 defines the roles of interpretability, physical constraints, and foundation models in facade-defect recognition. These methods supplement the T1–T5 workflow by supporting model checks, physical plausibility, annotation, and reporting; they do not replace the task hierarchy.

Interpretability, physical constraints, and foundation models should be treated as supporting layers rather than substitutes for the T1–T5 workflow. Saliency and perturbation analyses can expose questionable model attention but do not establish causal validity. Physical constraints can improve plausibility only when they reflect the materials and environment of the facade. Foundation models may reduce annotation and reporting effort, but quantitative detection and repair assessment still require dedicated models, controlled errors, and manual review.

## 5. Conclusions and Outlook

### 5.1. Conclusions

This review synthesizes progress in intelligent building-facade defect recognition across data acquisition, annotation, single-modality vision, multimodal fusion, 3D engineering localization, and the boundaries of interpretability and foundation models. The evidence indicates that combining deep learning with UAV inspection, infrared thermography, point-cloud reconstruction, and BIM or digital-twin technologies has improved automated recognition of cracks, spalling, delamination, debonding, seepage, and thermal anomalies. The field, however, has not yet established a reliable transition from image-based defect detection to engineering repair decisions. The limiting factors extend beyond the accuracy of individual models to the entire chain of data generation, annotation granularity, modality alignment, spatial mapping, physical interpretation, condition rating, and field validation.

Across the 106 visual-technical studies, the evidence spans a pathway from T1 classification and T2 detection through T3 segmentation and T4 geometric quantification to T5 3D localization/reconstruction. As summarized in Figure 4, T3 was the most common highest output (45 studies, 42.5%), whereas T4 remained comparatively uncommon (9, 8.5%). F0 single-modal methods also remained dominant (71, 67.0%), while F1–F3 fusion was used to integrate complementary evidence for weakly visible defects, including delamination, debonding, seepage, and thermal anomalies. S2 point-cloud or mesh registration and S3 BIM or digital-twin binding provide the spatial basis for component-level localization; however, only 20 studies reached T5 and 8 reached S3. Most studies remained at M1 (59, 55.7%) or M2 (46, 43.4%), and only one met the combined T5/S3/M3 criteria. The principal gap therefore lies not in isolated recognition accuracy or 3D demonstrations, but in maintaining calibration, uncertainty control, component association, review, maintenance action, and model updating across the complete O&M chain.

The principal bottleneck is whether algorithm outputs can be converted into component-level, quantifiable engineering information for repair decisions, rather than whether the models are sufficiently sophisticated. Image classification and bounding-box detection support large-scale screening and pre-localization but seldom provide the geometric detail required for repair. Pixel-level segmentation supports length, area, and boundary extraction but remains sensitive to annotation consistency, imaging conditions, and domain shift. Multimodal fusion can add thermal-response and geometric information, although its value depends on registration error, defect mechanism, and environmental conditions. Once 2D results enter 3D space, camera calibration, point-cloud/BIM registration, component matching, and error-propagation control are still required before repair quantities or model updates can be generated. Interpretability tools, physical constraints, and foundation models have supporting roles along this chain but cannot replace dedicated detection, quantification, and condition-rating systems with controlled error.

### 5.2. Research Challenges and Future Outlook

Future research should move beyond single-model accuracy comparisons and establish evidence protocols that support closed-loop engineering use. In line with the datasheets-for-datasets approach, facade datasets should record the acquisition platform, distance, material, orientation, floor level, illumination, thermal environment, annotation rules, failure cases, and recommended usage boundaries in addition to image counts, category counts, and random-split accuracy [204]. Cross-building, cross-material, cross-city, and cross-sensor evaluations become interpretable only when data provenance and applicability are documented fully.

Unified evaluation protocols are essential for moving from algorithm studies to engineering applications. Future facade-recognition studies should report accuracy, mAP, and mIoU together with deployment efficiency, geometric error, cross-domain performance degradation, and failure cases. Model cards provide a useful structure for documenting training data, test scenarios, hardware, input resolution, applicability boundaries, and unsuitable conditions [205]. Routine reports should also include cross-dataset performance loss, domain shift, uncertainty, and calibration error across materials, floor levels, acquisition distances, and thermal environments. These uncertainty estimates can guide manual-review priority, edge–cloud data transfer, and flagging of high-risk regions [206,207].

Future multimodal-fusion research should establish quantitative relationships among defect mechanisms, registration errors, thermophysical conditions, and fusion levels rather than simply adding sensors. Multisensor-fusion theory defines fusion as the multilevel combination of complementary information to reduce decision uncertainty [208]. The statistical pattern-recognition framework in SHM likewise treats damage identification as a closed loop of data acquisition, feature extraction, pattern classification, and decision interpretation [209]. For facades, the choice among F1, F2, and F3 should be determined jointly by physical response, environmental conditions, and registration quality. Reports should include RGB-IRT registration error, modality-conflict proportion, ambient temperature, solar radiation, material emissivity, acquisition time, and edge-deployment cost.

Transforming 2D recognition results into 3D engineering space remains a weak link in the closed loop. UAV-SFM, LiDAR, BIM, and digital-twin methods can support visualization and localization, but most workflows rarely extend from detection, localization, and display to rating, repair, and updating. Research on BIM for existing buildings identifies high modeling costs, difficulty updating information, and inadequate handling of uncertainty as persistent problems [181]. Digital twins for SHM also require explicit geometric-accuracy specifications [182]. A standardized 2D-to-3D mapping-error protocol should therefore report camera-intrinsic and extrinsic errors, reprojection error, point-cloud/BIM registration error, component-matching accuracy, and 3D RMSE separately, and should link crack width, spalling area, delamination position, seepage extent, and component type to maintenance grades and repair priorities.

Interpretability, physical constraints, and foundation models should remain supporting layers for trustworthy deployment. Grad-CAM, LIME, and SHAP can help determine whether models attend to anomalous regions but cannot prove reliance on genuine defect mechanisms. PIML and PINNs can improve physical consistency, although no unified governing equation covers the multimaterial, multidefect, and multienvironment conditions of facades. SAM, CLIP, and VLMs can assist annotation, candidate-region refinement, image retrieval, and report drafting but cannot replace dedicated detection, 3D-mapping, and condition-rating models. A practical future architecture is a layered closed loop comprising dedicated detection, multimodal spatial localization, physical metadata, uncertainty estimation, manual review, and report generation.

Several limitations concern the review process. Title–abstract screening, full-text screening, and data extraction were led by one reviewer, although uncertain cases were discussed with co-authors. Source-specific historical search strings, database-level retrieval counts, the deduplication software log, and a mutually exclusive reason-level tally for the 247 full-text exclusions were not completely retained. The structured evidence flags improve comparison but remain dependent on what each source reported; NR cannot establish that an attribute was absent. The corpus also combines direct facade evidence, transferable infrastructure methods, enabling sources, and contextual references. No pooled effect estimate or universal risk-of-bias score was therefore calculated, and the reported counts should be interpreted as a transparent descriptive synthesis rather than an estimate of comparative model effectiveness.

In summary, progress in intelligent facade-defect recognition depends on four foundations in addition to advances in network architecture and sensor integration: dataset protocols that document acquisition conditions, material properties, annotation boundaries, and intended use; evaluation protocols that jointly report recognition performance, deployment efficiency, geometric error, and cross-domain degradation; multimodal-fusion specifications that include registration error, modality conflict, and differences in physical response; and 2D-to-3D mapping-error protocols that reliably convert segmentation masks into component-level 3D engineering information. Intelligent facade inspection can support large-scale engineering deployment only when algorithm outputs can be verified, quantified, and updated reliably on real buildings, platforms, and maintenance workflows.

## Figures and Tables

**Table 1:** Unified coding scheme and analytical dimensions.

Dimension	Symbol	Definition	Main Purpose
Annotation granularity	A0–A2/A2*	A0: image-level labels; A1: bounding boxes; A2: pixel-level masks; A2*: dense point-level defect labels	Indicates annotation precision and quantifiability of the data
Visual task hierarchy	T1–T5	T1: classification; T2: detection; T3: segmentation; T4: geometric quantification; T5: 3D localization/reconstruction	Indicates whether model outputs can support engineering quantification
Fusion level	F0–F3	F0: single modality; F1: image-level fusion; F2: feature-level fusion; F3: decision-level fusion	Indicates how multimodal information is combined
Spatial mapping level	S0–S3	S0: no spatial mapping; S1: image coordinates; S2: point cloud/mesh; S3: BIM/digital twin	Indicates the degree to which detection results enter engineering space
Engineering maturity	M1–M3	M1: proof of concept; M2: controlled field trials; M3: engineering deployment	Indicates proximity to a closed-loop field O&M workflow

**Table 2:** Common evaluation metrics for building facade defect recognition.

Metric Category	Common Metrics	Applicable Tasks	Notes
Classification performance	Acc, P/R, F1, PPV, TPR/FPR	Image-level screening, anomaly judgment	Accuracy may overstate performance under class imbalance
Detection performance	IoU thresholds, AP/AP50, mAP@0.5, mAP@0.5–0.95, P/R/F1	Object detection, box-level localization	Box-level metrics may mask background redundancy for elongated cracks
Segmentation performance	IoU/mIoU, Dice/DSC, PA/mPA, F1, AP/mAP	Semantic/instance segmentation	Boundary quality and small-object connectivity require separate attention
Geometric quantification	Length/width/area error, AE/RE, GSD, scale error	Estimation of crack length, spalling area, delamination extent	Pixel scale, camera parameters, and post-processing must be specified
3D localization	Reprojection error, registration RMSE, 3D RMSE, point-to-point/plane error, component matching accuracy	Point cloud, mesh, BIM/digital twin mapping	Grazing angles, point cloud density, and component matching affect the results
Deployment efficiency	FPS, latency, processing time, Params, FLOPs/GFLOPs, model complexity	UAV/robot/edge-device inference	Hardware platform, precision mode, and batch size should be reported
Generalization and reliability	Cross-dataset changes in Acc/mAP/IoU/F1, performance degradation, transfer gain, failure cases	Cross-building, cross-material, cross-city testing	Random splits of a single dataset are insufficient to demonstrate engineering generalization

**Table 3:** Representative applications of the coding framework.

Study	Defect Type	Sensor Modality	CV Task	Fusion Level	Spatial Mapping Level	Maturity
Cha et al. (2017) [25]	Cracks	RGB	T1 classification	F0 single modality	S0 no spatial mapping	M1 proof of concept
Dorafshan et al. (2018) SDNET2018 [26]	Cracks	RGB	T1 classification	F0 single modality	S0 no spatial mapping	M1 proof of concept
Mundt et al. (2019) CODEBRIM [27]	Multi-class	RGB	T1 classification	F0 single modality	S0 no spatial mapping	M1 proof of concept
Wei et al. (2023) BFD-YOLO [28]	Multi-class	RGB	T2 detection	F0 single modality	S1 image coordinates	M2 controlled field
Zhou and Tiong (2026) [29]	Multi-class	RGB	T3 segmentation	F0 single modality	S1 image coordinates	M2 controlled field
Liu et al. (2026) IRT [30]	Delamination/seepage	IRT	T2 detection	F0 single modality	S1 image coordinates	M2 controlled field
Wang et al. (2025) fusion-based [31]	Multi-class	RGB + IRT	T3 segmentation	F1 → F2 image-to-feature-level fusion	S1 image coordinates	M2 controlled field
He et al. (2025) WCL [32]	Delamination/debonding	RGB + IRT	T3 segmentation	F2 feature-level fusion	S1 image coordinates	M2 controlled field
SCSegamba (2025) [33]	Cracks	RGB	T3 segmentation	F0 single modality	S1 image coordinates	M1 proof of concept
Yang et al. (2024) Defect-BIM [34]	Multi-class	RGB + SFM	T3 + T5 segmentation and 3D mapping	F1 image-level fusion	S3 BIM/digital twin	M2 controlled field
Pantoja-Rosero et al. (2023) [35]	Multi-class	RGB + 3D	T3 + T5 segmentation and 3D mapping	F1 image-level fusion	S2 point cloud/mesh	M2 controlled field
Song et al. (2026) climbing-robot DT [36]	Multi-class	RGB + 3D	T3 + T5 segmentation and 3D mapping	F3 decision-level fusion	S3 BIM/digital twin	M3 engineering deployment

**Table 4:** Overview of facade material systems, typical defects, and implications for annotation and modality selection.

Material/System	Key Imaging Cues	Typical Defects and Triggers	Annotation/Modality Implications
Panel cladding (metal, aluminum composite, stone, fiber cement)	Large regular panels; clear joints, corners, anchors, and frames; reflective or textured surfaces	Seepage, joint cracking, deformation, corrosion, delamination, loosening, detachment; driven by thermal movement, wind suction, rain, sealant/anchor aging, and freeze–thaw	RGB: joints, cracks, corrosion, detachment; IRT: hidden delamination/moisture; LiDAR/3D: deformation and component position
Tile/facing-brick systems	Repetitive small units and mortar-joint grids; glaze reflections and regular textures	Debonding, cracks, tile loss, seepage, efflorescence, mold; driven by bond aging, substrate movement, moisture, salts, and freeze–thaw	Treat joints and decorative lines as hard negatives; distinguish joints, cracks, and debonding; combine IRT or tapping for hidden defects
Paint, render, and EIFS/EWI	Continuous finish with roller, trowel, mortar, or color-variation textures	Surface cracks, peeling, blistering, spalling, seepage, chalking, efflorescence, mold, insulation debonding; linked to shrinkage, hygrothermal/UV aging, retained moisture, and bond failure	RGB: visible surface defects; IRT: moisture, insulation defects, thermal bridges; use soft boundaries for gradual anomalies
Masonry, fairfaced brick, and natural stone	Blocks and mortar joints; porous surfaces with variable color, weathering, salts, and biological growth	Joint/wall cracks, erosion, efflorescence, seepage, freeze–thaw spalling, biological soiling; driven by capillary uptake, rain, salts, freeze–thaw, and material mismatch	Include joints, stone texture, and salts as hard negatives; use material-aware labels; expect difficult cross-material transfer
Glass curtain walls	Reflective or transparent glass with visible frames, sealants, and joints	Glass failure, sealant aging, seepage, condensation, thermal bridges, frame/fastener anomalies; linked to wind, thermal movement, UV exposure, drainage, and sealing failure	RGB is reflection-sensitive; IRT better screens thermal bridges, seepage, and seal failure; annotate component joints rather than color differences

Note: The defects listed in the table are common engineering generalizations and do not imply that all buildings follow a fixed development path. Actual defect formation is also affected by construction quality, climate zone, orientation, floor height, construction details, maintenance history, and service environment.

**Table 5:** Overview of datasets and evidence sources for building facade defect detection and related transferable domains.

Dataset/Study	Modality/Platform	Target and Annotation	Scale/Access	Main Evidence
SDNET2018 (2018) [26]	RGB; structural surfaces	Crack/non-crack; A0	56,000+; public	Image-level labels with difficult backgrounds
CODEBRIM (2019) [27]	RGB; handheld/UAV	Multiclass concrete defects; A0	1590; public	Multiclass defects mixed with background variation
Multitemporal oblique imagery (2019) [39]	RGB; aerial oblique photogrammetry	Facade damage; A2	Two campaigns (2008/2009), 27 validation facades; not public	Pose repeatability and temporal change
Wall Crack Multiclass (2022) [45]	RGB; ground	Wall-crack subtypes; A0	2516; public	Expert-defined crack categories
Masonry-wall segmentation (2022) [48]	RGB; ground	Masonry cracks/bricks; A2	2814; partial access	Pixel-level geometric evidence
CUBIT-Det (2024) [46]	RGB; unmanned systems	Multiclass infrastructure defects; A1	5500+; public	High-resolution cross-structure detection
Thermal facade segmentation (2024) [49]	IRT + RGB/MSX; ground	Components and thermal bridges; A2	577 thermograms, 118 RGB, 114 MSX; public	Thermal components and pixel-level anomalies
UAV residential facades (2025) [37]	RGB + IRT; UAV	Cracks/seepage; A2	Not reported; not public	Distance, pose, and modality tradeoffs
Multimodal facade damage (2025) [31]	RGB + IRT; facade/UAV	Multiclass damage; A2	1761 pairs; on request/not public	Registration and end-to-end fusion
UAV building-surface defects (2025) [47]	UAV RGB	Cracks, detachment, seepage, corrosion, blistering; A1	14,471; public	Open facade-oriented multiclass benchmark
Envelope thermal anomalies (2025) [50]	IRT; UAV	Thermal anomalies/components; A2	701; research data	Joint component and anomaly labels
Color-to-thermal anomalies (2025) [52]	RGB + IRT; vehicle/street view	Thermal/moisture anomalies; A2	948 + 50, 865 + 50, 184 + 9, plus 30 blind-test pairs; availability unverified	Paired anomaly detection with sparse labels
High-rise facade IRT (2026) [30]	IRT; UAV	Delamination, seepage, cracks, detachment; A1/A2	180; not public	Thermal diffusion and small objects
UAV thermography + XAI (2026) [40]	IRT; UAV	Debonding; A1/A2	455; not public	Limits of thermography and post hoc explanation
Cross-dataset crack classification (2025) [53]	Multiple RGB datasets	Structural cracks; A0	Six public datasets; verifiable	Cross-dataset performance loss
DeepCrack (2019) [62]	RGB; multiscene	Cracks; A2	537; public	Hierarchical pixel segmentation
CrackSeg9k (2023) [63]	Multi-source RGB	Cracks; A2	~9000; public	Dataset aggregation and protocol alignment
StructDamage (2026) [64]	RGB; multiple structures	Cracks/surface defects; A0	~78,093; preprint dataset	Large cross-surface benchmark
Facade characteristics (2024) [56]	RGB; street view	Facade type, storeys, materials; A0	5.05 GB, 640 × 640 PNG; public	Material/scene pretraining rather than defect labels
PointCrack3D (2021) [58]	LiDAR/3D	Cracks; A2* point level	21 colored scans, >900 m^2^, 412 cracks; access unverified	3D crack localization and geometry
3D realitymesh cracks (2020) [57]	RGB/SFM-3D	Cracks; A1/A2	1250; restricted	2D recognition mapped to 3D meshes

Note: A2* denotes dense point-level labels, used here as the 3D analogue of pixel-level masks.

**Table 7:** Sensor modality–facade defect physical response matrix.

Defect Type	RGB Visible Light	Infrared Thermography	LiDAR/Depth	Ultrasonics/GPR	Fusion Value
Cracks	High: texture and edges visible	Low–medium: thermal diffusion blurs boundaries	Low: insufficient resolution for fine cracks	Medium: assists with deep cracks	RGB dominant; IRT/NDT supplement anomaly interpretation
Delamination/debonding	Low: weak early appearance	High: clear thermal-response differences	Low: surface deformation may be subtle or inconsistent	Medium–high	IRT dominant; RGB provides component semantics and pseudo-anomaly exclusion
Spalling/finish detachment	High: clear regional boundaries	Medium: local thermal-response variation	High: depth and volume quantification	—	RGB for detection; LiDAR/depth for geometric quantification
Seepage/moisture damage	Medium: water stains visible but easily confused	High: evaporative cooling and moist-area anomalies	Low	—	IRT for localization; RGB confirms surface semantics
Thermal bridges/thermal anomalies	Low	High: temperature gradients directly visible	—	—	IRT dominant; RGB for component localization
Rebar corrosion	Low–medium: surface rust stains visible	Medium: local thermal-property changes	—	High	NDT dominant; RGB/IRT assist surface verification

**Table 8:** Comparison of multimodal fusion methods for building facades and related domains.

Study/Modalities	Fusion and Target	Key Verifiable Evidence	Engineering Interpretation
Wang et al. [31], RGB + IRT	F1 → F2; facade damage; T3/S1	1761 pairs; registration RMSE 14.35 px; best mAP 85.4%; 35.661 M, 42.680 G FLOPs, 0.047 s	End-to-end workflow is feasible; registration still limits narrow defects
Wang et al. [153], RGB + IRT	F1; deterioration; T3/S1	1228 pairs; mAP 86.5%; class AP 79.9–91.5%; 0.423 s/frame	Near-real-time early fusion; thermal context and registration remain limiting
He et al. WCL [32], RGB + IRT	F2; subsurface defects; T3/S1	Pixel Acc 98.84%, IoU 0.7145, F1 ≈ 0.78, <6.7 s/image; HKUST validation	Designed for weakly aligned UAV data
He et al. LBF2-Net [130], RGB + IRT	F2; tiled-facade defects; T3/S1	1054 pairs (~68% defective); IoU 0.8115, F1 0.7819, Pixel Acc ≈ 98%	Separates shared and modality-specific responses
Yang et al. [147], RGB + IRT	F1/F2/F3 comparison; multiclass defects; T2/S1	Best Acc > 99.6%, F1 98.0%, AP 0.9744; class AP 86.0–91.5%	Fusion level is task-dependent; unsuitable for cross-dataset ranking
Motayyeb et al. [154], RGB + IRT + point cloud	F1; thermal-leakage mapping; T3–T5/S2	P/R 87/90% and 87/88% on two datasets; SFM/MVS + ICP	Links thermal anomalies to 3D position
Gao et al. [155], RGB + IRT	F2/correlation; delamination	F1 0.88, PPV 91%, TPR 85%, Acc 98.8%, FPR 0.4%	RGB semantics can reject thermal pseudo-anomalies
Liu et al. [30], IRT	F0; detection/segmentation	Detection P/mAP50/R/F1 89.7/87.9/81.8/85.6%; segmentation mIoU 78.83%, F1 87.47%	Requires wall-background filtering and small-object handling
Elamin et al. [156], RGB + LiDAR	F1 geometry; pavement cracks	P/R/F1 77.48/87.66/82.26%; low-grade LiDAR reduced fine-crack precision	Additional sensors help only when quality and scale are adequate
Aljagoub et al. [157], IRT + simulation	Augmentation; bridge-deck delamination	P/R/F1 94.5/86.4/90.2%, mAP50 89.7%, mAP50–95 72.7%; depth > 132 mm	Supports simulation-based augmentation when IRT data are scarce
DMM [158], RGB + IR	F2 Mamba; oriented detection	P/R/AP50/AP50–95 86.2/82.5/90.7/61.0%, 128 FPS; AP50 +4.8% vs. IR-only	Lightweight transfer reference; not facade-validated
MambaRefine-YOLO [159], RGB + IR	F2 Mamba; UAV small objects	DroneVehicle mAP 83.2%, +7.9% vs. baseline	Edge potential; facade validation pending

Note: The table adopts only originally reported results that can be stably verified and is not intended for cross-dataset performance ranking.

**Table 9:** Error propagation chain from 2D recognition to 3D engineering localization and recommended reporting metrics.

Stage	Main Error Sources	Downstream Propagation Effects	Recommended Metrics or Control Measures
Image acquisition and camera calibration	Focal length, distortion, GSD, rolling shutter, exposure, and viewpoint changes	Affects pixel scale, mask boundaries, and back-projection ray directions	Calibration residuals, GSD, reprojection error, records of shooting distance and viewpoint
SFM/MVS and point-cloud generation	Feature-matching errors, bundle-adjustment residuals, MVS dense-matching uncertainty	Offsets in point-cloud coordinates, surface normals, and texture-map positions	BA RMSE, point-cloud accuracy, point-to-point/point-to-plane error, checkpoint errors
Multimodal and 3D registration	RGB-IRT field-of-view differences, extrinsic errors, time desynchronization, LiDAR/image coordinate transformation errors	Misalignment of thermal anomalies, texture boundaries, and geometric surfaces	Registration RMSE, time offsets, synchronization strategies, modality-conflict proportion
BIM/mesh component matching	Insufficient BIM LOD, missing component semantics, ICP or semantic-matching errors	Defects assigned to incorrect components, affecting repair responsibility and work-order generation	BIM/point-cloud registration error, component matching accuracy, manual review ratio
Mask back-projection and geometric quantification	Grazing angles, occlusion, surface intersection errors, pixel-boundary noise, and post-processing errors	Accumulated errors in crack length, width, spalling area, and position	3D RMSE, length/width/area error, confidence intervals, review thresholds

**Table 10:** Comparison of 3D reconstruction and digital twin methods.

Method/Route	Levels	Key Verifiable Evidence	Engineering Role and Limitation
Damage-augmented digital twin [35]: SFM + segmentation	T3–T5/S2/M2	Crack F1 ≈ 0.68; FRDS LOD2 0.86–0.97, LOD3 0.84–0.94; IMF 0.0125–0.0839 m	3D damage representation; component O&M rules incomplete
UAV image-to-BIM [167]: GHT + BIM view matching	T5/S3/M2	Mean error ≈ 6 px (10–16 mm); maximum 20–24 mm; GPS baseline ≈ 173 mm	Facade localization; depends on BIM geometry
SDE-BIM [34]: UAV + K-Net + texture mapping	T3–T5/S3/M2	Training IoU ≈ 75%; other metrics ≈ 85%; ~0.5 s/patch; south facade ~14 min detection + 42 min mapping	Maps image U–V results to BIM coordinates
Tunnel digitization [173]: SFM + unfolding + DL	T3–T4/S2/M2	GSD 0.25–0.26 mm/px; scale error < 2 mm/m; length error 0.63%; transferred mIoU 87.92%	Transferable to unfolded facades; tunnel geometry is simpler
Historic-building localization [174]: 3D reconstruction + segmentation	T3–T5/S2/M2	4213 detection labels, 4710 masks; F1 0.80, mAP50 0.839; Acc/mPA/mIoU 98.41/89.56/83.72%; 119 maps for 3D localization	Handles complex heritage geometry; performance depends on data-quality
Realitymesh cracks [57]: oblique photogrammetry + FRCNN/Mask R-CNN	T3–T4/S2/M2	AP 0.78, DSC 0.54; crack-width grading and 3D display	Visualization and quantification; fine cracks limited by mesh resolution
LiDAR facade segmentation [161]: RandLA-Net	T5/S2/M2	XYZ + corrected intensity: mean Acc 98.87%, mIoU 83.1%; +6.94% mIoU vs. XYZ	Reliable geometry; weak defect-texture representation
Climbing-robot digital twin [36]: robot + RTK + DL	T3–T5/S3/M3	BBox mAP50/mAP75 0.823/0.708; segmentation mAP 0.775; F1 0.807; length error 1.140 cm	Near closed-loop deployment; complex platform and limited scenarios
GeoBIM digital twin [169]: UAV + semantic registration	T5/S3/M2–M3	Mean horizontal/vertical/diagonal errors 0.490/0.592/1.360 cm	Large-building management; standardized error protocols remain necessary
SAM + LiDAR measurement [180]: segmentation + LiDAR/IMU	T3–T5/S2/M2	Few-shot IoU improved; 15 cracks measured with AE 0.09 mm, RE 14.37%	Curved-surface crack measurement; limited facade multiclass validation

Note: T3, T4, and T5 denote pixel-level segmentation, geometric quantification, and 3D localization/reconstruction, respectively; S2 and S3 denote point-cloud/mesh and BIM/digital-twin mapping; M2 and M3 denote controlled field trials and engineering deployment. The table includes only information that could be verified in the original publications or accessible records and is not intended for cross-method ranking.

**Table 11:** Comparison of interpretability, physical-constraint, and foundation-model methods.

Category/Study	Role and Level	Maturity	Main Limitation
Grad-CAM [190]	Class-sensitive regions; T1–T3 verification	M1–M2	Sensitivity is not causal grounding
LIME [191]	Model-agnostic local explanation; T1–T3	M1	Superpixel and sampling instability
SHAP [192]	Feature attribution; T1–T3	M1	High image-dimensional cost; weak spatial semantics
SHM XAI review [193]	Trustworthy-deployment requirements	M1–M2	No method satisfies all engineering requirements
MARBLE-DA [194]	Domain adaptation with explanation; T1–T3	M2	Needs agreement across multiple explanation tools
UAV-T XAI [40]	Thermographic debonding explanation; T2–T3	M2	Limited by samples and thermophysical context
PIML [195,196,197]	Soft physical constraints; T2–T4	M1–M2	Facade physics are difficult to standardize
PINN [198]	PDE-based constraints; T4 inference	M1	No unified equation for multiple facade defects
SAM/CrackSAM [121,122]	Candidate segmentation and annotation; T3	M1–M2	Unstable zero-shot results; prompts/fine-tuning required
CLIP [200]	Open-vocabulary retrieval; T1	M1	Weak localization and fine-grained discrimination
LLaVA/MiniGPT-4 [201,202]	Captioning and report drafting; T1–T3 support	M1	No quantitative geometry; hallucination risk
VLM report generation [203]	Hazard recognition and structured text	M2	Requires detection, localization, and manual review

Note: Maturity in the table denotes the application stage of the methods in facade or adjacent engineering-vision scenarios, not their technical maturity in the general AI field.

## Data Availability

The author-acquired images are contained in Figure 6; the source-level coding underlying the descriptive synthesis is provided in Supplementary Table S1.

## Supplementary Materials

The following supporting information can be downloaded at: https://www.mdpi.com/article/10.3390/s26175498/s1, Supplementary File S1. Completed PRISMA 2020 checklist; Supplementary Table S1. Complete source-level evidence stratification, A/T/F/S/M coding, evidence-strength flags, and verification notes for the 203 retained sources.

## Appendix A. Search Strategy and Literature Selection

Appendix A documents the information sources, eligibility criteria, screening information, and reconstructed master search strategy referenced in Section 2.1.

### Appendix A.1. Search Sources and Reconstructed Strategy

**Table A1:** Search sources, eligibility criteria, screening information, and reconstructed master search strategy.

Item	Information
Main eligibility period	January 2015–April 2026
Earlier retained sources	Thirteen pre-2015 sources retained only as foundational methods, benchmark datasets, standards, or background evidence
Search and update dates	Final database search: 27 April 2026; targeted policy and contextual source update: 30 June 2026
Main bibliographic databases	Web of Science; Scopus; IEEE Xplore
Publisher platforms	ScienceDirect; SpringerLink; Wiley Online Library; MDPI; SAGE Journals
Supplementary discovery sources	arXiv; CNKI; Crossref; Google Scholar; backward reference searching
Local document library	Used to retrieve and verify previously identified full texts; not treated as an independent bibliographic database
Language limits	English and Chinese
Source types	Journal articles, conference papers, reviews, preprints, datasets, and selected technical or policy sources
Core-study inclusion criteria	Relevant facade, envelope, or transferable near-surface defect; automated recognition, sensing, fusion, or spatial-mapping method; and sufficient data, methods, metrics, or experimental findings
Full-text exclusion criteria	Out-of-scope structure or defect; no relevant automated recognition or mapping pipeline; purely mechanistic or physical NDT study; insufficient methodological or experimental information; duplicate or superseded publication
Historical query status	Database-specific historical search strings were not completely retained; the master strategy below was reconstructed from the documented concepts
Deduplication record	The original software log and complete operational record were not retained; the verified aggregate count of 700 removed duplicates is reported
Screening	One reviewer (C.L.) conducted title–abstract and full-text screening without automated selection tools; uncertain and borderline records were discussed with X.W. and J.L.
Reporting scope	The review is reported with reference to PRISMA 2020 and PRISMA-S; the completed checklist is supplied as Supplementary File S1, and the selection process is reported in Figure 1

### Appendix A.2. Representative Reconstructed Master Search Strategy

**Listing A1.** Representative Reconstructed Master Search Strategy.
( (“building facade*” OR “facade*” OR “building envelope*” OR “external wall*” OR “exterior wall*” OR cladding OR “curtain wall*” OR “masonry facade*” OR “masonry façade*”) AND (defect* OR damage OR deteriorat* OR crack* OR spall* OR delaminat* OR debond* OR detach* OR seepage OR leakage OR moisture OR “thermal anomal*” OR “thermal bridge*” OR corrosion OR efflorescence) AND (“deep learning” OR “machine learning” OR “computer vision” OR UAV OR drone* OR “infrared thermograph*” OR LiDAR OR “point cloud*” OR photogrammetr* OR “structure from motion” OR “semantic segmentation” OR “instance segmentation” OR “3D reconstruction” OR BIM OR “digital twin*” OR “foundation model*” OR “explainable AI”) )
Note: The following master strategy was reconstructed from the documented concept groups to summarize the search scope and Boolean logic. Database-specific syntax was adapted to each platform; therefore, this expression is not presented as a verbatim historical query executed identically across all information sources.

**Table A2:** Reconstructed source-level adaptations of the master search strategy.

Information Source	Role in Search	Reconstructed Field/Syntax Adaptation	Verified Date/Status
Web of Science Core Collection	Principal bibliographic database	Master expression wrapped as a Topic search (TS=)	Final search 27 April 2026; verbatim history unavailable
Scopus	Principal bibliographic database	Master expression wrapped in TITLE-ABS-KEY	Final search 27 April 2026; verbatim history unavailable
IEEE Xplore	Principal technical database	Concept groups applied to All Metadata; technical terms were retained and policy terms omitted	Final search 27 April 2026; verbatim history unavailable
ScienceDirect	Publisher platform	Three concept groups adapted to the platform search interface; shortened combinations used where query length was restricted	Final search 27 April 2026; verbatim history unavailable
SpringerLink	Publisher platform	Three concept groups adapted to the platform search interface	Final search 27 April 2026; verbatim history unavailable
Wiley Online Library	Publisher platform	Facade/defect terms combined with vision, thermography, UAV, or SHM terms	Final search 27 April 2026; verbatim history unavailable
MDPI	Publisher platform	Facade/defect terms combined with vision, thermography, UAV, BIM, or digital-twin terms	Final search 27 April 2026; verbatim history unavailable
SAGE Journals	Publisher platform	Facade/defect terms combined with vision and SHM terms	Final search 27 April 2026; verbatim history unavailable
CNKI	Chinese-language supplementary source	Chinese equivalents of facade/exterior-wall, defect, machine-vision, thermography, UAV, and 3D-mapping concepts	Final search 27 April 2026; verbatim history unavailable
arXiv and Crossref	Supplementary discovery and metadata checking	Shortened combinations of defect target and method terms	Searched through 27 April 2026
Google Scholar	Supplementary discovery	Short phrase combinations and cited-by searching; not used for a reproducible exhaustive count	Searched through 27 April 2026
Backward reference searching	Supplementary discovery	Reference lists of relevant reviews and representative primary studies	Conducted during screening
Government/policy websites	Contextual update only	Targeted document-title and policy-term checks	Updated through 30 June 2026

Note: Table A2 reconstructs source-specific field wrappers and search roles from retained records. It does not represent an exact historical export, and source-level retrieval counts are not reported because the original logs were not retained.
