# Supplementary material for: Intelligent Recognition of Building Facade Defects: A Multilevel Review from Visual Perception to Engineering Operations and Maintenance
Source: Sensors (Basel). 2026 Aug 30;26(17):5498. doi: 10.3390/s26175498 (PMC13568442; doi:10.3390/s26175498)
Supplement: Supplementary file 1 [file sensors-26-05498-s001.zip › Supplementary_File_S1_PRISMA_2020_Checklist.pdf]

PRISMA 2020 Checklist

Section and Topic	Item #	Checklist item	Location where item is reported
**TITLE**			
Title	1	Identify the report as a systematic review.	Title.
**ABSTRACT**			
Abstract	2	See the PRISMA 2020 for Abstracts checklist.	Review scope, coding framework, principal findings, limitations, and implications are reported. Detailed PRISMA methods are in Section 2.1.
**INTRODUCTION**			
Rationale	3	Describe the rationale for the review in the context of existing knowledge.	Introduction, paragraphs 1–6.
Objectives	4	Provide an explicit statement of the objective(s) or question(s) the review addresses.	Introduction, contribution statement and review scope.
**METHODS**			
Eligibility criteria	5	Specify the inclusion and exclusion criteria for the review and how studies were grouped for the syntheses.	Section 2.1, paragraph 3; Appendix A, Table A1.
Information sources	6	Specify all databases, registers, websites, organisations, reference lists and other sources searched or consulted to identify studies. Specify the date when each source was last searched or consulted.	Section 2.1, paragraphs 1–2; Appendix A, Table A1. Final search: 27 April 2026.
Search strategy	7	Present the full search strategies for all databases, registers and websites, including any filters and limits used.	Section 2.1, paragraph 2; Appendix A.1 and Table A1, reconstructed master search strategy.
Selection process	8	Specify the methods used to decide whether a study met the inclusion criteria of the review, including how many reviewers screened each record and each report retrieved, whether they worked independently, and if applicable, details of automation tools used in the process.	Section 2.1, paragraph 4 (single reviewer; co-author discussion of uncertain cases; no automated selection tools).
Data collection process	9	Specify the methods used to collect data from reports, including how many reviewers collected data from each report, whether they worked independently, any processes for obtaining or confirming data from study investigators, and if applicable, details of automation tools used in the process.	Section 2.1, paragraph 5 (single-reviewer structured extraction; no automated extraction tools; no imputation).
Data items	10a	List and define all outcomes for which data were sought. Specify whether all results that were compatible with each outcome domain in each study were sought (e.g. for all measures, time points, analyses), and if not, the methods used to decide which results to collect.	Section 2.1, paragraph 5; Section 2.3 and Table 2.
	10b	List and define all other variables for which data were sought (e.g. participant and intervention characteristics, funding sources). Describe any assumptions made about any missing or unclear information.	Section 2.1, paragraph 5; Sections 2.2–2.3 and Tables 1–3.
Study risk of bias assessment	11	Specify the methods used to assess risk of bias in the included studies, including details of the tool(s) used, how many reviewers assessed each study and whether they worked independently, and if applicable, details of automation tools used in the process.	Final paragraph of Section 2.4. No single formal study-level risk-of-bias instrument was applied; rationale and qualitative appraisal domains are reported.
Effect measures	12	Specify for each outcome the effect measure(s) (e.g. risk ratio, mean difference) used in the synthesis or presentation of results.	Final paragraph of Section 2.4; Section 2.3 and Table 2. Native study metrics were retained and no pooled effect measure was calculated.
Synthesis methods	13a	Describe the processes used to decide which studies were eligible for each synthesis (e.g. tabulating the study intervention characteristics and comparing against the planned groups for each synthesis (item #5)).	Sections 2.1–2.4. Sources were grouped by eligibility, defect, modality, task, and A/T/F/S/M dimensions.
	13b	Describe any methods required to prepare the data for presentation or synthesis, such as handling of missing summary statistics, or data conversions.	Section 2.1, paragraph 5; final paragraph of Section 2.4. Native definitions and units were retained; missing values were not imputed.
	13c	Describe any methods used to tabulate or visually display results of individual studies and syntheses.	Sections 2.2–2.4; Figures 2–4; Tables 1–11.

PRISMA

PRISMA 2020 Checklist

Section and Topic	Item #	Checklist item	Location where item is reported
	13d	Describe any methods used to synthesize results and provide a rationale for the choice(s). If meta-analysis was performed, describe the model(s), method(s) to identify the presence and extent of statistical heterogeneity, and software package(s) used.	Final paragraph of Section 2.4. Structured narrative synthesis was selected because the corpus is methodologically and metrically heterogeneous.
	13e	Describe any methods used to explore possible causes of heterogeneity among study results (e.g. subgroup analysis, meta-regression).	Sections 3–4; qualitative comparisons by defect, modality, task, platform, dataset, and engineering maturity.
	13f	Describe any sensitivity analyses conducted to assess robustness of the synthesized results.	Not applicable: no pooled effect estimate was calculated; therefore, no sensitivity analysis was performed. Stated in Section 2.4.
Reporting bias assessment	14	Describe any methods used to assess risk of bias due to missing results in a synthesis (arising from reporting biases).	Not applicable: no pooled outcome synthesis was performed and reporting bias was not formally assessed. Stated in Section 2.4.
Certainty assessment	15	Describe any methods used to assess certainty (or confidence) in the body of evidence for an outcome.	Not applicable: certainty of evidence was not formally graded. Stated in Section 2.4.
**RESULTS**			
Study selection	16a	Describe the results of the search and selection process, from the number of records identified in the search to the number of studies included in the review, ideally using a flow diagram.	Section 2.1, paragraph 4; Figure 1.
	16b	Cite studies that might appear to meet the inclusion criteria, but which were excluded, and explain why they were excluded.	Section 2.1, paragraphs 3–4 and Figure 1 (verified total of 247 full-text exclusions and principal eligibility categories).
Study characteristics	17	Cite each included study and present its characteristics.	Sections 1–5 and References [1–203]; structured characteristics of representative technical studies in Tables 3–11.
Risk of bias in studies	18	Present assessments of risk of bias for each included study.	Not applicable: no formal study-level risk-of-bias ratings were generated; the rationale is reported in Section 2.4.
Results of individual studies	19	For all outcomes, present, for each study: (a) summary statistics for each group (where appropriate) and (b) an effect estimate and its precision (e.g. confidence/credible interval), ideally using structured tables or plots.	Sections 3–4 and Tables 4–11 report the original study metrics and engineering evidence used in the qualitative synthesis.
Results of syntheses	20a	For each synthesis, briefly summarise the characteristics and risk of bias among contributing studies.	Sections 3–4 and Tables 3–11 summarize contributing-source characteristics and evidence limitations; no formal study-level ratings were generated.
	20b	Present results of all statistical syntheses conducted. If meta-analysis was done, present for each the summary estimate and its precision (e.g. confidence/credible interval) and measures of statistical heterogeneity. If comparing groups, describe the direction of the effect.	Not applicable: no statistical synthesis or meta-analysis was performed.
	20c	Present results of all investigations of possible causes of heterogeneity among study results.	Sections 3–4 report qualitative comparisons across datasets, defects, modalities, tasks, platforms, and maturity levels.
	20d	Present results of all sensitivity analyses conducted to assess the robustness of the synthesized results.	Not applicable: no sensitivity analysis was performed because no pooled estimate was calculated.
Reporting biases	21	Present assessments of risk of bias due to missing results (arising from reporting biases) for each synthesis assessed.	Not applicable: reporting bias was not formally assessed because no pooled outcome synthesis was performed.
Certainty of evidence	22	Present assessments of certainty (or confidence) in the body of evidence for each outcome assessed.	Not applicable: certainty of evidence was not formally graded.
**DISCUSSION**			
Discussion	23a	Provide a general interpretation of the results in the context of other evidence.	Sections 5.1–5.2.
	23b	Discuss any limitations of the evidence included in the review.	Sections 3–4 and Sections 5.1–5.2.

PRISMA 2020 Checklist

Section and Topic	Item #	Checklist item	Location where item is reported
	23c	Discuss any limitations of the review processes used.	Section 5.2, review-process limitations paragraph.
	23d	Discuss implications of the results for practice, policy, and future research.	Section 5.2, research challenges and future outlook.
**OTHER INFORMATION**			
Registration and protocol	24a	Provide registration information for the review, including register name and registration number, or state that the review was not registered.	Section 2.1, paragraph 4: the review was not registered.
	24b	Indicate where the review protocol can be accessed, or state that a protocol was not prepared.	Section 2.1, paragraph 4: no protocol was prepared.
	24c	Describe and explain any amendments to information provided at registration or in the protocol.	Not applicable: the review was not registered and no protocol was prepared; therefore, no registered/protocol amendments occurred.
Support	25	Describe sources of financial or non-financial support for the review, and the role of the funders or sponsors in the review.	Funding statement, including the funder's role.
Competing interests	26	Declare any competing interests of review authors.	Conflicts of Interest statement.
Availability of data, code and other materials	27	Report which of the following are publicly available and where they can be found: template data collection forms; data extracted from included studies; data used for all analyses; analytic code; any other materials used in the review.	No new primary data or meta-analytic code were generated.

From: Page MJ, McKenzie JE, Bossuyt PM, Boutron I, Hoffmann TC, Mulrow CD, et al. The PRISMA 2020 statement: an updated guideline for reporting systematic reviews. BMJ 2021;372:n71. doi: 10.1136/bmj.n71.
This work is licensed under CC BY 4.0. To view a copy of this license, visit https://creativecommons.org/licenses/by/4.0/
