# Supplementary material for: Intelligent Recognition of Building Facade Defects: A Multilevel Review from Visual Perception to Engineering Operations and Maintenance
Source: Sensors (Basel). 2026 Aug 30;26(17):5498. doi: 10.3390/s26175498 (PMC13568442; doi:10.3390/s26175498)
Supplement: Supplementary file 1 [file sensors-26-05498-s001.zip › Supplementary_Table_S1_Complete_Coding_203.pdf]

Supplementary Table S1. Complete Source-Level Coding of the 203 Retained Sources

Sources are listed in the numerical order used in the revised manuscript. The table separates direct facade evidence from transferable, enabling, and contextual evidence before applying the A/T/F/S/M framework. References [204–209], which were added after closure of the screened corpus for supplementary methodological context, are not included in this table or its descriptive counts.

Corpus Summary

Evidence strata: E1=49 (24.1%) direct facade/building-envelope evidence; E2=60 (29.6%) transferable adjacent-domain evidence; E3=65 (32.0%) enabling or non-defect technical sources; E4=29 (14.3%) contextual/synthesis sources.
Quantitative visual-technical subset: n=106. T1=16 (15.1%), T2=16 (15.1%), T3=45 (42.5%), T4=9 (8.5%), T5=20 (18.9%); F0=71 (67.0%), F1=18 (17.0%), F2=6 (5.7%), and F3=11 (10.4%).
Spatial and maturity distributions: S0=15 (14.2%), S1=70 (66.0%), S2=13 (12.3%), S3=8 (7.5%); M1=59 (55.7%), M2=46 (43.4%), M3=1 (0.9%). Eight studies reached T5/S3, and one reached T5/S3/M3.

Coding Rules and Evidence Flags

A0=image-level labels; A1=bounding boxes; A2=pixel masks; A2*=dense point-level labels, treated as the 3D analogue of a pixel mask. T records the highest validated output: T1 classification, T2 detection, T3 segmentation, T4 geometric quantification, and T5 3D localization/reconstruction. F0 denotes no cross-source fusion; F1 input/image-level fusion; F2 learned feature-level interaction; and F3 decision-, temporal-, or result-level combination. S0=no spatial mapping, S1=image coordinates, S2=point cloud/mesh, and S3=BIM/digital twin. M1=proof of concept, M2=controlled field validation, and M3=integrated operational workflow.
Evidence-strength flags: EV=external validation; PD=public data; PC=public code; QGT=quantitative/geometric ground truth; CD=cross-domain testing; FE=field evidence. Y means explicitly verified in the retained source or authoritative record. NR means not reported or not verified from the retained record and must not be interpreted as proof of absence. N/A means not applicable.

Complete Reference-Order Coding Matrix

Ref.	Source (manuscript order)	Evidence	Object / target	Modality	A	T	F	S	M	Evidence-strength flags	Coding note / verification basis
1	Compilation of Bulletins of the First National Comprehensive Natural Disaster Risk Survey: Bulletin No. 6—National Survey of Buildings and Municipal Facilities	E4	Built-environment survey/context	N/A	N/A	N/A	N/A	N/A	N/A	EV:N/A | PD:N/A | PC:N/A | QGT:N/A | CD:N/A | FE:N/A	Enabling or contextual source; included in corpus stratification but excluded from the 106-study visual-technical denominator. Bibliographic record and reported source scope examined.
2	Thirty-Five Percent of Residential Buildings Enter 'Middle Age' as Urban Physical Examinations Confront Housing Safety	E4	Building-stock safety/context	N/A	N/A	N/A	N/A	N/A	N/A	EV:N/A | PD:N/A | PC:N/A | QGT:N/A | CD:N/A | FE:N/A	Enabling or contextual source; included in corpus stratification but excluded from the 106-study visual-technical denominator. Bibliographic record and reported source scope examined.
3	Guiding Opinions on Comprehensively Carrying Out Urban Physical Examinations (Jian Ke [2023] No. 75)	E4	Urban physical-examination policy	N/A	N/A	N/A	N/A	N/A	N/A	EV:N/A | PD:N/A | PC:N/A | QGT:N/A | CD:N/A | FE:N/A	Enabling or contextual source; included in corpus stratification but excluded from the 106-study visual-technical denominator. Bibliographic record and reported source scope examined.
4	Ministry of Housing and Urban-Rural Development: Coordinated Advancement of Urban Physical Examination and Urban Renewal	E4	Urban renewal/context	N/A	N/A	N/A	N/A	N/A	N/A	EV:N/A | PD:N/A | PC:N/A | QGT:N/A | CD:N/A | FE:N/A	Enabling or contextual source; included in corpus stratification but excluded from the 106-study visual-technical denominator. Bibliographic record and reported source scope examined.
5	Notice of the State Council on Issuing the Urban Renewal Plan for the 15th Five-Year Plan Period (Guo Fa [2026] No. 12)	E4	Urban-renewal policy	N/A	N/A	N/A	N/A	N/A	N/A	EV:N/A | PD:N/A | PC:N/A | QGT:N/A | CD:N/A | FE:N/A	Enabling or contextual source; included in corpus stratification but excluded from the 106-study visual-technical denominator. Bibliographic record and reported source scope examined.
6	UAV-Embedded Sensors and Deep Learning for Pathology Identification in Building Façades: A Review	E4	Building facade pathology (review)	UAV imaging and embedded sensors (review)	N/A	N/A	N/A	N/A	N/A	EV:N/A | PD:N/A | PC:N/A | QGT:N/A | CD:N/A | FE:N/A	Enabling or contextual source; included in corpus stratification but excluded from the 106-study visual-technical denominator. Full text examined; coding was limited to explicitly reported information.
7	A Review of Computer Vision-Based Crack Detection Methods in Civil Infrastructure: Progress and Challenges	E4	Civil-infrastructure cracks (review)	RGB/computer vision (review)	N/A	N/A	N/A	N/A	N/A	EV:N/A | PD:N/A | PC:N/A | QGT:N/A | CD:N/A | FE:N/A	Enabling or contextual source; included in corpus stratification but excluded from the 106-study visual-technical denominator. Full text examined; coding was limited to explicitly reported information.
8	UAV-Based Deep Learning Applications for Automated Inspection of Civil Infrastructure	E4	Civil-infrastructure inspection (review)	UAV-based sensing (review)	N/A	N/A	N/A	N/A	N/A	EV:N/A | PD:N/A | PC:N/A | QGT:N/A | CD:N/A | FE:N/A	Enabling or contextual source; included in corpus stratification but excluded from the 106-study visual-technical denominator. Full text examined; coding was limited to explicitly reported information.
9	A Scoping Review: Applications of Deep Learning in Non-Destructive Building Tests	E4	Building NDT (review)	Multiple NDT modalities (review)	N/A	N/A	N/A	N/A	N/A	EV:N/A | PD:N/A | PC:N/A | QGT:N/A | CD:N/A | FE:N/A	Enabling or contextual source; included in corpus stratification but excluded from the 106-study visual-technical denominator. Full text examined; coding was limited to explicitly reported information.

Ref.	Source (manuscript order)	Evidence	Object / target	Modality	A	T	F	S	M	Evidence-strength flags	Coding note / verification basis
10	Bibliometric Analysis and Review of Deep Learning-Based Crack Detection Literature Published between 2010 and 2022	E4	Crack detection (bibliometric review)	RGB/computer vision (review)	N/A	N/A	N/A	N/A	N/A	EV:N/A | PD:N/A | PC:N/A | QGT:N/A | CD:N/A | FE:N/A	Enabling or contextual source; included in corpus stratification but excluded from the 106-study visual-technical denominator. Full text examined; coding was limited to explicitly reported information.
11	A Comprehensive Review of Deep Learning-Based Crack Detection Approaches	E4	Structural crack detection (review)	RGB/computer vision (review)	N/A	N/A	N/A	N/A	N/A	EV:N/A | PD:N/A | PC:N/A | QGT:N/A | CD:N/A | FE:N/A	Enabling or contextual source; included in corpus stratification but excluded from the 106-study visual-technical denominator. Full text examined; coding was limited to explicitly reported information.
12	Deep Learning-Based Crack Segmentation for Civil Infrastructure: Data Types, Architectures, and Benchmarked Performance	E4	Civil-infrastructure crack segmentation (review)	RGB/computer vision (review)	N/A	N/A	N/A	N/A	N/A	EV:N/A | PD:N/A | PC:N/A | QGT:N/A | CD:N/A | FE:N/A	Enabling or contextual source; included in corpus stratification but excluded from the 106-study visual-technical denominator. Full text examined; coding was limited to explicitly reported information.
13	Artificial Intelligence-Enhanced Non-Destructive Defect Detection for Civil Infrastructure	E4	Civil-infrastructure NDT (review)	Multiple NDT modalities (review)	N/A	N/A	N/A	N/A	N/A	EV:N/A | PD:N/A | PC:N/A | QGT:N/A | CD:N/A | FE:N/A	Enabling or contextual source; included in corpus stratification but excluded from the 106-study visual-technical denominator. Full text examined; coding was limited to explicitly reported information.
14	Advances in Artificial Intelligence for Structural Health Monitoring: A Comprehensive Review	E4	Structural health monitoring (review)	Multiple SHM modalities (review)	N/A	N/A	N/A	N/A	N/A	EV:N/A | PD:N/A | PC:N/A | QGT:N/A | CD:N/A | FE:N/A	Enabling or contextual source; included in corpus stratification but excluded from the 106-study visual-technical denominator. Bibliographic record and reported source scope examined.
15	A Review on Computer Vision Based Defect Detection and Condition Assessment of Concrete and Asphalt Civil Infrastructure	E4	Concrete/asphalt defects (review)	RGB/computer vision (review)	N/A	N/A	N/A	N/A	N/A	EV:N/A | PD:N/A | PC:N/A | QGT:N/A | CD:N/A | FE:N/A	Enabling or contextual source; included in corpus stratification but excluded from the 106-study visual-technical denominator. Full text examined; coding was limited to explicitly reported information.
16	Advances in Computer Vision-Based Civil Infrastructure Inspection and Monitoring	E4	Civil-infrastructure inspection and monitoring (review)	RGB/computer vision (review)	N/A	N/A	N/A	N/A	N/A	EV:N/A | PD:N/A | PC:N/A | QGT:N/A | CD:N/A | FE:N/A	Enabling or contextual source; included in corpus stratification but excluded from the 106-study visual-technical denominator. Full text examined; coding was limited to explicitly reported information.
17	The PRISMA 2020 Statement: An Updated Guideline for Reporting Systematic Reviews	E4	Systematic-review reporting guideline	N/A	N/A	N/A	N/A	N/A	N/A	EV:N/A | PD:N/A | PC:N/A | QGT:N/A | CD:N/A | FE:N/A	Enabling or contextual source; included in corpus stratification but excluded from the 106-study visual-technical denominator. Full text examined; coding was limited to explicitly reported information.
18	PRISMA-S: An Extension to the PRISMA Statement for Reporting Literature Searches in Systematic Reviews	E4	Literature-search reporting guideline	N/A	N/A	N/A	N/A	N/A	N/A	EV:N/A | PD:N/A | PC:N/A | QGT:N/A | CD:N/A | FE:N/A	Enabling or contextual source; included in corpus stratification but excluded from the 106-study visual-technical denominator. Bibliographic record and reported source scope examined.
19	Deep Learning-Based Crack Damage Detection Using Convolutional Neural Networks	E2	General/other; crack	RGB	A0	T2	F0	S1	M2	EV:Y | PD:NR | PC:NR | QGT:NR | CD:Y | FE:Y	Full text examined; coding was limited to explicitly reported information.
20	SDNET2018: An Annotated Image Dataset for Non-Contact Concrete Crack Detection Using Deep Convolutional Neural Networks	E2	General structure/concrete; crack	RGB	A0	T1	F0	S0	M1	EV:NR | PD:Y | PC:NR | QGT:NR | CD:NR | FE:NR	Full text examined; coding was limited to explicitly reported information.
21	Meta-Learning Convolutional Neural Architectures for Multi-Target Concrete Defect Classification with the COncrete DEfect BRidge IMage Dataset	E2	Bridge/deck; multi-defect/damage	RGB	A0	T1	F0	S0	M1	EV:NR | PD:Y | PC:NR | QGT:NR | CD:NR | FE:NR	Full text examined; coding was limited to explicitly reported information.
22	BFD-YOLO: A YOLOv7-Based Detection Method for Building Façade Defects	E1	Building façade/envelope; multi-defect/damage	RGB	A1	T2	F0	S1	M1	EV:NR | PD:NR | PC:NR | QGT:NR | CD:NR | FE:NR	Full text examined; coding was limited to explicitly reported information.
23	Defects Inspection System for Building Facades Using Drones and Deep Learning Method	E1	Building façade/envelope; multi-defect/damage	UAV RGB	A2	T3	F0	S1	M2	EV:Y | PD:NR | PC:NR | QGT:NR | CD:Y | FE:Y	Full text examined; coding was limited to explicitly reported information.
24	Intelligent Detection Method of Defects in High-Rise Building Facades Using Infrared Thermography	E1	Building façade/envelope; multi-defect/damage	IRT	A1	T2	F0	S1	M2	EV:NR | PD:NR | PC:NR | QGT:NR | CD:NR | FE:Y	Full text examined; coding was limited to explicitly reported information.
25	Fusion-Based Damage Segmentation for Multimodal Building Façade Images from an End-to-End Perspective	E1	Building façade/envelope; multi-defect/damage	RGB+IRT	A2	T3	F1	S1	M2	EV:NR | PD:NR | PC:NR | QGT:NR | CD:NR | FE:Y	Full text examined; coding was limited to explicitly reported information.
26	Weakly-Aligned Cross-Modal Learning Framework for Subsurface Defect Segmentation on Building Façades Using UAVs	E1	Building façade/envelope; multi-defect/damage	RGB+IRT	A2	T3	F2	S1	M2	EV:NR | PD:NR | PC:NR | QGT:NR | CD:NR | FE:Y	Full text examined; coding was limited to explicitly reported information.
27	SCSegamba: Lightweight Structure-Aware Vision Mamba for Crack Segmentation in Structures	E2	General/other; crack	RGB	A2	T3	F0	S1	M1	EV:NR | PD:Y | PC:Y | QGT:NR | CD:NR | FE:NR	Full text examined; coding was limited to explicitly reported information.

Ref.	Source (manuscript order)	Evidence	Object / target	Modality	A	T	F	S	M	Evidence-strength flags	Coding note / verification basis
28	Surface Defect-Extended BIM Generation Leveraging UAV Images and Deep Learning	E1	General/other; multi-defect/damage	UAV RGB	A2	T5	F1	S3	M2	EV:NR | PD:NR | PC:NR | QGT:Y | CD:NR | FE:Y	Full text examined; coding was limited to explicitly reported information.
29	Damage-Augmented Digital Twins towards the Automated Inspection of Buildings	E1	Building; multi-defect/damage	RGB	A2	T5	F1	S2	M2	EV:NR | PD:Y | PC:Y | QGT:NR | CD:NR | FE:Y	Full text examined; coding was limited to explicitly reported information.
30	Simultaneous Digital Twin: Chaining Climbing-Robot, Defect Segmentation, and Model Updating for Building Facade Inspection	E1	Building façade/envelope; multi-defect/damage	RGB	A2	T5	F3	S3	M3	EV:NR | PD:NR | PC:NR | QGT:Y | CD:NR | FE:Y	Full text examined; coding was limited to explicitly reported information.
31	UAV and Deep Learning for Automated Detection and Visualization of Façade Defects in Existing Residential Buildings	E1	Building façade/envelope; multi-defect/damage	RGB+IRT+3D	A2	T5	F3	S2	M2	EV:NR | PD:NR | PC:NR | QGT:NR | CD:NR | FE:Y	Full text examined; coding was limited to explicitly reported information.
32	Crack Detection Using Enhanced Thresholding on UAV-Based Collected Images	E1	Building façade/envelope; crack	UAV RGB	A2	T3	F0	S1	M2	EV:NR | PD:NR | PC:NR | QGT:NR | CD:NR | FE:Y	Validated on UAV images of commercial-building cracks. Author preprint or abstract examined; coding was limited to explicitly reported information.
33	DAMAGE DETECTION ON BUILDING FAÇADES USING MULTI-TEMPORAL AERIAL OBLIQUE IMAGERY	E1	Building façade/envelope; multi-defect/damage	UAV RGB	A0	T1	F1	S0	M2	EV:NR | PD:NR | PC:NR | QGT:NR | CD:NR | FE:Y	Full text examined; coding was limited to explicitly reported information.
34	Building Facade Debonding Damage Detection Method Based on Explainable Artificial Intelligence and UAV-Thermography in Cross Scale Field of View	E1	Building façade/envelope; delamination/debonding	IRT	A1	T2	F0	S1	M2	EV:NR | PD:NR | PC:NR | QGT:NR | CD:NR | FE:Y	Full text examined; coding was limited to explicitly reported information.
35	Enhancing Bridge Deck Delamination Detection Based on Aerial Thermography through Grayscale Morphologic Reconstruction: A Case Study	E2	Bridge/deck; delamination/debonding	IRT	A2	T4	F0	S1	M2	EV:NR | PD:NR | PC:NR | QGT:Y | CD:NR | FE:Y	Delamination area was quantified and compared with hammer-sounding ground truth. Author preprint or abstract examined; coding was limited to explicitly reported information.
36	Parsing Facades with Shape Grammars and Reinforcement Learning	E3	Building façade/envelope; N/A or non-defect target	RGB	N/A	N/A	N/A	N/A	N/A	EV:N/A | PD:N/A | PC:N/A | QGT:N/A | CD:N/A | FE:N/A	Enabling or contextual source; included in corpus stratification but excluded from the 106-study visual-technical denominator. Full text examined; coding was limited to explicitly reported information.
37	Spatial Pattern Templates for Recognition of Objects with Regular Structure	E3	General/other; N/A or non-defect target	RGB	N/A	N/A	N/A	N/A	N/A	EV:N/A | PD:N/A | PC:N/A | QGT:N/A | CD:N/A | FE:N/A	Enabling or contextual source; included in corpus stratification but excluded from the 106-study visual-technical denominator. Full text examined; coding was limited to explicitly reported information.
38	A Three-Layered Approach to Facade Parsing	E3	Building façade/envelope; N/A or non-defect target	RGB	N/A	N/A	N/A	N/A	N/A	EV:N/A | PD:N/A | PC:N/A | QGT:N/A | CD:N/A | FE:N/A	Enabling or contextual source; included in corpus stratification but excluded from the 106-study visual-technical denominator. Full text examined; coding was limited to explicitly reported information.
39	Wall Crack Multiclass Classification: Expertise-Based Dataset Construction and Learning Algorithms Performance Comparison	E1	General/other; crack	RGB	A0	T1	F0	S0	M1	EV:NR | PD:NR | PC:NR | QGT:NR | CD:NR | FE:NR	Full text examined; coding was limited to explicitly reported information.
40	High-Resolution Infrastructure Defect Detection Dataset Sourced by Unmanned Systems and Validated with Deep Learning	E2	General structure/concrete; multi-defect/damage	RGB	A1	T2	F0	S1	M1	EV:Y | PD:Y | PC:NR | QGT:NR | CD:Y | FE:NR	Full text examined; coding was limited to explicitly reported information.
41	A Dataset of Building Surface Defects Collected by UAVs for Machine Learning-Based Detection	E1	Building façade/envelope; multi-defect/damage	UAV RGB	A1	T2	F0	S1	M1	EV:NR | PD:Y | PC:NR | QGT:NR | CD:NR | FE:NR	Full text examined; coding was limited to explicitly reported information.
42	Automatic Image-Based Brick Segmentation and Crack Detection of Masonry Walls Using Machine Learning	E1	Masonry/cladding; crack	RGB	A2	T4	F0	S1	M1	EV:NR | PD:NR | PC:NR | QGT:Y | CD:NR | FE:NR	Full text examined; coding was limited to explicitly reported information.
43	Building Facade Evaluation Using Instance Segmentation on Thermal Images	E1	Building façade/envelope; thermal/moisture anomaly	IRT	A2	T3	F0	S1	M1	EV:NR | PD:Y | PC:NR | QGT:NR | CD:NR | FE:NR	Full text examined; coding was limited to explicitly reported information.
44	Building Envelope Thermal Anomaly Detection Using an Integrated Vision-Based Technique and Semantic Segmentation	E1	Building façade/envelope; thermal/moisture anomaly	RGB+IRT	A2	T3	F3	S1	M2	EV:NR | PD:NR | PC:NR | QGT:NR | CD:NR | FE:Y	Full text examined; coding was limited to explicitly reported information.
45	Deep Learning for Crack Detection on Masonry Façades Using Limited Data and Transfer Learning	E1	Masonry/cladding; crack	RGB	A0	T1	F0	S0	M1	EV:NR | PD:NR | PC:NR | QGT:NR | CD:NR | FE:NR	Full text examined; coding was limited to explicitly reported information.
46	One-Class Anomaly Detection through Color-to-Thermal AI for Building Envelope Inspection	E1	Building façade/envelope; thermal/moisture anomaly	RGB+IRT	A2	T3	F3	S1	M1	EV:NR | PD:NR | PC:NR | QGT:NR | CD:NR | FE:NR	Full text examined; coding was limited to explicitly reported information.
47	Cross-Dataset Evaluation of Deep Learning Models for Crack Classification in Structural Surfaces	E2	General structure/concrete; crack	RGB	A0	T1	F0	S0	M1	EV:Y | PD:Y | PC:NR | QGT:NR | CD:Y | FE:NR	Full text examined; coding was limited to explicitly reported information.

Ref.	Source (manuscript order)	Evidence	Object / target	Modality	A	T	F	S	M	Evidence-strength flags	Coding note / verification basis
48	Tile Delamination on Façades of Tall Buildings	E1	Building façade/envelope; delamination/debonding	RGB	N/A	N/A	N/A	N/A	N/A	EV:NR | PD:NR | PC:NR | QGT:NR | CD:NR | FE:Y	Physical/mechanistic façade evidence; excluded from A/T/F/S/M distributions. Full text examined; coding was limited to explicitly reported information.
49	A Comprehensive Review of Aluminium Composite Panels: Current Research, Challenges, and Future Research Direction	E4	Facade material systems (review)	N/A	N/A	N/A	N/A	N/A	N/A	EV:N/A | PD:N/A | PC:N/A | QGT:N/A | CD:N/A | FE:N/A	Enabling or contextual source; included in corpus stratification but excluded from the 106-study visual-technical denominator. Full text examined; coding was limited to explicitly reported information.
50	Building Façade Datasets for Analyzing Building Characteristics Using Deep Learning	E3	Building façade/envelope; N/A or non-defect target	RGB	N/A	N/A	N/A	N/A	N/A	EV:N/A | PD:N/A | PC:N/A | QGT:N/A | CD:N/A | FE:N/A	Enabling or contextual source; included in corpus stratification but excluded from the 106-study visual-technical denominator. Full text examined; coding was limited to explicitly reported information.
51	Crack Detection and Segmentation Using Deep Learning with 3D Reality Mesh Model for Quantitative Assessment and Integrated Visualization	E2	General/other; crack	RGB+SfM/MVS	A2	T5	F1	S2	M2	EV:Y | PD:NR | PC:NR | QGT:Y | CD:Y | FE:Y	Full text examined; coding was limited to explicitly reported information.
52	PointCrack3D: Crack Detection in Unstructured Environments Using a 3D-Point-Cloud-Based Deep Neural Network	E2	General/other; crack	colored LiDAR point cloud	A2*	T5	F0	S2	M2	EV:Y | PD:NR | PC:NR | QGT:Y | CD:Y | FE:Y	A2* denotes dense point-level labels (3D analogue of a pixel mask). Full text examined; coding was limited to explicitly reported information.
53	CrackTree: Automatic Crack Detection from Pavement Images	E2	Road/pavement; crack	RGB	A2	T3	F0	S1	M1	EV:NR | PD:NR | PC:NR | QGT:NR | CD:NR | FE:NR	Full text examined; coding was limited to explicitly reported information.
54	Automatic Road Crack Detection Using Random Structured Forests	E2	Road/pavement; crack	RGB	A2	T3	F0	S1	M1	EV:NR | PD:NR | PC:NR | QGT:NR | CD:NR | FE:NR	Full text examined; coding was limited to explicitly reported information.
55	Feature Pyramid and Hierarchical Boosting Network for Pavement Crack Detection	E2	Road/pavement; crack	RGB	A2	T3	F0	S1	M1	EV:NR | PD:NR | PC:NR | QGT:NR | CD:NR | FE:NR	Full text examined; coding was limited to explicitly reported information.
56	DeepCrack: A Deep Hierarchical Feature Learning Architecture for Crack Segmentation	E2	General/other; crack	RGB	A2	T3	F0	S1	M1	EV:NR | PD:NR | PC:NR | QGT:NR | CD:NR | FE:NR	Full text examined; coding was limited to explicitly reported information.
57	CrackSeg9k: A Collection and Benchmark for Crack Segmentation Datasets and Frameworks	E2	General/other; crack	RGB	A2	T3	F0	S1	M1	EV:NR | PD:Y | PC:NR | QGT:NR | CD:NR | FE:NR	Full text examined; coding was limited to explicitly reported information.
58	StructDamage: A Large-Scale Unified Crack and Surface Defect Dataset for Robust Structural Damage Detection	E2	General structure/concrete; crack	RGB	A0	T1	F0	S0	M1	EV:NR | PD:Y | PC:NR | QGT:NR | CD:NR | FE:NR	Full text examined; coding was limited to explicitly reported information.
59	Decoding Building Facades: Automated Element and Material Recognition in Street-Level Images for Large-Scale Building Stock Assessments	E3	Building façade/envelope; N/A or non-defect target	RGB	N/A	N/A	N/A	N/A	N/A	EV:N/A | PD:N/A | PC:N/A | QGT:N/A | CD:N/A | FE:N/A	Enabling or contextual source; included in corpus stratification but excluded from the 106-study visual-technical denominator. Full text examined; coding was limited to explicitly reported information.
60	Image-Based Concrete Crack Detection Using Convolutional Neural Network and Exhaustive Search Technique	E2	General structure/concrete; crack	RGB	A0	T2	F0	S1	M1	EV:NR | PD:NR | PC:NR | QGT:NR | CD:NR | FE:NR	Full text examined; coding was limited to explicitly reported information.
61	Crack and Noncrack Classification from Concrete Surface Images Using Machine Learning	E2	General structure/concrete; crack	RGB	A0	T1	F0	S0	M1	EV:NR | PD:NR | PC:NR | QGT:NR | CD:NR | FE:NR	Full text examined; coding was limited to explicitly reported information.
62	An Intelligent Classification Model for Surface Defects on Cement Concrete Bridges	E2	Bridge/deck; multi-defect/damage	RGB	A0	T1	F0	S0	M1	EV:NR | PD:NR | PC:NR | QGT:NR | CD:NR | FE:NR	Full text examined; coding was limited to explicitly reported information.
63	Deep Convolutional Neural Networks with Transfer Learning for Computer Vision-Based Data-Driven Pavement Distress Detection	E2	Road/pavement; N/A or non-defect target	RGB	A0	T1	F0	S0	M1	EV:NR | PD:NR | PC:NR | QGT:NR | CD:NR | FE:NR	Full text examined; coding was limited to explicitly reported information.
64	Road Crack Detection Using Deep Convolutional Neural Network	E2	Road/pavement; crack	RGB	A0	T2	F0	S1	M1	EV:NR | PD:NR | PC:NR | QGT:NR | CD:NR | FE:NR	Full text examined; coding was limited to explicitly reported information.
65	Intelligent Detection of Building Cracks Based on Deep Learning	E2	Building; crack	RGB	A2	T3	F0	S1	M1	EV:NR | PD:NR | PC:NR | QGT:NR | CD:NR | FE:NR	Full text examined; coding was limited to explicitly reported information.
66	Building Surface Crack Detection Using Deep Learning Technology	E1	Building façade/envelope; crack	RGB	A0	T1	F0	S0	M1	EV:NR | PD:NR | PC:NR | QGT:NR | CD:NR | FE:NR	Full text examined; coding was limited to explicitly reported information.
67	Ensembles of Deep Neural Networks for the Automatic Detection of Building Facade Defects From Images	E1	Building façade/envelope; multi-defect/damage	RGB	A0	T1	F3	S0	M1	EV:NR | PD:Y | PC:NR | QGT:NR | CD:NR | FE:NR	Full text examined; coding was limited to explicitly reported information.
68	Disease Detection on Exterior Surfaces of Buildings Using Deep Learning in China	E1	Building; multi-defect/damage	RGB	A1	T2	F0	S1	M2	EV:NR | PD:NR | PC:NR | QGT:NR | CD:NR | FE:Y	Full text examined; coding was limited to explicitly reported information.
69	Application of Deep Learning and Unmanned Aerial Vehicle on Building Maintenance	E1	Building; N/A or non-defect target	UAV RGB	A0	T2	F0	S1	M2	EV:NR | PD:NR | PC:NR | QGT:NR | CD:NR | FE:Y	Full text examined; coding was limited to explicitly reported information.

Ref.	Source (manuscript order)	Evidence	Object / target	Modality	A	T	F	S	M	Evidence-strength flags	Coding note / verification basis
70	Transfer Learned Deep Feature Based Crack Detection Using Support Vector Machine: A Comparative Study	E2	General/other; crack	RGB	A0	T1	F0	S0	M1	EV:Y | PD:Y | PC:NR | QGT:NR | CD:Y | FE:NR	Full text examined; coding was limited to explicitly reported information.
71	Faster R-CNN: Towards Real-Time Object Detection with Region Proposal Networks	E3	General/other; N/A or non-defect target	RGB	N/A	N/A	N/A	N/A	N/A	EV:N/A | PD:N/A | PC:N/A | QGT:N/A | CD:N/A | FE:N/A	Enabling or contextual source; included in corpus stratification but excluded from the 106-study visual-technical denominator. Bibliographic record and reported source scope examined.
72	You Only Look Once: Unified, Real-Time Object Detection	E3	General/other; N/A or non-defect target	RGB	N/A	N/A	N/A	N/A	N/A	EV:N/A | PD:N/A | PC:N/A | QGT:N/A | CD:N/A | FE:N/A	Enabling or contextual source; included in corpus stratification but excluded from the 106-study visual-technical denominator. Full text examined; coding was limited to explicitly reported information.
73	YOLOv3: An Incremental Improvement	E3	General/other; N/A or non-defect target	RGB	N/A	N/A	N/A	N/A	N/A	EV:N/A | PD:N/A | PC:N/A | QGT:N/A | CD:N/A | FE:N/A	Enabling or contextual source; included in corpus stratification but excluded from the 106-study visual-technical denominator. Full text examined; coding was limited to explicitly reported information.
74	YOLOv4: Optimal Speed and Accuracy of Object Detection	E3	General/other; N/A or non-defect target	RGB	N/A	N/A	N/A	N/A	N/A	EV:N/A | PD:N/A | PC:N/A | QGT:N/A | CD:N/A | FE:N/A	Enabling or contextual source; included in corpus stratification but excluded from the 106-study visual-technical denominator. Full text examined; coding was limited to explicitly reported information.
75	Autonomous Structural Visual Inspection Using Region-Based Deep Learning for Detecting Multiple Damage Types	E2	General structure/concrete; multi-defect/damage	RGB	A1	T2	F0	S1	M2	EV:NR | PD:NR | PC:NR | QGT:NR | CD:NR | FE:Y	Full text examined; coding was limited to explicitly reported information.
76	Vision-Based Automated Crack Detection for Bridge Inspection	E2	Bridge/deck; crack	RGB	A1	T2	F0	S1	M2	EV:NR | PD:NR | PC:NR | QGT:NR | CD:NR | FE:Y	Full text examined; coding was limited to explicitly reported information.
77	NB-CNN: Deep Learning-Based Crack Detection Using Convolutional Neural Network and Naïve Bayes Data Fusion	E2	General/other; crack	RGB video	A0	T2	F3	S1	M2	EV:NR | PD:NR | PC:NR | QGT:NR | CD:NR | FE:Y	Full text examined; coding was limited to explicitly reported information.
78	An Unmanned Aerial Vehicle Implemented Network for Real-time Crack Detection in Stone Cladding	E1	Masonry/cladding; crack	stereo RGB+3D model	A2	T5	F1	S2	M2	EV:NR | PD:NR | PC:NR | QGT:Y | CD:NR | FE:Y	Full text examined; coding was limited to explicitly reported information.
79	Automated Defect Detection and Classification in Ashlar Masonry Walls Using Machine Learning	E1	Masonry/cladding; multi-defect/damage	RGB	A0	T1	F0	S1	M2	EV:NR | PD:NR | PC:NR | QGT:NR | CD:NR | FE:Y	Full text examined; coding was limited to explicitly reported information.
80	A Deep Learning Framework for Real-Time Multi-Task Recognition and Measurement of Concrete Cracks	E2	General structure/concrete; crack	RGB	A2	T4	F0	S1	M1	EV:NR | PD:NR | PC:NR | QGT:Y | CD:NR | FE:NR	Full text examined; coding was limited to explicitly reported information.
81	Deep Learning Algorithm for Real-Time Automatic Crack Detection, Segmentation, Qualification	E2	General/other; crack	RGB	A2	T4	F0	S1	M1	EV:NR | PD:NR | PC:NR | QGT:Y | CD:NR | FE:NR	Full text examined; coding was limited to explicitly reported information.
82	DAH-YOLO: An Accurate and Efficient Model for Crack Detection in Complex Scenarios	E2	General/other; crack	RGB	A1	T2	F0	S1	M1	EV:Y | PD:Y | PC:NR | QGT:NR | CD:Y | FE:NR	Full text examined; coding was limited to explicitly reported information.
83	Advanced Metaheuristic Optimization with Enhanced Dung Beetle Algorithm for Automated Crack Detection in Civil Infrastructure	E2	General structure/concrete; crack	RGB	A0	T1	F0	S0	M1	EV:Y | PD:Y | PC:NR | QGT:NR | CD:Y | FE:NR	Full text examined; coding was limited to explicitly reported information.
84	Automated Post-Earthquake Structural Damage Assessment of Concrete Buildings Using a Hybrid Deep Learning and Rule-Based Framework on Image Datasets	E2	General structure/concrete; multi-defect/damage	RGB	A1	T2	F3	S1	M2	EV:Y | PD:NR | PC:NR | QGT:NR | CD:Y | FE:Y	Full text examined; coding was limited to explicitly reported information.
85	Automatic Pavement Crack Detection Based on Structured Prediction with the Convolutional Neural Network	E2	Road/pavement; crack	RGB	A2	T3	F0	S1	M1	EV:NR | PD:NR | PC:NR | QGT:NR | CD:NR | FE:NR	Full text examined; coding was limited to explicitly reported information.
86	Fully Convolutional Networks for Semantic Segmentation	E3	General/other; N/A or non-defect target	RGB	N/A	N/A	N/A	N/A	N/A	EV:N/A | PD:N/A | PC:N/A | QGT:N/A | CD:N/A | FE:N/A	Enabling or contextual source; included in corpus stratification but excluded from the 106-study visual-technical denominator. Full text examined; coding was limited to explicitly reported information.
87	U-Net: Convolutional Networks for Biomedical Image Segmentation	E3	General/other; N/A or non-defect target	RGB	N/A	N/A	N/A	N/A	N/A	EV:N/A | PD:N/A | PC:N/A | QGT:N/A | CD:N/A | FE:N/A	Enabling or contextual source; included in corpus stratification but excluded from the 106-study visual-technical denominator. Full text examined; coding was limited to explicitly reported information.
88	SegNet: A Deep Convolutional Encoder-Decoder Architecture for Image Segmentation	E3	General/other; N/A or non-defect target	RGB	N/A	N/A	N/A	N/A	N/A	EV:N/A | PD:N/A | PC:N/A | QGT:N/A | CD:N/A | FE:N/A	Enabling or contextual source; included in corpus stratification but excluded from the 106-study visual-technical denominator. Full text examined; coding was limited to explicitly reported information.

Ref.	Source (manuscript order)	Evidence	Object / target	Modality	A	T	F	S	M	Evidence-strength flags	Coding note / verification basis
89	DeepLab: Semantic Image Segmentation with Deep Convolutional Nets, Atrous Convolution, and Fully Connected CRFs	E3	General/other; N/A or non-defect target	RGB	N/A	N/A	N/A	N/A	N/A	EV:N/A | PD:N/A | PC:N/A | QGT:N/A | CD:N/A | FE:N/A	Enabling or contextual source; included in corpus stratification but excluded from the 106-study visual-technical denominator. Full text examined; coding was limited to explicitly reported information.
90	Encoder-Decoder with Atrous Separable Convolution for Semantic Image Segmentation	E3	General/other; N/A or non-defect target	RGB	N/A	N/A	N/A	N/A	N/A	EV:N/A | PD:N/A | PC:N/A | QGT:N/A | CD:N/A | FE:N/A	Enabling or contextual source; included in corpus stratification but excluded from the 106-study visual-technical denominator. Full text examined; coding was limited to explicitly reported information.
91	Mask R-CNN	E3	General/other; N/A or non-defect target	RGB	N/A	N/A	N/A	N/A	N/A	EV:N/A | PD:N/A | PC:N/A | QGT:N/A | CD:N/A | FE:N/A	Enabling or contextual source; included in corpus stratification but excluded from the 106-study visual-technical denominator. Full text examined; coding was limited to explicitly reported information.
92	Automatic Pixel-Level Crack Detection and Measurement Using Fully Convolutional Network	E2	General/other; crack	RGB	A2	T4	F0	S1	M1	EV:NR | PD:NR | PC:NR | QGT:Y | CD:NR | FE:NR	Full text examined; coding was limited to explicitly reported information.
93	DeepCrack: Learning Hierarchical Convolutional Features for Crack Detection	E2	General/other; crack	RGB	A2	T3	F0	S1	M1	EV:NR | PD:NR | PC:NR | QGT:NR | CD:NR | FE:NR	Full text examined; coding was limited to explicitly reported information.
94	Automated Pixel-Level Pavement Crack Detection on 3D Asphalt Surfaces with a Recurrent Neural Network	E2	Road/pavement; crack	3D/point cloud	A2	T3	F0	S1	M1	EV:NR | PD:NR | PC:NR | QGT:NR | CD:NR | FE:NR	Full text examined; coding was limited to explicitly reported information.
95	Deep Learning–Based Fully Automated Pavement Crack Detection on 3D Asphalt Surfaces with an Improved CrackNet	E2	Road/pavement; crack	3D/point cloud	A2	T3	F0	S1	M1	EV:NR | PD:NR | PC:NR | QGT:NR | CD:NR | FE:NR	Full text examined; coding was limited to explicitly reported information.
96	CrackU-net: A Novel Deep Convolutional Neural Network for Pixelwise Pavement Crack Detection	E2	Road/pavement; crack	RGB	A2	T3	F0	S1	M1	EV:NR | PD:NR | PC:NR | QGT:NR | CD:NR | FE:NR	Full text examined; coding was limited to explicitly reported information.
97	Automatic Segmentation and Quantification of Global Cracks in Concrete Structures Based on Deep Learning	E2	General structure/concrete; crack	RGB	A2	T4	F0	S1	M1	EV:NR | PD:NR | PC:NR | QGT:Y | CD:NR | FE:NR	Full text examined; coding was limited to explicitly reported information.
98	Enhanced Concrete Crack Segmentation with MSMC-U-Net: Integrating Multiscale Features and Contextual Analysis for Infrastructure Safety	E2	General structure/concrete; crack	RGB	A2	T3	F0	S1	M1	EV:NR | PD:NR | PC:NR | QGT:NR | CD:NR | FE:NR	Full text examined; coding was limited to explicitly reported information.
99	Deeper Networks for Pavement Crack Detection	E2	Road/pavement; crack	RGB	A2	T3	F0	S1	M1	EV:NR | PD:NR | PC:NR | QGT:NR | CD:NR | FE:NR	Full text examined; coding was limited to explicitly reported information.
100	Segmentation of Surface Cracks Based on a Fully Convolutional Neural Network and Gated Scale Pooling	E2	General/other; crack	RGB	A2	T3	F0	S1	M1	EV:NR | PD:NR | PC:NR | QGT:NR | CD:NR | FE:NR	Full text examined; coding was limited to explicitly reported information.
101	Dual Flow Fusion Model for Concrete Surface Crack Segmentation	E2	General structure/concrete; crack	RGB	A2	T3	F0	S1	M1	EV:NR | PD:NR | PC:NR | QGT:NR | CD:NR | FE:NR	Full text examined; coding was limited to explicitly reported information.
102	Comparison of Deep Convolutional Neural Networks and Edge Detectors for Image-Based Crack Detection in Concrete	E2	General structure/concrete; crack	RGB	A2	T3	F0	S1	M1	EV:NR | PD:NR | PC:NR | QGT:NR | CD:NR | FE:NR	Bibliographic record and reported source scope examined.
103	Automated Crack Segmentation in Close-Range Building Façade Inspection Images Using Deep Learning Techniques	E1	Building façade/envelope; crack	RGB	A2	T3	F0	S1	M2	EV:NR | PD:NR | PC:NR | QGT:NR | CD:NR | FE:Y	Full text examined; coding was limited to explicitly reported information.
104	Assessment of Geometric Parameters of Segmented Crack on Concrete Building Facade Using Deep Learning	E1	Building façade/envelope; crack	RGB	A2	T4	F0	S1	M2	EV:NR | PD:NR | PC:NR | QGT:Y | CD:NR | FE:Y	Full text examined; coding was limited to explicitly reported information.
105	A Novel Multi-Scale Feature Fusion Network for Tile Spalling Segmentation in Building Exterior	E1	Building façade/envelope; spalling	RGB	A2	T3	F0	S1	M1	EV:NR | PD:NR | PC:NR | QGT:NR | CD:NR | FE:NR	Full text examined; coding was limited to explicitly reported information.
106	A Robust Self-Supervised Approach for Fine-Grained Crack Detection in Concrete Structures	E2	General structure/concrete; crack	RGB	A2	T3	F0	S1	M1	EV:NR | PD:NR | PC:NR | QGT:NR | CD:NR | FE:NR	Full text examined; coding was limited to explicitly reported information.
107	Co-CrackSegment: A New Collaborative Deep Learning Framework for Pixel-Level Semantic Segmentation of Concrete Cracks	E2	General structure/concrete; crack	RGB	A2	T3	F0	S1	M1	EV:NR | PD:NR | PC:NR | QGT:NR | CD:NR | FE:NR	Full text examined; coding was limited to explicitly reported information.
108	An Image Is Worth 16x16 Words: Transformers for Image Recognition at Scale	E3	General/other; N/A or non-defect target	RGB	N/A	N/A	N/A	N/A	N/A	EV:N/A | PD:N/A | PC:N/A | QGT:N/A | CD:N/A | FE:N/A	Enabling or contextual source; included in corpus stratification but excluded from the 106-study visual-technical denominator. Bibliographic record and reported source scope examined.
109	Swin Transformer: Hierarchical Vision Transformer Using Shifted Windows	E3	General/other; N/A or non-defect target	RGB	N/A	N/A	N/A	N/A	N/A	EV:N/A | PD:N/A | PC:N/A | QGT:N/A | CD:N/A | FE:N/A	Enabling or contextual source; included in corpus stratification but excluded from the 106-study visual-technical denominator. Full text examined; coding was limited to explicitly reported information.

Ref.	Source (manuscript order)	Evidence	Object / target	Modality	A	T	F	S	M	Evidence-strength flags	Coding note / verification basis
110	SegFormer: Simple and Efficient Design for Semantic Segmentation with Transformers	E3	General/other; N/A or non-defect target	RGB	N/A	N/A	N/A	N/A	N/A	EV:N/A | PD:N/A | PC:N/A | QGT:N/A | CD:N/A | FE:N/A	Enabling or contextual source; included in corpus stratification but excluded from the 106-study visual-technical denominator. Full text examined; coding was limited to explicitly reported information.
111	End-to-End Object Detection with Transformers	E3	General/other; N/A or non-defect target	RGB	N/A	N/A	N/A	N/A	N/A	EV:N/A | PD:N/A | PC:N/A | QGT:N/A | CD:N/A | FE:N/A	Enabling or contextual source; included in corpus stratification but excluded from the 106-study visual-technical denominator. Full text examined; coding was limited to explicitly reported information.
112	TransUNet: Transformers Make Strong Encoders for Medical Image Segmentation	E3	General/other; N/A or non-defect target	RGB	N/A	N/A	N/A	N/A	N/A	EV:N/A | PD:N/A | PC:N/A | QGT:N/A | CD:N/A | FE:N/A	Enabling or contextual source; included in corpus stratification but excluded from the 106-study visual-technical denominator. Full text examined; coding was limited to explicitly reported information.
113	CA-SegFormer: An Improved Coordinate Attention-Enhanced Model for Crack Segmentation in Concrete Structures	E2	General structure/concrete; crack	RGB	A2	T3	F0	S1	M1	EV:NR | PD:NR | PC:NR | QGT:NR | CD:NR | FE:NR	Full text examined; coding was limited to explicitly reported information.
114	Multi-Temporal Crack Segmentation in Concrete Structures Using Deep Learning Approaches	E2	General structure/concrete; crack	RGB	A2	T3	F1	S1	M1	EV:NR | PD:NR | PC:NR | QGT:NR | CD:NR | FE:NR	Full text examined; coding was limited to explicitly reported information.
115	Segment Anything	E3	General/other; N/A or non-defect target	RGB	N/A	N/A	N/A	N/A	N/A	EV:N/A | PD:N/A | PC:N/A | QGT:N/A | CD:N/A | FE:N/A	Enabling or contextual source; included in corpus stratification but excluded from the 106-study visual-technical denominator. Bibliographic record and reported source scope examined.
116	Fine-Tuning Vision Foundation Model for Crack Segmentation in Civil Infrastructures	E2	General structure/concrete; crack	RGB	A2	T3	F0	S1	M1	EV:NR | PD:NR | PC:NR | QGT:NR | CD:NR | FE:NR	Full text examined; coding was limited to explicitly reported information.
117	Semi-Supervised Crack Detection Using Segment Anything Model and Deep Transfer Learning	E2	General/other; crack	RGB	A2	T3	F0	S1	M1	EV:NR | PD:NR | PC:NR | QGT:NR | CD:NR | FE:NR	Full text examined; coding was limited to explicitly reported information.
118	Segment Any Crack: Deep Semantic Segmentation Adaptation for Crack Detection	E2	General/other; crack	RGB	A2	T3	F0	S1	M1	EV:NR | PD:NR | PC:NR | QGT:NR | CD:NR | FE:NR	Full text examined; coding was limited to explicitly reported information.
119	Application of Segment Anything Model for Civil Infrastructure Defect Assessment	E2	General structure/concrete; multi-defect/damage	RGB	A2	T3	F0	S1	M1	EV:NR | PD:NR | PC:NR | QGT:NR | CD:NR | FE:NR	Full text examined; coding was limited to explicitly reported information.
120	A Two-Stage Concrete Crack Segmentation Method Based on the Improved YOLOv11 and Segment Anything Model	E2	General structure/concrete; crack	RGB	A2	T3	F0	S1	M1	EV:NR | PD:NR | PC:NR | QGT:NR | CD:NR | FE:NR	Full text examined; coding was limited to explicitly reported information.
121	High-Frequency Refined Mamba with Snake Perception Attention for More Accurate Crack Segmentation	E2	General/other; crack	RGB	A2	T3	F0	S1	M1	EV:NR | PD:Y | PC:NR | QGT:NR | CD:NR | FE:NR	Three public crack-segmentation datasets; no real-world deployment reported. Publisher or author record examined; coding was limited to explicitly reported information.
122	Enhancing Pixel-Level Crack Segmentation with Visual Mamba and Convolutional Networks	E2	General/other; crack	RGB	A2	T3	F0	S1	M1	EV:NR | PD:Y | PC:NR | QGT:NR | CD:NR | FE:NR	Two open crack-segmentation datasets; pixel-level segmentation only. Publisher or author record examined; coding was limited to explicitly reported information.
123	Enhancing Bridge Damage Detection with Mamba-Enhanced HRNet for Semantic Segmentation	E2	Bridge/deck; multi-defect/damage	RGB	A2	T3	F0	S1	M1	EV:NR | PD:NR | PC:NR | QGT:NR | CD:NR | FE:NR	Full text examined; coding was limited to explicitly reported information.
124	Layered Bilateral Feature Fusion Network for End-to-End Defect Segmentation on Aging Tiled Building Façades	E1	Building façade/envelope; multi-defect/damage	RGB	A2	T3	F2	S1	M2	EV:NR | PD:NR | PC:NR | QGT:NR | CD:NR | FE:Y	Full text examined; coding was limited to explicitly reported information.
125	DenseFuse: A Fusion Approach to Infrared and Visible Images	E3	General/other; N/A or non-defect target	IRT	N/A	N/A	N/A	N/A	N/A	EV:N/A | PD:N/A | PC:N/A | QGT:N/A | CD:N/A | FE:N/A	Enabling or contextual source; included in corpus stratification but excluded from the 106-study visual-technical denominator. Full text examined; coding was limited to explicitly reported information.
126	FusionGAN: A Generative Adversarial Network for Infrared and Visible Image Fusion	E3	General/other; N/A or non-defect target	IRT	N/A	N/A	N/A	N/A	N/A	EV:N/A | PD:N/A | PC:N/A | QGT:N/A | CD:N/A | FE:N/A	Enabling or contextual source; included in corpus stratification but excluded from the 106-study visual-technical denominator. Full text examined; coding was limited to explicitly reported information.
127	IFCNN: A General Image Fusion Framework Based on Convolutional Neural Network	E3	General/other; N/A or non-defect target	RGB	N/A	N/A	N/A	N/A	N/A	EV:N/A | PD:N/A | PC:N/A | QGT:N/A | CD:N/A | FE:N/A	Enabling or contextual source; included in corpus stratification but excluded from the 106-study visual-technical denominator. Full text examined; coding was limited to explicitly reported information.
128	U2Fusion: A Unified Unsupervised Image Fusion Network	E3	General/other; N/A or non-defect target	RGB	N/A	N/A	N/A	N/A	N/A	EV:N/A | PD:N/A | PC:N/A | QGT:N/A | CD:N/A | FE:N/A	Enabling or contextual source; included in corpus stratification but excluded from the 106-study visual-technical denominator. Full text examined; coding was limited to explicitly reported information.

Ref.	Source (manuscript order)	Evidence	Object / target	Modality	A	T	F	S	M	Evidence-strength flags	Coding note / verification basis
129	NestFuse: An Infrared and Visible Image Fusion Architecture Based on Nest Connection and Spatial/Channel Attention Models	E3	General/other; N/A or non-defect target	IRT	N/A	N/A	N/A	N/A	N/A	EV:N/A | PD:N/A | PC:N/A | QGT:N/A | CD:N/A | FE:N/A	Enabling or contextual source; included in corpus stratification but excluded from the 106-study visual-technical denominator. Full text examined; coding was limited to explicitly reported information.
130	RFN-Nest: An End-to-End Residual Fusion Network for Infrared and Visible Images	E3	General/other; N/A or non-defect target	IRT	N/A	N/A	N/A	N/A	N/A	EV:N/A | PD:N/A | PC:N/A | QGT:N/A | CD:N/A | FE:N/A	Enabling or contextual source; included in corpus stratification but excluded from the 106-study visual-technical denominator. Full text examined; coding was limited to explicitly reported information.
131	SwinFusion: Cross-Domain Long-Range Learning for General Image Fusion via Swin Transformer	E3	General/other; N/A or non-defect target	RGB	N/A	N/A	N/A	N/A	N/A	EV:N/A | PD:N/A | PC:N/A | QGT:N/A | CD:N/A | FE:N/A	Enabling or contextual source; included in corpus stratification but excluded from the 106-study visual-technical denominator. Full text examined; coding was limited to explicitly reported information.
132	DeepFuse: A Deep Unsupervised Approach for Exposure Fusion with Extreme Exposure Image Pairs	E3	General/other; N/A or non-defect target	RGB	N/A	N/A	N/A	N/A	N/A	EV:N/A | PD:N/A | PC:N/A | QGT:N/A | CD:N/A | FE:N/A	Enabling or contextual source; included in corpus stratification but excluded from the 106-study visual-technical denominator. Full text examined; coding was limited to explicitly reported information.
133	Bayesian Fusion for Infrared and Visible Images	E3	General/other; N/A or non-defect target	IRT	N/A	N/A	N/A	N/A	N/A	EV:N/A | PD:N/A | PC:N/A | QGT:N/A | CD:N/A | FE:N/A	Enabling or contextual source; included in corpus stratification but excluded from the 106-study visual-technical denominator. Full text examined; coding was limited to explicitly reported information.
134	Image Fusion in the Loop of High-Level Vision Tasks: A Semantic-Aware Real-Time Infrared and Visible Image Fusion Network	E3	General/other; N/A or non-defect target	IRT	N/A	N/A	N/A	N/A	N/A	EV:N/A | PD:N/A | PC:N/A | QGT:N/A | CD:N/A | FE:N/A	Enabling or contextual source; included in corpus stratification but excluded from the 106-study visual-technical denominator. Full text examined; coding was limited to explicitly reported information.
135	PAIF: Perception-Aware Infrared-Visible Image Fusion for Attack-Tolerant Semantic Segmentation	E3	General/other; N/A or non-defect target	IRT	N/A	N/A	N/A	N/A	N/A	EV:N/A | PD:N/A | PC:N/A | QGT:N/A | CD:N/A | FE:N/A	Enabling or contextual source; included in corpus stratification but excluded from the 106-study visual-technical denominator. Full text examined; coding was limited to explicitly reported information.
136	RTFNet: RGB-Thermal Fusion Network for Semantic Segmentation of Urban Scenes	E3	General/other; thermal/moisture anomaly	IRT	N/A	N/A	N/A	N/A	N/A	EV:N/A | PD:N/A | PC:N/A | QGT:N/A | CD:N/A | FE:N/A	Enabling or contextual source; included in corpus stratification but excluded from the 106-study visual-technical denominator. Full text examined; coding was limited to explicitly reported information.
137	SGFNet: Semantic-Guided Fusion Network for RGB-Thermal Semantic Segmentation	E3	General/other; thermal/moisture anomaly	IRT	N/A	N/A	N/A	N/A	N/A	EV:N/A | PD:N/A | PC:N/A | QGT:N/A | CD:N/A | FE:N/A	Enabling or contextual source; included in corpus stratification but excluded from the 106-study visual-technical denominator. Full text examined; coding was limited to explicitly reported information.
138	TSTR: A Real-Time RGB-Thermal Semantic Segmentation Model with Multimodal Fusion Transformers	E3	General/other; thermal/moisture anomaly	IRT	N/A	N/A	N/A	N/A	N/A	EV:N/A | PD:N/A | PC:N/A | QGT:N/A | CD:N/A | FE:N/A	Enabling or contextual source; included in corpus stratification but excluded from the 106-study visual-technical denominator. Full text examined; coding was limited to explicitly reported information.
139	CMX: Cross-Modal Fusion for RGB-X Semantic Segmentation with Transformers	E3	General/other; N/A or non-defect target	RGB	N/A	N/A	N/A	N/A	N/A	EV:N/A | PD:N/A | PC:N/A | QGT:N/A | CD:N/A | FE:N/A	Enabling or contextual source; included in corpus stratification but excluded from the 106-study visual-technical denominator. Full text examined; coding was limited to explicitly reported information.
140	A Graph Learning Approach for Automatic Subsurface Defects Segmentation in Building Façade Using RGB and Infrared Images	E1	Building façade/envelope; multi-defect/damage	RGB+IRT	A2	T3	F2	S1	M2	EV:NR | PD:NR | PC:NR | QGT:NR | CD:NR | FE:Y	Full text examined; coding was limited to explicitly reported information.
141	Comparison of Multimodal RGB-Thermal Fusion Techniques for Exterior Wall Multi-Defect Detection	E1	Building façade/envelope; thermal/moisture anomaly	RGB+IRT	A2	T3	F3	S1	M2	EV:NR | PD:NR | PC:NR | QGT:NR | CD:NR | FE:Y	Evaluates early, intermediate, and late fusion; F3 records the highest evaluated fusion level. Full text examined; coding was limited to explicitly reported information.
142	A U-Net Convolutional Neural Network Deep Learning Model Application for Identification of Energy Loss in Infrared Thermographic Images	E1	General/other; thermal/moisture anomaly	IRT	A2	T3	F0	S1	M2	EV:NR | PD:NR | PC:NR | QGT:NR | CD:NR | FE:Y	Full text examined; coding was limited to explicitly reported information.
143	Automatic Curtain Wall Frame Detection Based on Deep Learning and Cross-Modal Feature Fusion	E3	General/other; N/A or non-defect target	RGB	N/A	N/A	N/A	N/A	N/A	EV:N/A | PD:N/A | PC:N/A | QGT:N/A | CD:N/A | FE:N/A	Enabling or contextual source; included in corpus stratification but excluded from the 106-study visual-technical denominator. Full text examined; coding was limited to explicitly reported information.
144	Cross-Modal Object Detection for UAV-Based Multispectral Delamination Assessment of Building Envelopes	E1	Building façade/envelope; delamination/debonding	RGB+IRT	A1	T2	F2	S1	M2	EV:NR | PD:NR | PC:NR | QGT:NR | CD:NR | FE:Y	Full text examined; coding was limited to explicitly reported information.

Ref.	Source (manuscript order)	Evidence	Object / target	Modality	A	T	F	S	M	Evidence-strength flags	Coding note / verification basis
145	Edge Intelligence: Paving the Last Mile of Artificial Intelligence With Edge Computing	E3	General/other; N/A or non-defect target	RGB	N/A	N/A	N/A	N/A	N/A	EV:N/A | PD:N/A | PC:N/A | QGT:N/A | CD:N/A | FE:N/A	Enabling or contextual source; included in corpus stratification but excluded from the 106-study visual-technical denominator. Full text examined; coding was limited to explicitly reported information.
146	TUNI: Real-Time RGB-T Semantic Segmentation with Unified Multi-Modal Feature Extraction and Cross-Modal Feature Fusion	E3	General/other; N/A or non-defect target	RGB	N/A	N/A	N/A	N/A	N/A	EV:N/A | PD:N/A | PC:N/A | QGT:N/A | CD:N/A | FE:N/A	Enabling or contextual source; included in corpus stratification but excluded from the 106-study visual-technical denominator. Bibliographic record and reported source scope examined.
147	An Automatic Building Façade Deterioration Detection System Using Infrared-Visible Image Fusion and Deep Learning	E1	Building façade/envelope; multi-defect/damage	RGB+IRT	A2	T3	F1	S1	M2	EV:NR | PD:NR | PC:NR | QGT:NR | CD:NR | FE:Y	Full text examined; coding was limited to explicitly reported information.
148	Fusion of UAV-Based Infrared and Visible Images for Thermal Leakage Map Generation of Building Facades	E1	Building façade/envelope; thermal/moisture anomaly	RGB+IRT+SfM/MVS	A2	T5	F2	S2	M2	EV:NR | PD:NR | PC:NR | QGT:Y | CD:NR | FE:Y	Full text examined; coding was limited to explicitly reported information.
149	Multi-Modal Correlation for UAV-Based Infrared Thermography Diagnosis of Facade Delamination	E1	Building façade/envelope; delamination/debonding	RGB+IRT	A2	T4	F3	S1	M2	EV:NR | PD:NR | PC:NR | QGT:Y | CD:NR | FE:Y	Full text examined; coding was limited to explicitly reported information.
150	UAV-Based Image and LiDAR Fusion for Pavement Crack Segmentation	E2	Road/pavement; crack	RGB+LiDAR	A2	T3	F1	S1	M2	EV:NR | PD:NR | PC:NR | QGT:NR | CD:NR | FE:Y	Full text examined; coding was limited to explicitly reported information.
151	Delamination Detection in Concrete Decks Using Numerical Simulation and UAV-Based Infrared Thermography with Deep Learning	E2	Bridge/deck; delamination/debonding	IRT+simulation	A2	T3	F1	S1	M2	EV:NR | PD:NR | PC:NR | QGT:NR | CD:NR | FE:Y	Full text examined; coding was limited to explicitly reported information.
152	DMM: Disparity-Guided Multispectral Mamba for Oriented Object Detection in Remote Sensing	E3	General/other; N/A or non-defect target	RGB	N/A	N/A	N/A	N/A	N/A	EV:N/A | PD:N/A | PC:N/A | QGT:N/A | CD:N/A | FE:N/A	Enabling or contextual source; included in corpus stratification but excluded from the 106-study visual-technical denominator. Full text examined; coding was limited to explicitly reported information.
153	MambaRefine-YOLO: A Dual-Modality Small Object Detector for UAV Imagery	E3	General/other; N/A or non-defect target	UAV RGB	N/A	N/A	N/A	N/A	N/A	EV:N/A | PD:N/A | PC:N/A | QGT:N/A | CD:N/A | FE:N/A	Enabling or contextual source; included in corpus stratification but excluded from the 106-study visual-technical denominator. Full text examined; coding was limited to explicitly reported information.
154	3D Gaussian Splatting for Real-Time Radiance Field Rendering	E3	General/other; N/A or non-defect target	3D/point cloud	N/A	N/A	N/A	N/A	N/A	EV:N/A | PD:N/A | PC:N/A | QGT:N/A | CD:N/A | FE:N/A	Enabling or contextual source; included in corpus stratification but excluded from the 106-study visual-technical denominator. Bibliographic record and reported source scope examined.
155	Improved Building Facade Segmentation through Digital Twin-Enabled RandLA-Net with Empirical Intensity Correction Model	E3	Building façade/envelope; N/A or non-defect target	RGB	N/A	N/A	N/A	N/A	N/A	EV:N/A | PD:N/A | PC:N/A | QGT:N/A | CD:N/A | FE:N/A	Enabling or contextual source; included in corpus stratification but excluded from the 106-study visual-technical denominator. Full text examined; coding was limited to explicitly reported information.
156	Automated Building Facade Quality Assessment Using 3D Laser Scanning and Improved K-d Tree–Accelerated Point-Cloud Analysis	E1	Building façade/envelope; N/A or non-defect target	TLS/LiDAR	N/A	T4	F0	S2	M2	EV:NR | PD:NR | PC:NR | QGT:Y | CD:NR | FE:Y	Full text examined; coding was limited to explicitly reported information.
157	Automated Recognition of 3D CAD Model Objects and Calculation of As-Built Dimensions for Dimensional Compliance Control in Construction	E3	General/other; N/A or non-defect target	3D/point cloud	N/A	N/A	N/A	N/A	N/A	EV:N/A | PD:N/A | PC:N/A | QGT:N/A | CD:N/A | FE:N/A	Enabling or contextual source; included in corpus stratification but excluded from the 106-study visual-technical denominator. Full text examined; coding was limited to explicitly reported information.
158	Automatic Extraction of Building Features from Terrestrial Laser Scanning	E3	Building; N/A or non-defect target	3D/point cloud	N/A	N/A	N/A	N/A	N/A	EV:N/A | PD:N/A | PC:N/A | QGT:N/A | CD:N/A | FE:N/A	Enabling or contextual source; included in corpus stratification but excluded from the 106-study visual-technical denominator. Bibliographic record and reported source scope examined.
159	3D Is Here: Point Cloud Library (PCL)	E3	General/other; N/A or non-defect target	3D/point cloud	N/A	N/A	N/A	N/A	N/A	EV:N/A | PD:N/A | PC:N/A | QGT:N/A | CD:N/A | FE:N/A	Enabling or contextual source; included in corpus stratification but excluded from the 106-study visual-technical denominator. Full text examined; coding was limited to explicitly reported information.
160	Building and Infrastructure Defect Detection and Visualization Using Drone and Deep Learning Technologies	E2	General structure/concrete; multi-defect/damage	RGB photogrammetry+3D features	A2*	T5	F1	S2	M2	EV:NR | PD:NR | PC:NR | QGT:NR | CD:NR | FE:Y	A2* denotes dense point-level labels (3D analogue of a pixel mask). Full text examined; coding was limited to explicitly reported information.
161	Automated UAV Image-to-BIM Registration for Building Façade Inspection Using Improved Generalised Hough Transform	E1	Building façade/envelope; N/A or non-defect target	UAV RGB+BIM	N/A	T5	F1	S3	M2	EV:NR | PD:NR | PC:NR | QGT:Y | CD:NR | FE:Y	Full text examined; coding was limited to explicitly reported information.

Ref.	Source (manuscript order)	Evidence	Object / target	Modality	A	T	F	S	M	Evidence-strength flags	Coding note / verification basis
162	Automatic Thermographic and RGB Texture of As-Built BIM for Energy Rehabilitation Purposes	E3	General/other; N/A or non-defect target	IRT	N/A	N/A	N/A	N/A	N/A	EV:N/A | PD:N/A | PC:N/A | QGT:N/A | CD:N/A | FE:N/A	Enabling or contextual source; included in corpus stratification but excluded from the 106-study visual-technical denominator. Full text examined; coding was limited to explicitly reported information.
163	AI-Empowered Digital Twin Modeling for High-Precision Building Defect Management Integrating UAV and GeoBIM	E1	Building; multi-defect/damage	UAV RGB+GeoBIM	A1	T5	F1	S3	M2	EV:NR | PD:NR | PC:NR | QGT:Y | CD:NR | FE:Y	Full text examined; coding was limited to explicitly reported information.
164	Uncertainty Quantification Framework for Aerial and UAV Photogrammetry through Error Propagation	E3	General/other; N/A or non-defect target	UAV RGB	N/A	N/A	N/A	N/A	N/A	EV:N/A | PD:N/A | PC:N/A | QGT:N/A | CD:N/A | FE:N/A	Enabling or contextual source; included in corpus stratification but excluded from the 106-study visual-technical denominator. Bibliographic record and reported source scope examined.
165	Mapping and Modelling Defect Data from UAV Captured Images to BIM for Building External Wall Inspection	E1	Building; multi-defect/damage	UAV RGB+BIM	A2	T5	F1	S3	M2	EV:NR | PD:NR | PC:NR | QGT:Y | CD:NR | FE:Y	Full text examined; coding was limited to explicitly reported information.
166	Information Modeling of Earthquake-Damaged Reinforced Concrete Structures	E3	General structure/concrete; multi-defect/damage	RGB	N/A	N/A	N/A	N/A	N/A	EV:N/A | PD:N/A | PC:N/A | QGT:N/A | CD:N/A | FE:N/A	Enabling or contextual source; included in corpus stratification but excluded from the 106-study visual-technical denominator. Full text examined; coding was limited to explicitly reported information.
167	From Pixel to Infrastructure: Photogrammetry-Based Tunnel Crack Digitalization and Documentation Method Using Deep Learning	E2	Tunnel; crack	RGB+SfM/MVS	A2	T5	F1	S2	M2	EV:NR | PD:NR | PC:NR | QGT:Y | CD:NR | FE:Y	Full text examined; coding was limited to explicitly reported information.
168	Deep Learning-Based 3D Reconstruction of Ancient Buildings with Surface Damage Identification and Localization	E1	Building; multi-defect/damage	RGB+SfM/MVS	A2	T5	F1	S2	M2	EV:NR | PD:NR | PC:NR | QGT:Y | CD:NR | FE:Y	Full text examined; coding was limited to explicitly reported information.
169	PointNet: Deep Learning on Point Sets for 3D Classification and Segmentation	E3	General/other; N/A or non-defect target	3D/point cloud	N/A	N/A	N/A	N/A	N/A	EV:N/A | PD:N/A | PC:N/A | QGT:N/A | CD:N/A | FE:N/A	Enabling or contextual source; included in corpus stratification but excluded from the 106-study visual-technical denominator. Full text examined; coding was limited to explicitly reported information.
170	PointNet++: Deep Hierarchical Feature Learning on Point Sets in a Metric Space	E3	General/other; N/A or non-defect target	RGB	N/A	N/A	N/A	N/A	N/A	EV:N/A | PD:N/A | PC:N/A | QGT:N/A | CD:N/A | FE:N/A	Enabling or contextual source; included in corpus stratification but excluded from the 106-study visual-technical denominator. Full text examined; coding was limited to explicitly reported information.
171	RandLA-Net: Efficient Semantic Segmentation of Large-Scale Point Clouds	E3	General/other; N/A or non-defect target	3D/point cloud	N/A	N/A	N/A	N/A	N/A	EV:N/A | PD:N/A | PC:N/A | QGT:N/A | CD:N/A | FE:N/A	Enabling or contextual source; included in corpus stratification but excluded from the 106-study visual-technical denominator. Bibliographic record and reported source scope examined.
172	Image-Based Automated 3D Crack Detection for Post-Disaster Building Assessment	E1	Building; crack	RGB+3D mesh	A2	T5	F1	S2	M1	EV:NR | PD:NR | PC:NR | QGT:Y | CD:NR | FE:NR	Full text examined; coding was limited to explicitly reported information.
173	Multi-Feature Driven Rapid Inspection of Earthquake-Induced Damage on Building Facades Using UAV-Derived Point Cloud	E1	Building façade/envelope; multi-defect/damage	UAV-derived point cloud	A2*	T5	F0	S2	M2	EV:Y | PD:NR | PC:NR | QGT:Y | CD:NR | FE:Y	A2* denotes dense point-level labels (3D analogue of a pixel mask). Full text examined; coding was limited to explicitly reported information.
174	3D Modeling and Automated Measurement of Concrete Cracks via Segment Anything Refinement and Visual Inertial LiDAR Fusion	E2	General structure/concrete; crack	RGB+LiDAR+IMU	A2	T5	F2	S2	M2	EV:Y | PD:NR | PC:Y | QGT:Y | CD:Y | FE:Y	Full text examined; coding was limited to explicitly reported information.
175	Building Information Modeling (BIM) for Existing Buildings — Literature Review and Future Needs	E4	BIM for existing buildings (review)	BIM/digital twin (review)	N/A	N/A	N/A	N/A	N/A	EV:N/A | PD:N/A | PC:N/A | QGT:N/A | CD:N/A | FE:N/A	Enabling or contextual source; included in corpus stratification but excluded from the 106-study visual-technical denominator. Full text examined; coding was limited to explicitly reported information.
176	Geometric Accuracy of Digital Twins for Structural Health Monitoring	E3	General structure/concrete; N/A or non-defect target	RGB	N/A	N/A	N/A	N/A	N/A	EV:N/A | PD:N/A | PC:N/A | QGT:N/A | CD:N/A | FE:N/A	Enabling or contextual source; included in corpus stratification but excluded from the 106-study visual-technical denominator. Full text examined; coding was limited to explicitly reported information.
177	Automated Construction Progress Measurement Using a 4D Building Information Model and 3D Data	E3	Building; N/A or non-defect target	3D/point cloud	N/A	N/A	N/A	N/A	N/A	EV:N/A | PD:N/A | PC:N/A | QGT:N/A | CD:N/A | FE:N/A	Enabling or contextual source; included in corpus stratification but excluded from the 106-study visual-technical denominator. Full text examined; coding was limited to explicitly reported information.
178	Automated Progress Monitoring Using Unordered Daily Construction Photographs and IFC-Based Building Information Models	E3	Building; N/A or non-defect target	RGB	N/A	N/A	N/A	N/A	N/A	EV:N/A | PD:N/A | PC:N/A | QGT:N/A | CD:N/A | FE:N/A	Enabling or contextual source; included in corpus stratification but excluded from the 106-study visual-technical denominator. Full text examined; coding was limited to explicitly reported information.

Ref.	Source (manuscript order)	Evidence	Object / target	Modality	A	T	F	S	M	Evidence-strength flags	Coding note / verification basis
179	Drone-Based AI and 3D Reconstruction for Digital Twin Augmentation	E1	General/other; N/A or non-defect target	UAV RGB+3D digital twin	A1	T5	F1	S3	M1	EV:NR | PD:NR | PC:NR | QGT:NR | CD:NR | FE:NR	Building digital-twin proof of concept; the proceedings DOI is preferred over the arXiv identifier. Peer-reviewed proceedings record and author preprint examined.
180	Intelligent Inspection of Building Exterior Walls Using UAV and Mixed Reality Based on Man-Machine-Environment System Engineering	E1	Building façade/envelope; N/A or non-defect target	UAV RGB+MR	A1	T5	F3	S3	M2	EV:NR | PD:NR | PC:NR | QGT:NR | CD:NR | FE:Y	Full text examined; coding was limited to explicitly reported information.
181	Advancing Building Facade Inspection: Integration of an Infrared Camera-Equipped Drone and Mixed Reality	E1	Building façade/envelope; N/A or non-defect target	RGB+IRT+MR	A2	T5	F3	S3	M2	EV:NR | PD:NR | PC:NR | QGT:NR | CD:NR | FE:Y	Full text examined; coding was limited to explicitly reported information.
182	Classifying Point Clouds at the Facade Level Using Geometric Features and Deep Learning Networks	E3	Building façade/envelope; N/A or non-defect target	3D/point cloud	N/A	N/A	N/A	N/A	N/A	EV:N/A | PD:N/A | PC:N/A | QGT:N/A | CD:N/A | FE:N/A	Enabling or contextual source; included in corpus stratification but excluded from the 106-study visual-technical denominator. Full text examined; coding was limited to explicitly reported information.
183	Asset Management, Condition Monitoring and Digital Twins: Damage Detection and Virtual Inspection on a Reinforced Concrete Bridge	E2	Bridge/deck; multi-defect/damage	monitoring data+digital twin	N/A	N/A	N/A	S3	M3	EV:NR | PD:NR | PC:NR | QGT:NR | CD:NR | FE:Y	Operational sensor-based bridge digital twin; retained as transferable O&M evidence but excluded from visual A/T/F/S/M distributions. Publisher article and author preprint examined.
184	Grad-CAM: Visual Explanations from Deep Networks via Gradient-Based Localization	E3	General/other; N/A or non-defect target	RGB	N/A	N/A	N/A	N/A	N/A	EV:N/A | PD:N/A | PC:N/A | QGT:N/A | CD:N/A | FE:N/A	Enabling or contextual source; included in corpus stratification but excluded from the 106-study visual-technical denominator. Full text examined; coding was limited to explicitly reported information.
185	Why Should I Trust You? Explaining the Predictions of Any Classifier	E3	General/other; N/A or non-defect target	RGB	N/A	N/A	N/A	N/A	N/A	EV:N/A | PD:N/A | PC:N/A | QGT:N/A | CD:N/A | FE:N/A	Enabling or contextual source; included in corpus stratification but excluded from the 106-study visual-technical denominator. Full text examined; coding was limited to explicitly reported information.
186	A Unified Approach to Interpreting Model Predictions	E3	General/other; N/A or non-defect target	RGB	N/A	N/A	N/A	N/A	N/A	EV:N/A | PD:N/A | PC:N/A | QGT:N/A | CD:N/A | FE:N/A	Enabling or contextual source; included in corpus stratification but excluded from the 106-study visual-technical denominator. Bibliographic record and reported source scope examined.
187	Explainable Artificial Intelligence to Advance Structural Health Monitoring	E4	Explainable AI for SHM (review)	XAI for SHM (review)	N/A	N/A	N/A	N/A	N/A	EV:N/A | PD:N/A | PC:N/A | QGT:N/A | CD:N/A | FE:N/A	Enabling or contextual source; included in corpus stratification but excluded from the 106-study visual-technical denominator. Full text examined; coding was limited to explicitly reported information.
188	MARBLE-DA: Masonry Analysis with Robust, Batch-Normalised, Label-Free, Explainable Domain Adaptation for Crack Detection	E1	Masonry/cladding; crack	RGB	A0	T1	F0	S0	M1	EV:Y | PD:NR | PC:NR | QGT:NR | CD:Y | FE:NR	Full text examined; coding was limited to explicitly reported information.
189	Physics-Informed Machine Learning for Structural Health Monitoring	E4	Physics-informed SHM (review)	Physics-informed SHM (review)	N/A	N/A	N/A	N/A	N/A	EV:N/A | PD:N/A | PC:N/A | QGT:N/A | CD:N/A | FE:N/A	Enabling or contextual source; included in corpus stratification but excluded from the 106-study visual-technical denominator. Full text examined; coding was limited to explicitly reported information.
190	From Traditional Damage Detection Methods to Physics-Informed Machine Learning in Bridges: A Review	E4	Bridge damage identification (review)	Physics-informed SHM (review)	N/A	N/A	N/A	N/A	N/A	EV:N/A | PD:N/A | PC:N/A | QGT:N/A | CD:N/A | FE:N/A	Enabling or contextual source; included in corpus stratification but excluded from the 106-study visual-technical denominator. Full text examined; coding was limited to explicitly reported information.
191	Advancing Structural Failure Analysis with Physics-Informed Machine Learning in Engineering Applications	E4	Structural failure analysis (review)	Physics-informed engineering analysis (review)	N/A	N/A	N/A	N/A	N/A	EV:N/A | PD:N/A | PC:N/A | QGT:N/A | CD:N/A | FE:N/A	Enabling or contextual source; included in corpus stratification but excluded from the 106-study visual-technical denominator. Full text examined; coding was limited to explicitly reported information.
192	Physics-Informed Neural Networks: A Deep Learning Framework for Solving Forward and Inverse Problems Involving Nonlinear Partial Differential Equations	E3	General/other; N/A or non-defect target	RGB	N/A	N/A	N/A	N/A	N/A	EV:N/A | PD:N/A | PC:N/A | QGT:N/A | CD:N/A | FE:N/A	Enabling or contextual source; included in corpus stratification but excluded from the 106-study visual-technical denominator. Full text examined; coding was limited to explicitly reported information.
193	Infrared Thermography of Façade Delamination: Quantifying Environmental Variability and Heat Transfer Mechanisms	E1	Building façade/envelope; delamination/debonding	IRT	N/A	N/A	N/A	N/A	N/A	EV:NR | PD:NR | PC:NR | QGT:Y | CD:NR | FE:Y	Physical/mechanistic façade evidence; excluded from A/T/F/S/M distributions. Full text examined; coding was limited to explicitly reported information.
194	Learning Transferable Visual Models from Natural Language Supervision	E3	General/other; N/A or non-defect target	RGB	N/A	N/A	N/A	N/A	N/A	EV:N/A | PD:N/A | PC:N/A | QGT:N/A | CD:N/A | FE:N/A	Enabling or contextual source; included in corpus stratification but excluded from the 106-study visual-technical denominator. Full text examined; coding was limited to explicitly reported information.

Ref.	Source (manuscript order)	Evidence	Object / target	Modality	A	T	F	S	M	Evidence-strength flags	Coding note / verification basis
195	Visual Instruction Tuning	E3	General/other; N/A or non-defect target	RGB	N/A	N/A	N/A	N/A	N/A	EV:N/A | PD:N/A | PC:N/A | QGT:N/A | CD:N/A | FE:N/A	Enabling or contextual source; included in corpus stratification but excluded from the 106-study visual-technical denominator. Full text examined; coding was limited to explicitly reported information.
196	MiniGPT-4: Enhancing Vision-Language Understanding with Advanced Large Language Models	E3	General/other; N/A or non-defect target	RGB	N/A	N/A	N/A	N/A	N/A	EV:N/A | PD:N/A | PC:N/A | QGT:N/A | CD:N/A | FE:N/A	Enabling or contextual source; included in corpus stratification but excluded from the 106-study visual-technical denominator. Bibliographic record and reported source scope examined.
197	Tailored Vision-Language Framework for Automated Hazard Identification and Report Generation in Construction Sites	E3	General/other; N/A or non-defect target	RGB	N/A	N/A	N/A	N/A	N/A	EV:N/A | PD:N/A | PC:N/A | QGT:N/A | CD:N/A | FE:N/A	Enabling or contextual source; included in corpus stratification but excluded from the 106-study visual-technical denominator. Full text examined; coding was limited to explicitly reported information.
198	Datasheets for Datasets	E4	Dataset documentation framework	N/A	N/A	N/A	N/A	N/A	N/A	EV:N/A | PD:N/A | PC:N/A | QGT:N/A | CD:N/A | FE:N/A	Enabling or contextual source; included in corpus stratification but excluded from the 106-study visual-technical denominator. Bibliographic record and reported source scope examined.
199	Model Cards for Model Reporting	E4	Model-reporting framework	N/A	N/A	N/A	N/A	N/A	N/A	EV:N/A | PD:N/A | PC:N/A | QGT:N/A | CD:N/A | FE:N/A	Enabling or contextual source; included in corpus stratification but excluded from the 106-study visual-technical denominator. Bibliographic record and reported source scope examined.
200	Domain Generalization: A Survey	E4	Domain generalization (review)	N/A	N/A	N/A	N/A	N/A	N/A	EV:N/A | PD:N/A | PC:N/A | QGT:N/A | CD:N/A | FE:N/A	Enabling or contextual source; included in corpus stratification but excluded from the 106-study visual-technical denominator. Bibliographic record and reported source scope examined.
201	Can You Trust Your Model's Uncertainty? Evaluating Predictive Uncertainty under Dataset Shift	E3	General/other; N/A or non-defect target	RGB	N/A	N/A	N/A	N/A	N/A	EV:N/A | PD:N/A | PC:N/A | QGT:N/A | CD:N/A | FE:N/A	Enabling or contextual source; included in corpus stratification but excluded from the 106-study visual-technical denominator. Bibliographic record and reported source scope examined.
202	An Introduction to Multisensor Data Fusion	E4	Multisensor data fusion (overview)	Multisensor data fusion (review)	N/A	N/A	N/A	N/A	N/A	EV:N/A | PD:N/A | PC:N/A | QGT:N/A | CD:N/A | FE:N/A	Enabling or contextual source; included in corpus stratification but excluded from the 106-study visual-technical denominator. Full text examined; coding was limited to explicitly reported information.
203	An Introduction to Structural Health Monitoring	E4	Structural health monitoring (overview)	Multiple SHM modalities (overview)	N/A	N/A	N/A	N/A	N/A	EV:N/A | PD:N/A | PC:N/A | QGT:N/A | CD:N/A | FE:N/A	Enabling or contextual source; included in corpus stratification but excluded from the 106-study visual-technical denominator. Full text examined; coding was limited to explicitly reported information.
